# Effects of larval exposure to the insecticide flumethrin on the development of honeybee (*Apis mellifera*) workers

**DOI:** 10.3389/fphys.2022.1054769

**Published:** 2022-12-14

**Authors:** Chen Liu, Xiaobo Wu, Heyan Yang, Longtao Yu, Yong Zhang

**Affiliations:** ^1^ Honeybee Research Institute, Jiangxi Agricultural University, Nanchang, China; ^2^ Jiangxi Province Key Laboratory of Honeybee Biology and Beekeeping, Nanchang, China

**Keywords:** flumethrin, Apis mellifera, enzyme, toxicity, transcriptome

## Abstract

Flumethrin is a widely used acaricide, but its improper use often leads to residue accumulation in honeybee colonies, thus threatening the health of honeybees, especially at the larval stage. Therefore, this study aimed to describe the direct toxicity of flumethrin on honeybee (*Apis mellifera*) larvae by conducting bioassays for immune and detoxification-related enzymes and transcriptome sequencing to determine the potential effects on newly emerged adults who were exposed to flumethrin during the larval stage. Results showed that the higher the concentration of flumethrin the honeybee larvae were exposed to, the greater the damage to the physiology of honeybee larvae and the newly emerged worker bees. When honeybee larvae were exposed to flumethrin concentrations higher than 0.01 mg/L, the activities of glutathione sulfur transferase and carboxylesterase were affected, and the metabolism-related genes in the head of newly emerged honeybees exposed to flumethrin during the larval stage were down-regulated. Flumethrin concentration higher than 0.1 mg/L significantly increased mixed-functional oxidase content in honeybee larvae, reduced the larval survival rate, and down-regulated the expression levels of olfactory-related and antioxidant-related genes in newly emerged honeybees. Furthermore, a flumethrin concentration of 1 mg/L significantly down-regulated the expression levels of immune and detoxification-related genes in newly emerged honeybees. These findings provide a comprehensive understanding of the response of honeybee larvae to sublethal flumethrin toxicity and could be used to further investigate the complex molecular mechanisms in honeybees under pesticide stress.

## 1 Introduction

The honeybee is a very important pollination insect (Kevan et al., 1990). It has huge economic value in global agriculture and plays an important role in maintaining ecological diversity (Potts et al., 2010). The *Varroa* mite is one of the most threatening pests in apiculture, causing huge economic losses to apiculture worldwide (Ghasemi et al., 2011). In honeybee colonies, *Varroa* mites damage the health of honeybees by sucking the fat and hemolymph from honeybee larvae and adults (Ramsey et al., 2019). At present, acaricides, such as flumethrin, are commonly used to control *Varroa* mites. Flumethrin is a second-generation pyrethroid insecticide used against *Varroa* mites through application within honeybee hives. Its main insecticidal mechanism is to delay the closure of ion channels and interfere with the normal physiological functions of the nervous systems of insects (Lund and Narahashi, 1981), causing symptoms such as excitement, convulsions, spasms, and even death (Catterall, 2001; Soderlund et al., 2002; Raymond-Delpech et al., 2005; Shafer et al., 2005). Flumethrin kills the mites but also affects the health of honeybees. When insecticides are used according to the manufacturer’s instructions, latent toxin interactions from insecticides and fungicides can affect the health of honeybee colonies and have sublethal effects in honeybees (Frost et al., 2012). In recent years, researchers have been paying attention to sublethal doses or concentrations of pesticides. Pesticides administered below the sublethal dose can still increase the susceptibility of honeybee to pathogens, cause certain damage to the learning, memory, and behavioral abilities of honeybees, and affect the development of the whole honeybee colony (El Hassani et al., 2005; Wu et al., 2011; Gill et al., 2012; Frost et al., 2012; Williamson et al., 2013; Doublet et al., 2015).

The adverse effects of flumethrin on honeybees through multiple life stages are well documented. Honeybee (*A. mellifera*) larvae exposed to flumethrin have been shown to have increased mortality, developmental failure, physiological changes, and low immunity in larval, pupal, and adult stages (Qi et al., 2020). Exposure of honeybee larvae to a sublethal dose of flumethrin has been shown to affect larval development, pupation and adult emergence rates, and the activities of key enzymes and related gene expression levels of *A. mellifera* (Niu, 2019). The toxic stress on honeybee larvae caused by flumethrin is alleviated by microorganisms, which play a role in intestinal barrier function and protect the larvae to some extent (Ramya et al., 2016). In addition, honeybee larvae can activate immune and detoxification systems to defend against the threat of increasing flumethrin concentrations (Yu et al., 2021). Flumethrin was shown to adversely affect the lifespan, immune function, and forage behavior of worker bees (*A. mellifera*) (Wu et al., 2023), and chronic exposure to sublethal concentrations of flumethrin was shown to negatively affect the development, olfactory learning, and memory of worker bees (*A. mellifera*; Li et al., 2022). The ingestion of a sublethal dose of sugar water containing flumethrin significantly affected the longevity and acquisition behavior of *Apis cernana* (Tan et al., 2013). Flumethrin has also been shown to affect the detoxification pathways of *A. cernana* (Jiang et al., 2016). Together, these studies show how flumethrin can affect honeybee development, immune and detoxification-related enzyme activity, behavior, and lifespan.

Due to the high lipophilic nature of flumethrin, its residues are often a problem in bee products (Niu, 2019). In one study, the residual concentrations of flumethrin detected in beeswax during a honey-flowing period ranged from 0.03 to 0.13 mg/kg, while residual concentrations as high as 3 mg/kg have been detected in older beeswax samples (Bogdanov, 2003; Johnson et al., 2010; Rosenkranz et al., 2010). A previous study reported a detection rate of flumethrin in honey samples of 75.3%, with a maximum concentration of 0.126 mg/kg (Yu et al., 2015). According to regulations put forth by the Chinese agricultural industry, the maximum residue limit (MRL) value of flumethrin in bee products is 0.01 mg/kg (Song et al., 2021). The European Union stipulates that flumethrin cannot be used with animals that provide milk for human consumption (Mutinelli, 2016). Recently, the issue of pesticide residues and their effects on human health have attracted more and more attention. This study aimed to investigate the impact of residual flumethrin levels in bee products on the physiological functions of honeybee larvae and newly emerged honeybees exposed to flumethrin during the larval stage. The findings from this study will fill the knowledge gap related to the chronic effects of flumethrin on honeybee development and provide new ideas for comprehensive risk assessments and application technology for acaricides in beehives.

## 2 Materials and methods

### 2.1 Honeybee and chemicals

Honeybees were obtained from the Honeybee Research Institute of Jiangxi Agricultural University (Jiangxi, China). The sucrose solution containing flumethrin was formulated according to the residual levels of flumethrin in bee products (Bogdanov, 2003; Johnson et al., 2010; Rosenkranz et al., 2010). Flumethrin (98.5% pure) was purchased from ANPEL Laboratory Technologies Inc. (Shanghai, China). Flumethrin was dissolved with acetone then diluted with a 50% sucrose solution (Xilong Scientific, Shantou, China) to get the following concentrations (1, 0.1, and 0.01 mg/L) for the three experimental groups. Sucrose solution (50%) was prepared for the control group.

### 2.2 Larva treatment

A new foundation was put into the experimental hive and the queen was kept to lay eggs on the new comb for 12 h when the new comb was being constructed. After the larvae hatched, they were equally divided into four groups with approximately 100 larvae per group. All larvae were from the same honeybee colony. From 2 days of age, experimental larvae were fed daily with 2 µl of sucrose solution containing flumethrin at concentrations of 1, 0.1, and 0.01 mg/L, while the control group was fed 50% sucrose solution until capped. The number of larvae was recorded daily before feeding. Because worker bees remove any dead larvae, the reduction in larvae count is equal to the number of dead larvae.

### 2.3 Enzyme content assay

Seven-day-old larvae (just capped) were frozen with liquid nitrogen and placed at −80°C for subsequent determination of related enzyme activities. Each sample contained a whole larva, and each group had five replicates. Protein concentrations were measured using a BCA Protein Assay Kit (BL521A, Biosharp, Anhui, China). The activities of glutathione sulfur transferase (GST), carboxylesterase (CarE), and mixed-functional oxidase (MFOs), as well as the total antioxidant capacity (T-AOC), were determined using kits A004-1-1, A33-1-1, H452-1, and A015-1, respectively (Nanjing Jiancheng Bioengineering Institute, China). All assays were performed according to the manufacturer’s instructions.

### 2.4 RNA isolation, library preparation, and sequencing

Honeybee larvae were treated according to the methods in Section 2.2 then were transferred to an incubator maintained at 34.5°C and 75% relative humidity for emerging once they had been capped for 12 days. The newly emerged honeybees were immediately frozen with liquid nitrogen and placed at -80°C for RNA isolation, library preparation, and sequencing. The head of the honeybee were put into an Eppendorf tube with 200 µl TransZoL Up (ER501-01, TransGen Biotech, Beijing, China) and fully ground with an electric micro-tissue crusher. Each tube was filled with 800 µl TransZoL Up. Total RNA was extracted as previously described (Qin et al., 2012). The RNA purity was determined using a nucleic acid protein tester (NanoPhotometerTM P300, Implen GmbH, Munich, Germany). Total RNA was reverse transcribed with a PrimeScript TR Reagent kit according to manufacturer’s instructions (RR047A, Takara Bio, Beijing, China), and the resulting complementary DNA (cDNA) was stored at -80°C. Sequencing work was completed by Novogene using the Illumina NovaSeq 6,000 sequencing platform (Illumina, San Diego, CA, United States).

### 2.5 Raw data acquisition and statistical analysis

After obtaining sequence reads, it was necessary to filter the raw data. Reads with adapter sequences, containing N (indicating that base information cannot be determined), or of low-quality (=20 bases representing more than 50% of the total read length) were removed. The expression level of each gene was normalized as fragments per kilobase of transcript sequence per millions mapped reads (FPKM) based on the gene length and the number of reads mapped to each gene (Mortazavi et al., 2008). An absolute value of log2Ratio ≥1 (twofold change) and false discovery ratio (FDR) ≤ 0.05 were set as significance thresholds and used to filter the differentially expressed genes (DEGs; Anders and Huber, 2012). ClusterProfile software (ver. 3.4.4) was used for Gene Ontology (GO) functional enrichment analysis and Kyoto Encyclopedia of Genes and Genomes (KEGG) pathway analysis of differential gene sets.

### 2.6 Validation by quantitative real-time PCR (qRT-PCR)

Four genes related to immunity, detoxification, and olfaction were analyzed by qRT-PCR with three biological and three technical replicates. These four genes were selected because honeybees resist insecticides through activation of their immune and detoxification pathways; insecticides also cause damage to the nervous system and impair the olfactory ability of honeybees. The gene-specific primers used in this study are presented in Table 1. Glyceraldehyde 3-phosphate dehydrogenase (GAPDH) was used as an internal control gene. Ct values of target and reference genes were collected, and the relative expression levels of each target gene were calculated (Huang et al., 2012).

**TABLE 1:** Gene-specific primers used in quantitative real-time PCR.

Gene name	Forward primer (5′-3′)	Reverse primer (5′-3′)
CSP1	AAC​GTG​GAC​ATA​TGC​TGA​GGA​AC	CCG​GAG​TTA​AAC​AAG​AGC​CTG​C
OBP13	GTC​AGT​TTG​CGC​CGA​AGA​AAA​TGG	ACC​CTT​CTT​AAC​GTC​GTC​TGC​TTT​C
LOC406081	GCT​CGG​TGA​ACA​CTC​CTC​AAC​TG	TGA​TTG​TGA​AGG​TTC​TCG​CCA​ACT​C
LOC406105	GAT​ACC​GTT​CGC​AAC​ACC​TCC​TG	TGG​CTG​ACG​CTG​AGA​GCA​GTA​AT
GAPDH	GCT​GGT​TTC​ATC​GAT​GGT​TT	ACG​ATT​TCG​ACC​ACC​GTA​AC

### 2.7 Data analysis

The Statistical Package for the Social Sciences (SPSS) 17.0 was used to analyze the differences in enzyme activity and gene expression of the four groups. One-way analysis of variance (ANOVA) was used to compare the differences in enzyme activity and gene expression between the treatment and control groups. At *p* < 0.05, the least significant difference (LSD) test was used to determine differences between groups. GraphPad Prism 5 software (GraphPad Software, San Diego, CA, United States) was used to plot the survival curve of honeybee larvae and analyze the differences between each group by Kaplan-Meier analysis.

## 3 Results

### 3.1 Flumethrin affects honeybee larval survival

**FIGURE 1:**
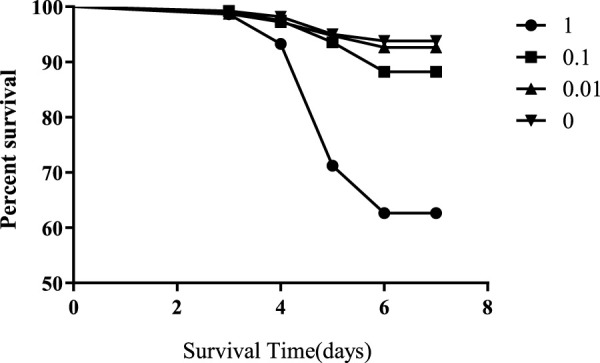
Effect of flumethrin on the survival of honeybee larvae.

Honeybees were exposed to 1, 0.1, 0.01, or 0 mg/L of flumethrin during the larval stage from day 2 to day 5. The mortality rates of honeybee larvae in the 1 and 0.1 mg/L groups were significantly higher than those in the control group (1 mg/L vs control, *p* < 0.0001; 0.1 mg/L vs control, *p* < 0.0001; Figure 1); however, there was no significant difference in the mortality rates of honeybee larvae between the 0.01 mg/L and control groups (*p* = 0.3325).

### 3.2 Effects of flumethrin on immunity and detoxification-related enzyme activities of larvae

**FIGURE 2:**
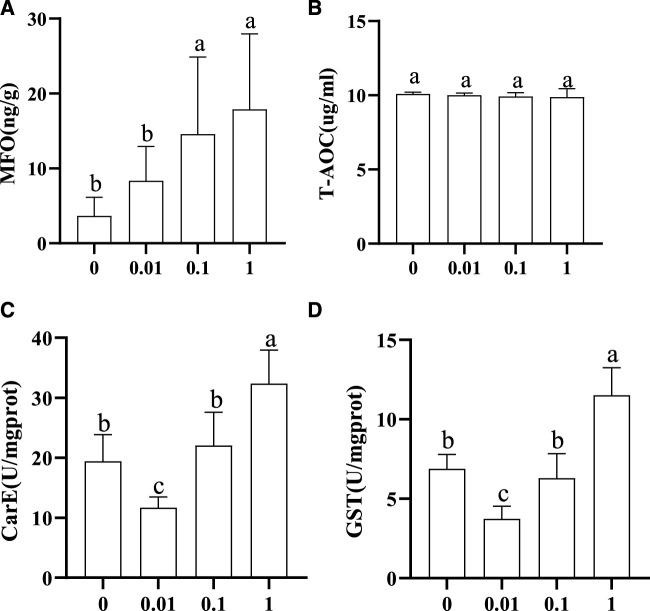
Effect of flumethrin on the content of immune and detoxification-related enzymes in honeybee larvae. The same letter above the bars means no significant difference. **(A–D)** represent the content of MFO, T-AOC, CarE, and Gst measured in each group, respectively.

The activity trends of MFO, GST, CarE, and T-AOC in honeybee larvae exposed to different concentrations of flumethrin are shown in Figure 2. MFO content in the larvae increased gradually with an increase of flumethrin concentration (Figure 2A). The content of MFO in the 1 (17.90 ± 10.06 ng/g) and 0.1 mg/L groups (14.60 ± 10.28 ng/g) was significantly higher than in the 0.01 mg/L (8.344 ± 4.592 ng/g) and control groups (3.666 ± 2.473 ng/g; *p* < 0.05). There was no significant difference in T-AOC among the four groups (Figure 2B). The trends for CarE and GST (Figure 2C, Figure 2D) activities were similar. There was a significant decrease in the activities of GST and CarE in the 0.01 mg/L group compared with the control group (*p* < 0.05). On the other hand, GST and CarE activities in the 1 mg/L group were significantly higher than those in the control group (*p* < 0.05). There were no significant differences in the activities of GST and CarE between the 0.1 mg/L and control groups.

### 3.3 Raw RNA-Seq data analysis

The transcriptome sequencing statistics for all samples are summarized in Supplemental Table S1. The raw reading included 4.03 × 10^7^–4.79 × 10^7^ reads. The clean reading segment included 3.98 × 10^7^–4.71 × 10^7^ reads after removing the invalid reading segments. Among the 11 libraries, more than 96.82% were Q20, and more than 91.83% were Q30. These results indicated that the data quality was good and was therefore used for subsequent analysis. The whole sequencing dataset was deposited in the National Center for Biotechnology Information (NCBI) Sequence Read Archive (accession number: PRJNA866287).

**FIGURE 3:**
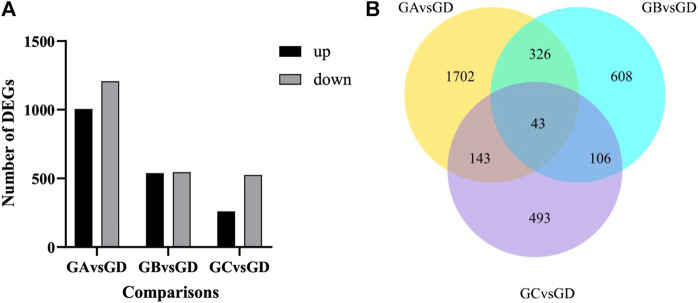
Effect of flumethrin on gene expression of newly emerged honeybees. **(A)** Numbers of DEGs of *A. mellifera* larvae treated with flumethrin. Up- and down-regulation indicates higher or lower expression of these genes in the treatment groups. **(B)** Venn analysis of DEGs. The sum of the numbers in each large circle represents the total number of DEGs in the comparative combinations, while the overlapping part of the circles represents the DEGs shared by the combinations. GA, GB, GC, and GD represent 1, 0.1, 0.01, and 0 mg/L groups, respectively.

As shown in Figure 3A, with the increase in flumethrin concentration, the number of DEGs increased gradually. A total of 785 DEGs were identified between the 0.01 mg/L and the control groups. Of these, 260 were upregulated, and 525 were downregulated. A total of 1,083 DEGs were identified between the 0.1 mg/L and control groups, of which 538 DEGs were upregulated, and 545 DEGs were downregulated. In total, 2,214 DEGs were identified between the 1 mg/L and control groups, of which 1,006 DEGs were upregulated, and 1,208 DEGs were downregulated. The common DEGs are shown in Figure 3B and Table 2. There were 43 common DEGs, including loc102656552, Cox6c, rpl39, loc725432, loc100578551, and Apd-2, in the pesticide treatment group. In addition, as flumethrin concentration increased, more DEGs were produced. An additional 326 common DEGs, including olfactory-related genes (CSP2, CSP3, OBP17, OBP3) and an antioxidant gene (SOD1), were found in the 1 and 0.1 mg/L groups compared with the control group, as shown in Supplemental Table S2 (excluding the DEGs in Table S3). A total of 1,702 unique DEGs, including detoxification-related genes (GSTs1, loc406081) and an antioxidant gene (MsrA), were found in the 1 mg/L group compared with the control group (Supplemental Table S3).

**TABLE 2:** The fragments per kilobase of exon per million mapped fragments (FPKM) of common DEGs between treated groups and control group.

gene_name	1 mg/L	0.1 mg/L	0.01 mg/L	Control	gene_description
LOC100577725	92.55	80.04	131.71	205.50	probable chitinase 10
LOC724114	108.93	139.66	179.12	234.74	uncharacterized LOC724114 transcript variant X2
LOC413048	8.54	10.16	12.30	16.06	bifunctional 3′-phosphoadenosine 5′-phosphosulfate synthase 2 transcript variant X2
Rpl39	79.80	79.10	110.85	154.25	ribosomal protein L39
LOC725432	634.60	739.29	890.81	1123.68	60S ribosomal protein L37a
LOC727025	50.25	49.75	62.43	87.11	DNA-directed RNA polymerases I II and III subunit RPABC4
Cox6c	320.31	296.78	380.02	498.62	cytochrome c oxidase subunit VIc transcript variant X5
LOC102656552	7.10	6.50	8.31	15.48	hypertrehalosaemic prohormone transcript variant X1
LOC551861	309.33	305.35	376.68	490.39	ATP synthase subunit e mitochondrial
LOC100578551	64.42	69.92	89.92	113.99	39S ribosomal protein L34 mitochondrial
LOC413391	2.17	2.24	2.69	3.76	homeobox protein MSX-2
LOC102656354	1135.16	1232.38	1298.24	1717.68	uncharacterized LOC102656354
LOC408525	11.31	13.15	13.64	17.96	neuronal acetylcholine receptor subunit alpha-10
LOC552685	1074.65	1022.31	1330.61	1757.53	pupal cuticle protein
LOC724851	5.70	6.20	6.28	7.92	LIM domain only protein 3 transcript variant X3
LOC412768	15.29	17.66	16.70	24.84	protein takeout
LOC410555	6.80	6.23	7.24	9.74	insulin-like growth factor-binding protein complex acid labile subunit
LOC725548	39.95	36.78	42.34	52.37	protein FAM151B transcript variant X2
LOC100642173	0.66	0.72	0.67	1.14	uncharacterized LOC100642173
Apd-2	7665.40	6357.81	7740.82	12370.28	apidermin 2 transcript variant X1
LOC725415	9.65	9.18	9.08	12.73	BTB/POZ domain-containing protein KCTD16 transcript variant X2
LOC100578090	12.23	10.28	12.37	16.51	uncharacterized LOC100578090
LOC725765	2.33	2.11	2.57	3.42	uncharacterized LOC725765 transcript variant X4
LOC726286	44.42	90.32	107.95	61.27	uncharacterized LOC726286
LOC113218735	2348.87	1956.31	2095.88	3492.02	uncharacterized LOC113218735
LOC410577	11.60	10.82	10.34	15.37	acid sphingomyelinase-like phosphodiesterase 3a transcript variant X3
LOC409058	0.12	0.07	0.10	0.25	L-threonine ammonia-lyase-like transcript variant X2
LOC100576700	27.23	16.52	13.76	42.92	class A basic helix-loop-helix protein 15 transcript variant X2
LOC551369	603.03	460.69	571.13	797.40	actin clone 205
LOC100577548	3.01	2.41	2.65	4.37	tubulin beta chain transcript variant X2
LOC725190	1.06	1.05	0.85	1.46	protein snail transcript variant X2
LOC411564	1.09	8.60	5.79	2.18	nose resistant to fluoxetine protein 6 transcript variant X2
LOC550673	10.90	11.92	12.14	8.92	inosine-5′-monophosphate dehydrogenase 1b
LOC551772	1.71	1.46	1.46	2.36	zeta-sarcoglycan transcript variant X1
LOC102654344	3.23	1.76	1.60	2.50	glutamine-rich protein 2
LOC113219007	3.36	2.84	2.27	4.92	zinc finger protein 524-like
LOC409000	6.69	6.20	5.54	8.41	cell adhesion molecule 2 transcript variant X2
LOC726150	0.04	0.03	0.03	0.11	transcription factor Sox-21-B
LOC100578519	1.97	2.37	2.23	1.54	uncharacterized protein PF11_0207

**FIGURE 4:**
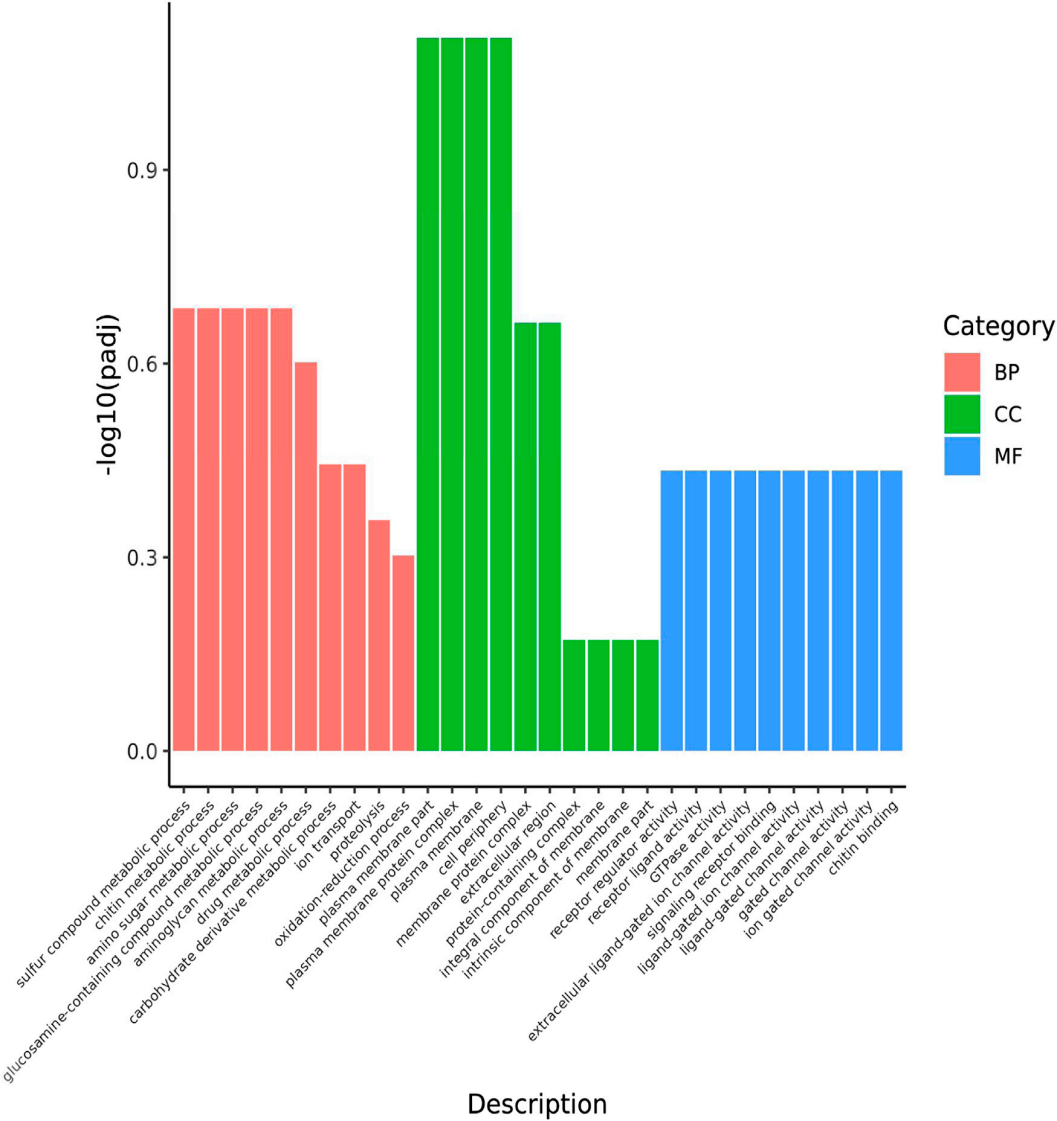
GO classification of DEGs shared by the 3 comparative combinations. The abscissa in the figure is GO terms; the ordinate is the significance level of GO Term enrichment, which is represented by -log10 (padj), and different colors represent different functional categories. BP: DEGs enriched for biological process; CC: DEGs enriched for cellular components; MF: DEGs enriched for molecular function. The same is below.

**FIGURE 5:**
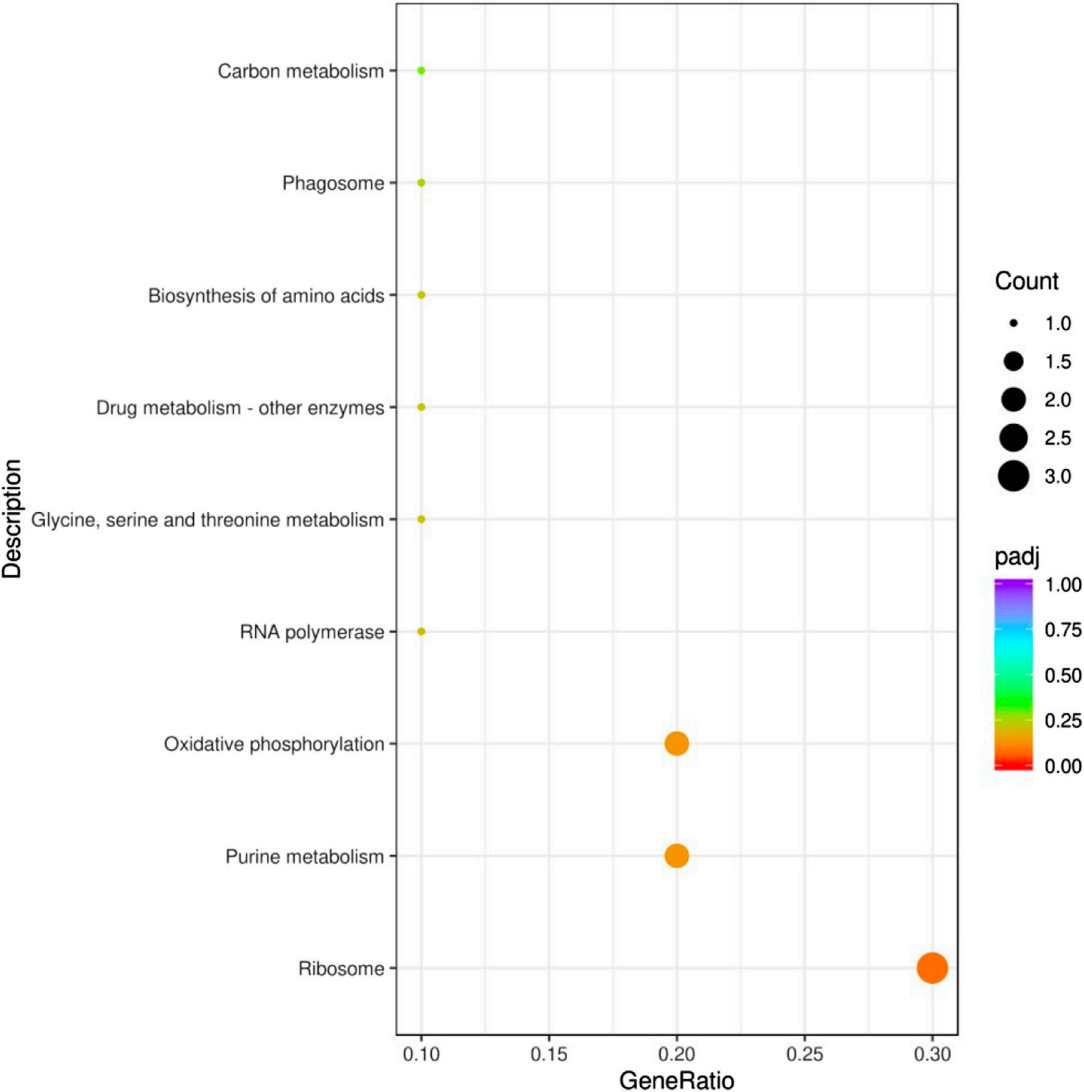
KEGG pathway enrichment analysis of DEGs. The abscissa in the figure is the ratio of the number of differential genes annotated to the KEGG pathway to the total number of differential genes. The ordinate is the KEGG pathway. Count: Number of DEGs annotated to the KEGG pathway. Padj: *p*-value corrected for multiple hypothesis testing.

The functions of the 43 common DEGs in the pesticide group were analyzed according to the GO database. The enriched terms in the biological process category included drug metabolic processes, oxidation-reduction processes, and ion transport. In the cellular component category, the enriched terms included plasma membrane part, plasma membrane protein complex, and membrane part. In the molecular function category, the enriched terms included receptor regulator activity, receptor ligand activity, and GTPase activity (Figure 4). In addition, these 43 common DEGs were related to enrichment in nine KEGG pathways, including ribosome function, purine metabolism, and oxidative phosphorylation (Figure 5).

### 3.4 Validation of target DEGs by qRT-PCR

**FIGURE 6:**
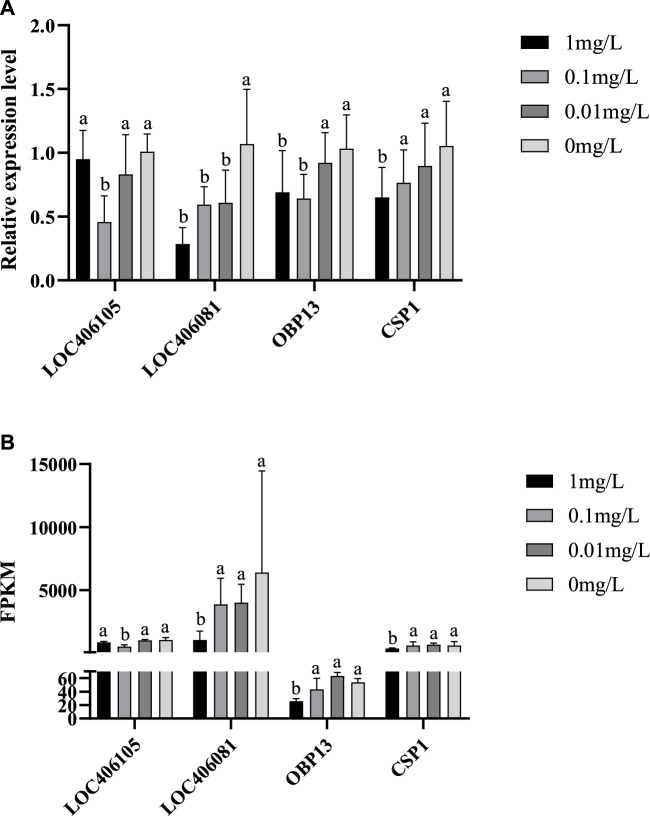
qRT-PCR verification **(A)** and RNA Seq data **(B)** of DEGs on the head of newly emerged honeybees in flumethrin-treated and control groups, respectively.

The expression patterns of the selected 4 DEGs verified by qRT-PCR (Figure 6A) were similar to those of RNA-seq (Figure 6B) indicating that the abundance of the Illumina reads closely mirrored the actual expression levels of the DEGs. The validation results for the 4 DEGs indicated that the qPCR data correlated well with the transcriptome data and further confirmed the significance of flumethrin induced downregulation of genes involved in immunity, detoxification, and olfaction in the heads of honeybees.

## 4 Discussion

Each honeybee goes through four life stages: egg, larva, pupa, and adult. The normal growth and development of larvae are the basis of the entire colony. However, larvae may be exposed to pesticides through activities such as living in contaminated hives and eating food containing pesticide residues, including royal jelly and honey (Tavares et al., 2017). In this study, the detection of immune and detoxification-related enzyme activities and RNA-Seq technology were used to investigate the toxic effects of flumethrin on honeybees.

There are three major immune and detoxification-related enzymes in insects, namely cytochrome P450 monooxygenase (P450), GST, and CarE (Claudianos et al., 2010). The core enzyme system of MFO determined in this study was the cytochrome P450 monooxygenase system. These enzymes have a direct impact on the detoxification and metabolism of insects and participate in the formation of drug resistance in insects (Enayati et al., 2005; Oakeshott et al., 2010). This study showed that the MFO activity increased with the increase in flumethrin concentration (Figure 2A). The MFO activity of larvae in the 1 and 0.1 mg/L groups was significantly higher than in the 0.01 mg/L and control groups, while there was no significant difference in larval MFO content between the 0.01 mg/L and control groups. These results indicate that residual levels of flumethrin (less than 0.01 mg/L) in the larval diet would not affect the activity of MFO. In contrast, MFO content could increase when honeybee larvae encounter residual flumethrin concentrations above 0.1 mg/L and could exert its detoxification effects to decompose toxic substances. With an increasing concentration of flumethrin, the activities of CarE and GST showed an initial decrease followed by a gradual increase (Figure 2C,D). It is possible that the activities of CarE and GST in the 0.01 mg/L group decreased due to using the larva’s CarE and GST enzymes neutralizing the negative effects of flumethrin. When the residual level of flumethrin in the larval diet was higher than 0.01 mg/L, more CarE and GST enzymes were produced to resist the toxic effect of pesticides; the concentration of these two enzymes rises to help prevent any harm to the body caused by flumethrin. Nielsen et al. (2000) measured the detoxification enzyme GST in larvae, pupae, and adult honeybees collected from flumethrin-treated colonies. They found significantly increased GST activities in the late fifth larval and pupal instars. T-AOC is an indicator of the antioxidant capacity in the reactive organism (Ghiselli et al., 2000). In this study, the content of T-AOC was not significantly different among the four treatment groups (Figure 2B), indicating that flumethrin might not affect the T-AOC of honeybee larvae. However, the mechanism should be further investigated.

The DEGs found in the present study reflect the multifaceted influence of flumethrin on honeybee physiology. There were huge differences in the number of DEGs in different dose groups compared with the control group (Figure 3). The number of DEGs increased with the increase in flumethrin concentration in the treatment groups, which showed that the higher the concentration of flumethrin, the greater the impact on and damage to honeybees. We found 43 common DEGs, including loc102656552, Cox6c, rpl39, loc725432, loc100578551, and Apd-2, in three of the treated groups. The expression level of the gene loc102656552, which is related to hypertrehalosemic hormone, was significantly downregulated. Hypertrehalosemic hormone can promote fat and glycogen decomposition in the body and increase the concentrations of lipids and trehalose in the blood (Rockstein, 1998). Trehalase converts trehalose stored in insects into glucose which is metabolized through glycolysis or the pentose phosphate pathway (Becker et al., 1996). The downregulated expression of loc102656552 could affect the synthesis of trehalose in honeybees, resulting a reduced trehalose content and affecting the growth, development, and resproduction of honeybees. Chen (2021) found that both carbendazim and Nosema ceranae could lead to the upregulated expression of genes encoding trehalose transporters and trehalase in honeybees, reducing blood trehalose levels. Cytochrome oxidase (Cox) plays an important role in the oxidative phosphorylation process of cells (Barazzoni et al., 2000). The expression of Cox6c was significantly downregulated after honeybee larvae received flumethrin, indicating that it could affect the process of oxidative phosphorylation, even at very low concentrations. The oxidative phosphorylation pathway is considered to be the most important biochemical process in animal cells. It mainly occurs in the inner membrane of mitochondria. As the final metabolic pathway of the respiratory chain, oxidative phosphorylation can provide adenosine triphosphate for a variety of basic cellular pathways, such as neural activity, material synthesis, and bioelectrogenesis (Byrne, 2009). The expression levels of ribosomal protein-related genes (rpl39, loc725432, and loc100578551) were significantly downregulated with flumethrin exposure. Ribosomal protein is an important part of the ribosome that plays an essential role in protein synthesis and translation (Byrne, 2009). Downregulated expression of these genes suggests that flumethrin exposure may affect energy metabolism and inhibit protein synthesis of honeybees. A recent study showed that sublethal doses of thiamethoxam caused the downregulation of genes related to oxidative phosphorylation and ribosomes in honeybees (Shi, 2020). The apidermin (APD) protein family is a newly discovered family of structural epidermal proteins in insects. Apd-2 belongs to the APD protein gene family and controls the main components of exoskeleton proteins in honeybees (Sun et al., 2012). Therefore, the downregulated expression of this epidermal gene may be a morphological manifestation of developmental delay. A previous study found that the expression levels of some epidermal protein family genes in the brain of honeybees exposed to carbendazim were downregulated (Fan, 2020). The present study showed that in all flumethrin-treated groups, genes involved in metabolism and biochemical processes in honeybee heads were significantly downregulated.

Annotation of enriched GO terms revealed that genes associated with the oxidation-reduction process and ion transport were significantly downregulated with exposure to flumethrin, which could lead to the generation of reactive oxidative species (ROS) (Figure 4). Increased oxidative stress was observed in neonicotinoid-exposed honeybees due to the excessive production of ROS (James and Xu, 2012). Iron plays an important role in redox reactions, the downregulation of genes involved in iron ion binding may indicate altered iron homeostasis in the brains of flumethrin-treated honeybees. A sublethal dose of imidacloprid has been shown to lead to downregulated expression levels of genes associated with the oxidation-reduction process and ion transport (Li et al., 2019). KEGG pathway analysis showed that most of the altered pathways were related to biological metabolic processes and biochemical reactions of the body (Figure 5). Together, these results show that flumethrin concentrations above 0.01 mg/L can affect the expression levels of genes related to biochemical processes and metabolism of substances in the heads of honeybees, affecting the growth and development of honeybees.

With the increasing flumethrin concentrations, the expression levels of olfactory-related genes, including CSP2, CSP3, OBP17, and OBP3, decreased significantly in the 1 and 0.1 mg/L groups compared to controls (Table S2). OBP17 and OBP3 belong to the odorant binding protein family, which are a class of water-soluble proteins that can bind odor molecules and help insects identify odors of a small molecular mass (Mu and Dong, 2004). CSP2 and CSP3 belong to the family of chemosensory proteins, whose main function is identifying, binding, and transporting non-volatile molecules (Xie et al., 2016). Imidacloprid can significantly reduce the expression of olfactory-related genes in the honeybee brain (Li et al., 2019). The down-regulation of olfactory-related genes may indicate an impaired olfactory function in honeybees exposed to flumethrin, leading to dysfunctional behaviors. The honeybee’s sense of smell is closely related to all of its life activities. For example, worker bees sense queen pheromones through smell, which helps to stabilize the whole colony; the drone senses the queen by smell and copulates with her (Xie et al., 2016). The changes in olfactory-related gene expression levels observed here may affect the recognition of informative materials and the exchange of information between honeybees, thus affecting the function of the entire colony. The antioxidant gene SOD1 was significantly downregulated in the 1 and 0.1 mg/L groups compared to controls. SOD1 can remove excess ROS in insects and protect the body from environmental stress (Mccord and Fridovich, 1969).

The higher the concentration of flumethrin in the treatment group, the higher the number of DEGs observed. Some DEGs, such as immune and detoxification-related genes (GST, loc406081) and antioxidant gene (MsrA), were differentially expressed only in the 1 mg/L group (Table S3). These three genes had significantly downregulated expression in the 1 mg/L group. GSTs are a multifunctional super gene family of enzymes that can metabolize various endogenous and exogenous substances and participate in the body’s detoxification of exogenous substances (e.g., pesticides). In the insect body, loc406081 can regulate the oxidation of glucose, produce energy and oxidation reactions to promote detoxification functions, and produce H_2_0_2_, which has an antibacterial function (Bucekova et al., 2014). Methionine sulfoxide reductase A (MsrA) belongs to the methylthionine sulfoxide reductase family, which are antioxidant and protein-repair factors in organisms with indirect antioxidant effects (Gong et al., 2012). Compared with the 0.01 and 0.1 mg/L groups, 1 mg/L flumethrin had a considerable impact on the expression levels of immunity, detoxification, and antioxidant genes of honeybees and a negative impact on the survival of honeybees.

## 5 Conclusion

This study showed that the residues of flumethrin at honey-relevant levels could affect the physiology of honeybee larvae and newly emerged worker honeybees (*A. mellifera*) exposed to flumethrin during the larval stage, and that with increases residual concentrations, the impact would be greater. Therefore, in beekeeping, we should pay close attention to the residues of flumethrin in bee products to ensure the health of honeybees and the production of high-quality bee products. In addition, the mechanisms through which honeybees can repair this damage after becoming adults remains to be further studied.

## Data Availability

The datasets presented in this study can be found in online repositories. The names of the repository/repositories and accession number(s) can be found below: https://www.ncbi.nlm.nih.gov/,PRJNA866287.
