# Supplementary material for: Effects of larval exposure to the insecticide flumethrin on the development of honeybee (Apis mellifera) workers
Source: Front Physiol. 2022 Dec 14;13:1054769. doi: 10.3389/fphys.2022.1054769 (PMC9795074; doi:10.3389/fphys.2022.1054769)
Supplement: Supplementary file 1 [file Table1.DOCX]

Supplemental Table S1. Summary of statistics for transcriptome in newly emerged honeybees exposed to flumethrin.

| Sample | Raw reads | Clean reads | Clean bases | Q20 | Q30 |
| --- | --- | --- | --- | --- | --- |
| GA1 | 44746538 | 43984024 | 6.60G | 96.86 | 91.66 |
| GA2 | 40824732 | 40259938 | 6.04G | 96.88 | 91.67 |
| GA3 | 47849312 | 47066346 | 7.06G | 97.06 | 92.00 |
| GB1 | 47245458 | 46563334 | 6.98G | 97.18 | 92.19 |
| GB2 | 44395416 | 43808234 | 6.57G | 97.24 | 92.45 |
| GB3 | 46658876 | 45976058 | 6.90G | 97.41 | 92.75 |
| GC2 | 40337738 | 39815956 | 5.97G | 97.36 | 92.58 |
| GC3 | 46730696 | 46031120 | 6.90G | 97.24 | 92.43 |
| GD1 | 40737228 | 40021190 | 6.00G | 97.12 | 92.24 |
| GD2 | 44752748 | 43967094 | 6.60G | 97.05 | 92.11 |
| GD3 | 44856618 | 44013574 | 6.60G | 97.57 | 93.12 |

Notice: GA, GB, GC and GD represent 1mg/L,0.1mg/L,0.01mg/L and 0mg/L group respectively．
