# Supplementary material for: Effects of larval exposure to the insecticide flumethrin on the development of honeybee (Apis mellifera) workers
Source: Front Physiol. 2022 Dec 14;13:1054769. doi: 10.3389/fphys.2022.1054769 (PMC9795074; doi:10.3389/fphys.2022.1054769)
Supplement: Supplementary file 2 [file Table2.DOCX]

Supplemental Table S2. The RPKM values from RNA-Seq of the DEGs common in the 1 and 0.1 mg/L groups.

| gene_name | 1mg/L | 0.1mg/L | 0.01mg/L | 0mg/L |
| --- | --- | --- | --- | --- |
| LOC409678 | 1.887571382 | 1.224310394 | 0.932275561 | 0.751703421 |
| LOC724639 | 42.07051811 | 49.7831971 | 68.94507439 | 75.52844959 |
| LOC100577163 | 76.39795842 | 57.3420796 | 139.966006 | 139.221127 |
| LOC410550 | 7.956614065 | 7.81224791 | 12.30483999 | 13.46417077 |
| LOC101664701 | 29.12711423 | 31.8428582 | 38.72352036 | 46.46815966 |
| - | 9.160936658 | 8.139279822 | 11.4832216 | 14.57296913 |
| ACSF2 | 192.1320987 | 182.2673865 | 332.6054648 | 365.7550919 |
| LOC725147 | 125.8933546 | 165.3905606 | 220.3908764 | 304.5552089 |
| LOC113218630 | 137.748346 | 155.5029312 | 186.4191877 | 216.5751275 |
| LOC724631 | 50.58748186 | 69.00347928 | 89.27569014 | 107.5382398 |
| LOC409346 | 7.669013465 | 7.308138712 | 5.263297139 | 4.672199412 |
| LOC408516 | 278.0227736 | 310.8403784 | 407.3867271 | 417.3058936 |
| LOC409616 | 18.69689883 | 17.44821916 | 13.67781035 | 13.35275927 |
| LOC551479 | 138.0846433 | 169.5880279 | 252.7445851 | 292.8094782 |
| LOC724264 | 120.246548 | 148.3822372 | 185.1073376 | 199.1293661 |
| Rpl41 | 427.8953693 | 534.0676747 | 837.5339335 | 1029.601999 |
| LOC107965288 | 99.76743896 | 108.6410619 | 131.4876022 | 160.6960941 |
| Tom7 | 110.6139381 | 134.9700633 | 177.0612056 | 185.0449788 |
| LOC408961 | 53.52650514 | 71.40362539 | 101.0996163 | 100.8368044 |
| LOC408285 | 17.66177615 | 16.19305508 | 10.8012877 | 11.73856565 |
| LOC726747 | 139.8915233 | 154.4429965 | 204.2956417 | 238.8344201 |
| LOC100578213 | 5.403478674 | 5.355181293 | 9.786552536 | 10.0456603 |
| LOC410465 | 21.58187668 | 22.82529094 | 33.7762105 | 33.93169243 |
| LOC726118 | 287.3066519 | 295.3733695 | 367.8520554 | 462.0981993 |
| CSP2 | 290.5762612 | 318.996394 | 420.9171055 | 418.017228 |
| LOC410824 | 47.56342325 | 55.41923576 | 85.73820119 | 81.76570655 |
| - | 3.350200787 | 2.889588009 | 6.832657845 | 6.315257048 |
| CSP3 | 5708.91647 | 5735.152308 | 7990.210299 | 8634.302912 |
| LOC725383 | 824.0260951 | 974.6919457 | 1153.883969 | 1448.096825 |
| LOC551318 | 175.4228133 | 191.3245908 | 253.3759517 | 259.3252147 |
| LOC107964134 | 17.23954531 | 16.0275181 | 31.8893746 | 32.84771112 |
| Obp17 | 151.3205063 | 146.1622256 | 202.5863707 | 232.2104397 |
| LOC410117 | 38.3642495 | 38.71314287 | 27.54760871 | 27.09472238 |
| LOC411677 | 87.6834604 | 90.90553955 | 122.4887382 | 131.7159508 |
| LOC726247 | 0.792004493 | 0.990256788 | 1.824690521 | 2.065370273 |
| LOC724489 | 13.40195973 | 16.06916648 | 21.74717771 | 24.61101139 |
| LOC410517 | 23.45192133 | 23.52790653 | 42.08472323 | 40.22408802 |
| LOC408335 | 28.76312501 | 27.9534162 | 20.8619252 | 21.39983399 |
| LOC410167 | 51.35443418 | 41.79483499 | 70.20941352 | 90.02392015 |
| LOC724221 | 308.9303179 | 299.5029696 | 382.1617494 | 476.9932681 |
| LOC552724 | 36.81768014 | 38.84925574 | 53.29634023 | 59.30109397 |
| LOC409699 | 46.11988288 | 49.60841842 | 67.87045726 | 64.53738674 |
| LOC408696 | 272.9278975 | 309.0055159 | 359.2799413 | 407.9898626 |
| LOC725380 | 284.631098 | 364.1450677 | 546.4466049 | 674.853964 |
| LOC409090 | 64.36561356 | 67.3613967 | 86.644311 | 95.23963356 |
| LOC100576814 | 24.34850205 | 21.98505591 | 34.85119799 | 39.06060021 |
| Ndufb2 | 107.6219215 | 110.0052661 | 133.1486577 | 164.1522188 |
| LOC725230 | 29.67559868 | 27.49286678 | 44.42374974 | 47.514558 |
| LOC408427 | 27.34618798 | 27.19081055 | 18.99860616 | 18.82327237 |
| LOC724378 | 12.88312241 | 13.79643903 | 21.69739793 | 21.21183393 |
| LOC725038 | 665.4852352 | 698.1860798 | 958.6068801 | 990.7280952 |
| LOC408411 | 4.919900663 | 4.672761866 | 5.483482584 | 6.917144781 |
| - | 0.764429676 | 0.903399177 | 1.557339476 | 2.119394564 |
| LOC408807 | 12.37533799 | 412.989136 | 21.17970026 | 29.31445519 |
| Y-f | 120.5721485 | 133.6993581 | 148.1143724 | 172.7788256 |
| - | 98.0027379 | 99.47690438 | 126.6556229 | 144.1456277 |
| LOC100578262 | 5.897256444 | 5.834603019 | 4.167501983 | 4.367936928 |
| Y-y | 73.18158236 | 75.95813311 | 97.26144519 | 113.7802188 |
| LOC411582 | 13.08067492 | 12.73525644 | 10.43261666 | 9.3183223 |
| - | 60.69124185 | 62.3421376 | 77.8986357 | 92.47989829 |
| LOC725414 | 6.712627834 | 8.941695052 | 14.311601 | 14.21626282 |
| LOC100578782 | 153.0773288 | 160.0852409 | 188.2796708 | 227.9264227 |
| LOC410365 | 6.836988961 | 7.653773444 | 8.00986612 | 10.20818267 |
| LOC107965367 | 734.8376636 | 681.332207 | 895.8019294 | 1321.068387 |
| LOC100577363 | 6.173871255 | 5.056242324 | 10.05382039 | 13.75981574 |
| LOC551541 | 483.1258002 | 478.131523 | 599.8351447 | 683.2773558 |
| LOC727483 | 126.8154362 | 143.4243456 | 174.3012405 | 191.5438701 |
| LOC725547 | 125.8713502 | 126.8745486 | 164.307119 | 194.5925359 |
| LOC725776 | 2.536769753 | 2.876894471 | 3.759519387 | 4.026193161 |
| LOC408633 | 14.3644754 | 15.05795941 | 23.40316785 | 20.73169207 |
| LOC551250 | 10.46040764 | 10.12224527 | 17.36495085 | 15.97137942 |
| LOC726841 | 12.20258425 | 11.35521169 | 8.963486599 | 8.680217493 |
| LOC411854 | 34.36178771 | 32.43627353 | 26.82218342 | 25.74426785 |
| Cox6b1 | 155.8768153 | 158.6055587 | 198.7323968 | 218.4469608 |
| LOC726863 | 9.323500041 | 9.101377879 | 12.26875901 | 16.16664023 |
| Ef-1a-f1 | 136.7821051 | 135.7548352 | 161.7555692 | 197.9649127 |
| LOC726757 | 46.88827287 | 49.97402662 | 63.94473019 | 63.83710308 |
| LOC100576132 | 13.36110186 | 12.52431405 | 9.027252775 | 9.582756033 |
| LOC724638 | 19.26221505 | 22.32540839 | 36.03596049 | 34.61243577 |
| LOC409177 | 4.713428673 | 4.090818926 | 2.751080486 | 3.084964118 |
| LOC113218623 | 16.30974121 | 16.19884282 | 19.02512491 | 25.11807412 |
| LOC100577456 | 42.33166379 | 40.13407724 | 66.29035691 | 68.14478343 |
| LOC551381 | 469.5014233 | 497.3806283 | 610.9885818 | 675.098281 |
| LOC107964319 | 2.626761765 | 2.716422007 | 3.134896216 | 3.680824684 |
| LOC724919 | 40.66170981 | 42.32669544 | 58.46604564 | 64.88181532 |
| LOC552216 | 3.644397352 | 3.347435358 | 2.258807328 | 2.211120219 |
| LOC410434 | 7.437804899 | 7.687667507 | 11.86662747 | 11.02282458 |
| LOC725283 | 14.58566511 | 11.53230322 | 16.94413828 | 22.25286669 |
| LOC726297 | 421.7539343 | 411.1713648 | 499.8113524 | 637.0354568 |
| LOC727165 | 284.2353415 | 321.4027783 | 381.0073207 | 481.4240748 |
| LOC726259 | 32.9924598 | 32.52387385 | 45.59244439 | 47.81325825 |
| LOC411539 | 10.27536721 | 9.787906646 | 8.059695269 | 7.871701283 |
| LOC726321 | 206.46015 | 208.3008071 | 244.2174949 | 281.4524837 |
| LOC413047 | 6.207541683 | 6.906236559 | 8.283570922 | 9.212296592 |
| LOC727026 | 31.84378061 | 31.62644119 | 40.00330766 | 45.90084535 |
| LOC552610 | 448.7813772 | 461.7840829 | 577.0905249 | 615.6351028 |
| LOC409847 | 16.90336423 | 16.7610087 | 13.52577431 | 12.28713896 |
| LOC412742 | 6.889561394 | 6.41043237 | 4.722504832 | 5.184547335 |
| LOC410271 | 38.63029149 | 40.13649596 | 53.53423548 | 53.65631132 |
| LOC410837 | 266.2597053 | 257.094081 | 381.0162443 | 370.4267472 |
| LOC409497 | 11.57081613 | 11.13553639 | 8.969211508 | 8.759535709 |
| LOC410256 | 1.920437662 | 1.846349384 | 1.071378135 | 1.284659921 |
| LOC413501 | 0.906401355 | 0.811236783 | 1.147105966 | 1.667833306 |
| LOC100576967 | 16.6694984 | 17.21119138 | 26.3827571 | 24.62361423 |
| LOC113219052 | 0.037673239 | 0.194282354 | 0.239652932 | 0.634083365 |
| LOC409500 | 47.50940618 | 49.59216891 | 65.82867578 | 64.32131551 |
| LOC408424 | 48.83407614 | 52.20806911 | 65.99575509 | 69.75252572 |
| LOC409187 | 163.7671273 | 159.4252773 | 255.7701414 | 245.4181877 |
| LOC411159 | 9.60740545 | 10.18945216 | 13.61692938 | 14.69598499 |
| LOC100578818 | 2.268574722 | 2.293229568 | 3.922993251 | 3.732807704 |
| LOC726958 | 20.55220037 | 20.95677078 | 26.24660026 | 29.16563711 |
| LOC724421 | 46.41735186 | 48.42201746 | 77.66152344 | 81.60451926 |
| LOC726887 | 15.83511957 | 15.45207351 | 13.32896098 | 11.03816361 |
| - | 41.74263866 | 43.86933158 | 49.07035301 | 59.19443022 |
| LOC724211 | 89.06844722 | 93.09157426 | 158.4904262 | 142.1127836 |
| LOC410788 | 7.626825843 | 7.934090974 | 10.05386708 | 10.52600496 |
| LOC100577168 | 5.412132522 | 5.881850247 | 7.691537457 | 7.677576615 |
| LOC551947 | 3.162716729 | 3.074342823 | 2.311319561 | 2.316782363 |
| LOC409757 | 9.674745053 | 9.940202696 | 6.964668377 | 7.342079337 |
| NLG-3 | 3.463806754 | 3.361472125 | 3.914547965 | 4.674094168 |
| Wat | 107.6489025 | 82.81761143 | 137.0838764 | 143.0097382 |
| LOC552523 | 8.208679206 | 6.682155568 | 13.15090681 | 16.9242981 |
| LOC725980 | 44.20509645 | 42.53552421 | 56.44517774 | 61.76905776 |
| LOC551974 | 60.00647609 | 63.74503538 | 83.86057143 | 79.70759585 |
| LOC408955 | 43.27786545 | 44.27347936 | 54.82708715 | 55.39703759 |
| LOC551072 | 31.80467544 | 30.34578917 | 42.5472338 | 40.90293807 |
| LOC409271 | 6.549412302 | 6.555148137 | 4.441587255 | 5.145038562 |
| LOC100577777 | 3.413341514 | 4.146116005 | 5.752383317 | 6.749424617 |
| LOC726302 | 1.958254639 | 1.893515783 | 3.495437234 | 3.263857648 |
| - | 11.06011857 | 10.98015531 | 13.743861 | 15.44482649 |
| LOC727049 | 64.35199901 | 69.46093849 | 78.98855913 | 93.13856868 |
| LOC551668 | 8.992060261 | 9.327153031 | 7.6235259 | 6.931731259 |
| LOC409788 | 21.82938892 | 23.82313681 | 16.76065407 | 16.74418248 |
| LOC412544 | 4.846130247 | 6.269059681 | 3.725592417 | 3.877791751 |
| LOC552276 | 44.57024423 | 47.45991705 | 62.14934759 | 65.07615894 |
| LOC410009 | 0.216689191 | 0.221926724 | 0.339157368 | 0.344957791 |
| LOC410915 | 11.61621912 | 27.67639466 | 23.86430825 | 18.11891848 |
| LOC552741 | 56.87089865 | 56.25615374 | 74.39203858 | 74.06617488 |
| - | 1.207957782 | 1.354348141 | 0.483782318 | 0.296817397 |
| LOC100576839 | 0.33061753 | 0.371532107 | 0.574702993 | 0.667888071 |
| LOC409717 | 15.06876232 | 15.95336407 | 11.86518796 | 12.08692478 |
| LOC726611 | 17.65919327 | 51.36042888 | 31.97563148 | 26.18839978 |
| LOC725947 | 17.99559215 | 16.08381793 | 23.46481724 | 25.44075577 |
| LOC107965181 | 0.610014759 | 0.555836308 | 0.428723396 | 0.323114396 |
| LOC410688 | 14.03635631 | 13.93893074 | 11.46170717 | 11.29656862 |
| LOC552528 | 49.11681806 | 55.97855545 | 43.21644276 | 39.58679812 |
| LOC102654839 | 0.57682447 | 0.576341026 | 1.207645934 | 0.985042403 |
| Sod1 | 644.9087057 | 634.851258 | 933.0010365 | 862.3337657 |
| LOC551997 | 5.393247204 | 5.209294807 | 4.291276001 | 3.956862112 |
| LOC100578921 | 20.60439647 | 21.14574663 | 29.04328959 | 28.59191719 |
| LOC412825 | 6.88441207 | 7.550382469 | 10.57709983 | 10.49026355 |
| LOC410254 | 3.940571448 | 3.323661687 | 4.820793463 | 6.137311923 |
| LOC100577381 | 13.34309023 | 13.37013117 | 19.28508327 | 20.58136779 |
| NFRKB | 8.936878665 | 9.11257999 | 6.423956951 | 7.113624408 |
| LOC100576901 | 2.81703988 | 3.447521367 | 5.645954436 | 5.687791991 |
| Tsf1 | 224.8113649 | 651.0422986 | 342.4625147 | 349.4066353 |
| LOC413289 | 1.264295196 | 1.368336904 | 2.076792989 | 1.881622609 |
| LOC726336 | 12.26927724 | 12.07902989 | 9.7611298 | 9.518494505 |
| LOC725797 | 79.42233774 | 79.5098867 | 94.97038001 | 113.0481877 |
| LOC408365 | 86.28935648 | 91.65561724 | 135.0156715 | 138.5114127 |
| LOC102654920 | 9.831962883 | 10.53443288 | 10.7638039 | 14.95856773 |
| LOC725705 | 35.01924331 | 35.00286193 | 57.83965543 | 49.93637641 |
| Cpap3-b | 134.8662751 | 116.2967079 | 174.1450099 | 187.3618253 |
| LOC413549 | 10.91189457 | 11.19277429 | 15.18333816 | 15.06279249 |
| LOC726842 | 2.257642213 | 1.95292632 | 2.925528539 | 3.275232252 |
| LOC413789 | 8.246929652 | 19.93428584 | 18.48456088 | 12.61600933 |
| LOC100577883 | 5.94338366 | 26.52695869 | 17.31994926 | 10.74378531 |
| LOC409702 | 4.158160279 | 4.500373329 | 3.290900713 | 3.113529214 |
| LOC724829 | 26.0567757 | 26.95529811 | 42.43424141 | 36.89365849 |
| LOC412127 | 15.81443892 | 16.50407509 | 25.56931706 | 22.50852001 |
| LOC726918 | 3.099695159 | 3.14912084 | 1.90638024 | 2.313149868 |
| LOC726552 | 54.05525247 | 51.89810036 | 69.12097805 | 69.55340051 |
| LOC100578232 | 26.9833247 | 23.40215801 | 39.08894312 | 36.03702011 |
| LOC100578243 | 1.556344133 | 1.734204412 | 5.638174408 | 4.23279992 |
| LOC411326 | 28.85973282 | 29.27812769 | 22.67441142 | 23.2033602 |
| LOC727236 | 4.418490844 | 4.469242559 | 3.302942404 | 3.441289392 |
| LOC409563 | 12.64538876 | 12.58012763 | 10.2808512 | 9.741147821 |
| Fibroin4 | 1.599678551 | 1.72569622 | 2.640276177 | 2.950689386 |
| LOC413612 | 41.6881464 | 42.7254416 | 58.18467803 | 55.06192139 |
| LOC725596 | 44.88112699 | 46.64335473 | 35.01732495 | 35.6861784 |
| LOC727028 | 8.808126984 | 7.927703707 | 14.6351559 | 14.65703915 |
| LOC551323 | 95.41675662 | 94.80917536 | 136.1352683 | 145.8146292 |
| LOC408409 | 42.29920623 | 43.39153507 | 35.09355992 | 34.61355996 |
| LOC552694 | 6.690246282 | 6.623110082 | 4.015265337 | 5.022039981 |
| LOC102653587 | 0.430042448 | 0.378032123 | 0.352742038 | 0.110451745 |
| LOC100576163 | 10.66109872 | 9.066532932 | 16.86802304 | 17.58698689 |
| LOC725068 | 10.05641183 | 10.19593139 | 7.715441451 | 8.189114119 |
| LOC725532 | 0.384002123 | 0.438356056 | 0.492954651 | 0.827020363 |
| LOC411019 | 2.573157185 | 2.508257572 | 3.369088165 | 3.635976013 |
| LOC410151 | 12.42314721 | 12.41486981 | 8.390271249 | 10.02234776 |
| LOC411068 | 8.481391361 | 8.900899956 | 6.957599144 | 6.914279909 |
| LOC551424 | 9.173695791 | 9.294503614 | 6.634845623 | 7.274834349 |
| LOC100577081 | 13.41954653 | 13.6047098 | 16.129325 | 17.57162981 |
| LOC725748 | 3.892297483 | 3.782189282 | 5.870762028 | 5.72124203 |
| LOC552073 | 10.82916259 | 10.78057711 | 13.37674277 | 14.31356727 |
| LOC408264 | 5.287301511 | 5.275130917 | 7.358424752 | 8.182805987 |
| LOC727254 | 9.277665406 | 8.248833938 | 10.80705992 | 12.57772714 |
| LOC102656725 | 10.46979213 | 10.69968364 | 13.21677609 | 15.43557865 |
| LOC551984 | 8.553851564 | 9.726166505 | 7.296849347 | 6.822932123 |
| LOC100577064 | 1.377444525 | 1.397414876 | 0.883807036 | 0.600806473 |
| LOC725344 | 6.92269539 | 6.57327971 | 10.05145595 | 11.04886187 |
| - | 7.353732137 | 7.844352877 | 14.25512887 | 11.2891371 |
| LOC552558 | 0.151962338 | 0.145180609 | 0.245509452 | 0.381350189 |
| LOC727129 | 4.326801331 | 3.378500072 | 5.86276598 | 6.570907814 |
| LOC412166 | 19.70914157 | 60.06541956 | 49.63490199 | 32.90137992 |
| LOC102654715 | 26.79817673 | 29.96269278 | 21.38007995 | 20.8545559 |
| LOC413567 | 35.28130878 | 31.24582723 | 45.12290415 | 45.73799017 |
| LOC100577783 | 16.88039378 | 16.32314225 | 23.1469145 | 24.69629662 |
| LOC551185 | 10.28627543 | 10.75010053 | 8.226282604 | 8.332700681 |
| LOC411219 | 8.034783847 | 7.680267957 | 5.476761036 | 5.663955522 |
| LOC411723 | 16.07491347 | 17.25992708 | 12.66141706 | 12.85088026 |
| LOC726793 | 59.51905052 | 55.11112059 | 64.00380693 | 81.19817603 |
| LOC409902 | 5.229296393 | 5.343949544 | 3.744985187 | 4.069197289 |
| LOC726277 | 15.6179627 | 14.10040197 | 18.1738253 | 21.38445685 |
| LOC726664 | 13.84210989 | 16.21314279 | 10.69388119 | 11.15912541 |
| LOC412133 | 4.222751855 | 4.601591594 | 3.163270186 | 3.2316279 |
| LOC551397 | 45.81231532 | 47.23634519 | 56.98753577 | 59.63431413 |
| LOC408872 | 14.70662772 | 11.28236056 | 18.37106996 | 20.21427566 |
| LOC410420 | 29.01617006 | 28.67484568 | 38.19355552 | 37.96034469 |
| LOC409417 | 117.0720916 | 121.1709091 | 98.96726989 | 99.02322996 |
| LOC410005 | 2.316912941 | 2.326953244 | 2.625610088 | 3.214171753 |
| LOC409278 | 213.4928964 | 206.6989093 | 262.9837536 | 258.4627062 |
| LOC726497 | 8.675313277 | 9.455556479 | 6.801563867 | 7.224908769 |
| LOC102656425 | 19.44692747 | 19.55726856 | 31.82287744 | 27.49238786 |
| LOC552190 | 113.0835098 | 103.0573691 | 176.4444603 | 160.3950123 |
| LOC107964436 | 23.26706313 | 23.43875454 | 30.02283652 | 34.2294071 |
| LOC409697 | 17.16328958 | 16.33378393 | 21.36914717 | 24.67217858 |
| LOC551089 | 0.771451358 | 0.843679752 | 0.295404493 | 0.344878274 |
| sGC-alpha1 | 12.36405002 | 11.50238102 | 13.33762189 | 15.93207879 |
| LOC552211 | 61.25587605 | 52.70373797 | 69.29437492 | 76.38179901 |
| LOC727247 | 8.801656973 | 8.729818502 | 11.5312229 | 11.66167785 |
| LOC408579 | 7.100008622 | 6.933144319 | 8.478046469 | 9.134853648 |
| LOC411953 | 7.275919295 | 6.165224392 | 9.527176985 | 9.783516417 |
| LOC725170 | 29.18277807 | 27.90656596 | 33.69153057 | 37.74680498 |
| LOC102656088 | 1.953951596 | 1.749603628 | 2.313874487 | 2.869102531 |
| LOC410811 | 9.54069136 | 9.795889645 | 8.074993551 | 8.015025914 |
| LOC727170 | 6.560828826 | 6.771418067 | 4.833290568 | 4.779373441 |
| LOC413152 | 2.358406137 | 2.670793008 | 2.148385001 | 1.793677956 |
| LOC409619 | 0.204995706 | 0.18237136 | 0.380560036 | 0.388156642 |
| LOC408548 | 65.08719777 | 72.12058412 | 53.06393267 | 54.05773666 |
| LOC102654090 | 15.5092986 | 14.85318049 | 17.00876447 | 20.03666496 |
| LOC724187 | 5.408496989 | 4.15497505 | 8.627858002 | 8.529775175 |
| LOC727599 | 87.99640248 | 87.9942894 | 106.5331566 | 113.235375 |
| LOC113218812 | 113.3326496 | 107.2058611 | 131.9883692 | 145.9265657 |
| Obp3 | 32.62009168 | 20.98088368 | 64.72037904 | 53.65793862 |
| LOC410554 | 6.037592112 | 5.980112394 | 7.694108882 | 7.937735673 |
| LOC411378 | 217.6839051 | 178.2180872 | 265.5025809 | 281.5604558 |
| LOC413669 | 5.640675029 | 7.091222978 | 5.143995754 | 4.598984504 |
| LOC411961 | 11.3895442 | 11.98017904 | 8.670006516 | 9.328545026 |
| LOC724647 | 8.600070827 | 8.364133466 | 10.70617672 | 11.09717493 |
| LOC100577377 | 4.463447056 | 4.202312037 | 5.03467388 | 5.78200508 |
| LOC409663 | 208.9392431 | 190.3232899 | 267.4249882 | 277.2220432 |
| LOC726914 | 3.877108591 | 3.753692965 | 4.590978168 | 5.23766667 |
| LOC726106 | 3.338450601 | 3.03754835 | 3.530867064 | 4.38012201 |
| LOC411488 | 11.54821981 | 12.17569086 | 11.61473271 | 8.711447244 |
| LOC100577150 | 1.087647182 | 0.88996696 | 1.621798948 | 1.852597645 |
| LOC408669 | 24.35280565 | 22.98730466 | 26.10021952 | 32.4297286 |
| LOC100577192 | 65.32416105 | 57.08921442 | 76.21119837 | 81.97619291 |
| LOC100576467 | 59.00650915 | 57.77519735 | 80.78979677 | 72.87783073 |
| LOC102655446 | 0.818944529 | 1.114366453 | 0.768398966 | 0.510966588 |
| LOC102654510 | 3.057094479 | 4.015110931 | 2.06426453 | 2.258654116 |
| LOC411750 | 10.43069692 | 11.18201523 | 8.699072323 | 8.89008115 |
| LOC413858 | 12.20465829 | 14.65946046 | 8.426250503 | 9.312342358 |
| LOC102655054 | 1.237036386 | 1.179661618 | 1.578267439 | 2.013121032 |
| LOC727300 | 28.75583704 | 27.25709326 | 35.72788984 | 36.58571133 |
| LOC100576540 | 0.61668489 | 0.614236409 | 0.933081873 | 1.208421113 |
| LOC551924 | 21.46851813 | 23.25576044 | 16.70567976 | 16.53479819 |
| LOC410779 | 7.606126102 | 7.569948414 | 8.230834821 | 9.816502578 |
| LOC552573 | 20.74787517 | 21.60448845 | 15.50054513 | 17.08222447 |
| LOC408532 | 357.6204178 | 395.0643652 | 285.144977 | 275.1372185 |
| LOC408787 | 36.14311401 | 35.88434203 | 42.86625869 | 46.25872262 |
| LOC113219378 | 0.176874822 | 0.159700257 | 0.29633295 | 0.319999218 |
| LOC552625 | 4.479312178 | 4.922701354 | 4.347862376 | 3.578880064 |
| LOC724865 | 11.2844171 | 36.19372913 | 28.43814846 | 17.86206344 |
| LOC409531 | 15.81863949 | 25.70411471 | 23.05029139 | 19.91375268 |
| LOC412924 | 46.73167417 | 49.84701929 | 35.19614965 | 40.1425694 |
| LOC412077 | 9.638358823 | 9.966899386 | 8.543372258 | 8.053496571 |
| LOC100577527 | 9.418916802 | 9.196599973 | 12.98097112 | 13.45615058 |
| LOC408878 | 0.10611972 | 0.158180015 | 0.063032289 | 0.038460845 |
| LOC725420 | 8.727435243 | 8.081972565 | 12.28036995 | 11.56758415 |
| LOC726381 | 3.912618702 | 4.05311787 | 2.618105334 | 3.13175134 |
| LOC411060 | 2.102162019 | 2.277978321 | 1.763194311 | 1.704555914 |
| LOC726210 | 52.04770614 | 46.85375527 | 59.91552112 | 64.67113911 |
| - | 1.717685613 | 2.052141376 | 1.47851581 | 1.120497446 |
| LOC551219 | 2.580887616 | 2.715980742 | 2.139927334 | 1.829669326 |
| LOC551519 | 54.68540303 | 56.98994826 | 41.92452068 | 46.66732364 |
| LOC410994 | 7.414761327 | 6.809805721 | 7.978579247 | 10.66591715 |
| LOC107965237 | 4.303960574 | 4.382014613 | 5.740307317 | 6.205686836 |
| LOC726541 | 6.713840299 | 7.531333503 | 5.507595256 | 5.286133566 |
| LOC725616 | 13.20083283 | 11.51716352 | 13.84311417 | 18.43270234 |
| LOC413185 | 2.724109136 | 2.897444394 | 2.395725255 | 2.059765822 |
| LOC100578368 | 5.224762531 | 4.654238775 | 5.272969683 | 6.366089891 |
| LOC552320 | 1.703114806 | 1.812116493 | 2.737245497 | 2.698337966 |
| aub | 7.864718802 | 7.684755282 | 4.973148756 | 5.156394062 |
| Uqcr11 | 440.6995279 | 414.8346452 | 438.9803612 | 561.0682879 |
| LOC409255 | 5.196711239 | 6.286540586 | 4.816818463 | 4.20420955 |
| LOC552513 | 1.904372139 | 1.871826195 | 2.076757564 | 2.821375915 |
| LOC726711 | 20.26293233 | 18.91525432 | 28.053008 | 25.74198268 |
| COX2 | 3493.185761 | 3330.273864 | 3500.442146 | 4756.71934 |
| LOC113219396 | 3.355493564 | 3.678725502 | 2.987423922 | 2.787293622 |
| Pgrp-s2 | 21.93320104 | 399.4438108 | 120.8494298 | 53.32598059 |
| LOC726733 | 181.9718599 | 149.8944329 | 202.2620883 | 237.817197 |
| LOC724293 | 10.90396806 | 29.27340274 | 18.99493136 | 15.60945686 |
| - | 394.5563585 | 342.2405125 | 411.7824655 | 525.2105041 |
| LOC552473 | 5.3434003 | 5.929200106 | 4.538696622 | 4.249745196 |
| - | 5.886657829 | 6.058543549 | 8.146521384 | 8.570537549 |
| LOC100576290 | 0.291267506 | 0.260383517 | 0.275545611 | 0.53213799 |
| LOC726201 | 8.575212965 | 8.923194622 | 5.504869988 | 5.90030654 |
| LOC408497 | 7.278810669 | 8.007634359 | 7.222056514 | 5.862973832 |
| LOC551514 | 2.325027162 | 2.109308743 | 3.347651796 | 3.027472727 |
| LOC725128 | 50.5051294 | 47.80663183 | 88.82759332 | 69.28415555 |
| LOC410655 | 0.740837717 | 1.936301215 | 1.58499974 | 1.214831608 |
| LOC410083 | 5.193344772 | 5.432600628 | 4.032312719 | 4.34233476 |
| LOC100578680 | 9.051984457 | 10.0484028 | 7.141187901 | 7.093312679 |
| LOC100577901 | 132.4156881 | 104.2353152 | 154.059881 | 183.8660914 |
| LOC409881 | 2164.492958 | 2067.910053 | 2979.690464 | 2824.868139 |
| LOC724561 | 2.324999993 | 2.214877417 | 2.674367398 | 3.203957153 |
| - | 15.49752156 | 11.99542522 | 16.75117436 | 20.30423298 |
| LOC411729 | 2.081999718 | 5.535691936 | 4.759910061 | 3.131757123 |
| LOC725942 | 74.76708666 | 68.48182516 | 84.09002699 | 90.11559372 |
| Y-e3 | 0.332562521 | 3.269977927 | 0.582289323 | 0.631810691 |
| Calpb | 28.96514 | 29.90368923 | 26.38152425 | 24.66582305 |
| TRNAA-GGC | 0.467005434 | 0.199285866 | 1.185488509 | 2.737343141 |
| LOC408681 | 4.715612913 | 4.244240759 | 5.295613102 | 6.483126375 |
| LOC726833 | 21.01232647 | 23.02665541 | 17.60795682 | 17.63592908 |
| LOC100577573 | 0.290726469 | 0.278280009 | 0.693955261 | 0.805563375 |
