# Supplementary material for: Effects of larval exposure to the insecticide flumethrin on the development of honeybee (Apis mellifera) workers
Source: Front Physiol. 2022 Dec 14;13:1054769. doi: 10.3389/fphys.2022.1054769 (PMC9795074; doi:10.3389/fphys.2022.1054769)
Supplement: Supplementary file 3 [file Table3.DOCX]

Supplemental Table S3. The RPKM values from RNA-Seq of the DEGs specific in the 1 mg/L group.

| gene_name | 1mg/L | 0.1mg/L | 0.01mg/L | 0mg/L |
| --- | --- | --- | --- | --- |
| Obp13 | 25.56185931 | 43.35930112 | 63.17224705 | 53.806703 |
| LOC724471 | 42.97186358 | 33.97072136 | 25.91438802 | 26.04412075 |
| LOC409025 | 21.68382535 | 36.39236942 | 56.18286076 | 43.39993628 |
| LOC725701 | 10.68764964 | 15.38246633 | 15.79379025 | 17.46228402 |
| - | 4.461335435 | 1.933186919 | 1.397418658 | 1.600997487 |
| LOC725524 | 30.81286745 | 23.58934956 | 18.37646518 | 20.44986314 |
| LOC551223 | 124.0824051 | 80.89191022 | 80.25898044 | 61.74898155 |
| LOC100576118 | 22.48770402 | 28.45126881 | 39.30725594 | 36.57246717 |
| LOC413332 | 4.605110236 | 3.494647303 | 2.4882191 | 2.677820576 |
| LOC410860 | 5.88987233 | 9.787223644 | 10.2538325 | 9.775547584 |
| LOC413036 | 2.286721384 | 1.562101577 | 1.358789038 | 1.450634381 |
| LOC412701 | 5.227462596 | 3.858542769 | 3.212902444 | 3.403612019 |
| LOC727185 | 6.78819972 | 4.822784849 | 3.906568722 | 4.49485908 |
| LOC725258 | 20.96997849 | 39.21343726 | 49.43338213 | 36.32028319 |
| LOC411535 | 17.42721177 | 11.70317967 | 10.53451113 | 10.61494921 |
| LOC551723 | 25.33066062 | 39.72806704 | 60.37647807 | 60.51650176 |
| LOC100577763 | 3.459714064 | 1.88042216 | 1.700342422 | 1.890545539 |
| LOC102653899 | 91.26097513 | 44.4531531 | 46.29048294 | 47.91213061 |
| LOC726866 | 13.21462157 | 17.11216772 | 19.82529755 | 19.77232693 |
| LOC409949 | 25.49117251 | 33.38957682 | 38.18208601 | 40.47483842 |
| LOC408864 | 16.22538942 | 42.94650795 | 30.0811365 | 27.96039286 |
| LOC412224 | 8.67143311 | 6.898748815 | 5.349612163 | 5.899742601 |
| LOC412176 | 3.271815742 | 2.164168562 | 1.778738542 | 1.970536834 |
| - | 33.73670097 | 66.01374243 | 90.5128527 | 68.07807804 |
| LOC551782 | 19.86547152 | 6.213483793 | 5.16033771 | 6.474008278 |
| LOC409228 | 58.92932044 | 76.53083073 | 96.59715897 | 89.7091475 |
| LOC552370 | 4.526180368 | 3.710479464 | 2.861172791 | 2.985178844 |
| LOC409953 | 18.62004172 | 11.17682974 | 9.098353542 | 11.38079614 |
| LOC409906 | 11.10945369 | 8.11105305 | 5.992811035 | 6.813196793 |
| LOC408388 | 504.3102011 | 622.2800873 | 776.8066263 | 712.2745642 |
| LOC410161 | 9.98295748 | 7.349540895 | 4.921857797 | 5.895464524 |
| LOC100578382 | 0.976730711 | 0.480577319 | 0.400937432 | 0.397416271 |
| LOC412104 | 22.39078668 | 19.05899327 | 15.10660847 | 15.17828785 |
| LOC409043 | 8.775851274 | 11.13976592 | 13.58916713 | 13.54767111 |
| LOC724625 | 25.74878588 | 47.40159717 | 62.04096504 | 49.65830798 |
| LOC408702 | 24.52481149 | 28.93200828 | 36.05767456 | 36.15198238 |
| - | 6.060499345 | 4.072275762 | 2.947230381 | 3.747197776 |
| LOC726728 | 6.909551194 | 5.013761751 | 3.926886297 | 4.352525287 |
| LOC413749 | 47.79919544 | 126.7406505 | 103.8779237 | 85.03520154 |
| LOC552545 | 11.41129985 | 13.84767596 | 16.66288982 | 17.81225079 |
| LOC724216 | 5.106343975 | 8.144280497 | 10.39197857 | 10.16876318 |
| LOC725098 | 13.03691106 | 9.07931204 | 7.487216071 | 8.018220918 |
| LOC724560 | 5.095515257 | 2.941126341 | 2.742525122 | 2.914608645 |
| LOC551280 | 11.33633023 | 21.43133368 | 22.7999857 | 20.93825116 |
| LOC413091 | 11.86725245 | 9.122920136 | 8.015444806 | 8.022004587 |
| LOC552309 | 12.19981121 | 9.41431141 | 7.125135542 | 7.837741307 |
| LOC413331 | 6.360622302 | 5.552955104 | 4.605490154 | 4.474044141 |
| LOC410019 | 3.692708171 | 2.512989028 | 1.881109802 | 2.321989748 |
| LOC551678 | 81.89668916 | 95.8351231 | 133.5693702 | 120.6787959 |
| LOC552712 | 61.18523139 | 80.10016605 | 104.0306231 | 93.09942826 |
| LOC408729 | 10.39700576 | 13.64581994 | 15.46554938 | 16.27510556 |
| LOC724594 | 2.408948873 | 4.96021681 | 6.240931514 | 5.115453047 |
| LOC725074 | 328.2528261 | 376.9218705 | 444.9643815 | 513.4270386 |
| LOC412256 | 5.78029845 | 4.557613826 | 3.659099401 | 3.88600137 |
| Gat-a | 13.59599318 | 21.1984657 | 20.87580789 | 18.97200903 |
| LOC102656585 | 53.67090613 | 90.60646403 | 91.99987137 | 90.71269177 |
| LOC551651 | 573.9282013 | 699.6125889 | 849.0755465 | 782.8774873 |
| LOC725681 | 4.167869828 | 2.200465764 | 1.647876394 | 2.215499831 |
| LOC409045 | 16.35691961 | 11.53428433 | 9.015737171 | 10.66264981 |
| LOC726322 | 26.6078248 | 36.67350002 | 39.47402337 | 39.29502675 |
| LOC408705 | 21.7938025 | 17.04441065 | 12.36880386 | 14.49636893 |
| LOC408552 | 72.19156842 | 106.4253448 | 155.2812793 | 143.1156779 |
| LOC413223 | 8.870187732 | 7.018544765 | 7.001985603 | 6.354776445 |
| LOC552461 | 43.73787033 | 94.8938294 | 70.11437844 | 68.03684487 |
| LOC409252 | 3.684096702 | 2.53694913 | 1.722807683 | 2.156713866 |
| LOC411207 | 9.113809447 | 21.98628019 | 14.91319001 | 14.83170647 |
| LOC552278 | 13.17550368 | 15.91309905 | 19.16619693 | 19.15166793 |
| LOC113218950 | 21.26655723 | 25.65404296 | 34.06624937 | 33.92809409 |
| LOC726726 | 8.363900238 | 5.758241305 | 4.761724687 | 5.247014867 |
| LOC724169 | 3.409853641 | 8.24142553 | 8.378566424 | 6.938324157 |
| LOC725253 | 130.6257102 | 161.0533572 | 213.6601706 | 214.7101048 |
| LOC410516 | 60.09737611 | 79.16793872 | 100.4201955 | 83.27581687 |
| LOC726238 | 45.99429785 | 38.44790142 | 29.36260569 | 33.25332074 |
| LOC409288 | 10.00775099 | 17.91907257 | 15.26318143 | 14.99277839 |
| LOC100577113 | 6.424757845 | 4.912023092 | 3.956940759 | 4.123762414 |
| LOC408308 | 1253.423948 | 1450.463192 | 1890.337615 | 1821.408442 |
| LOC551361 | 10.07104236 | 7.740328471 | 5.427835706 | 6.678363217 |
| LOC725503 | 6.742027261 | 4.636410425 | 3.272893833 | 4.182295129 |
| LOC408774 | 10.371791 | 8.043510718 | 7.181727048 | 7.43150102 |
| LOC409861 | 338.326228 | 485.1536551 | 685.7958346 | 572.6020891 |
| LOC412746 | 2.693277079 | 2.043692951 | 1.552789091 | 1.907536871 |
| LOC724402 | 5.447151454 | 3.976734281 | 3.142245014 | 3.539798261 |
| LOC726908 | 7.97356242 | 4.993859475 | 3.433236584 | 4.2707218 |
| LOC552677 | 7.701044815 | 5.318071905 | 4.729824062 | 4.939621076 |
| LOC552502 | 6.230017431 | 5.047653811 | 4.387613682 | 4.264022018 |
| LOC552323 | 41.06644474 | 53.43347438 | 61.85661049 | 65.44401423 |
| LOC408533 | 5.142414517 | 4.55638486 | 3.976388799 | 3.558300473 |
| LOC726855 | 0.129631404 | 0.333244187 | 0.520822454 | 0.694761944 |
| LOC408277 | 19.79694062 | 23.43911967 | 35.74781599 | 34.69110223 |
| LOC100576895 | 2.681219296 | 3.599053448 | 5.902409588 | 4.392814129 |
| ROCK2 | 7.607464299 | 6.387438022 | 4.805483894 | 5.465405568 |
| LOC413054 | 3.672500945 | 7.126809479 | 5.062644416 | 5.801002783 |
| LOC725179 | 19.89428314 | 14.30093593 | 12.15682056 | 12.59516457 |
| LOC725883 | 71.11843605 | 118.0748347 | 165.4699998 | 136.3135422 |
| - | 1.533307295 | 2.956782477 | 4.087694954 | 3.469682137 |
| LOC411756 | 4.440897312 | 3.142782531 | 2.644198573 | 2.895119856 |
| LOC726672 | 5.817665908 | 16.39930546 | 11.75852296 | 14.92485068 |
| LOC100576829 | 7.363011054 | 4.463350012 | 3.906399834 | 4.402401008 |
| LOC726653 | 21.3933974 | 24.86407962 | 31.38237844 | 32.06816724 |
| LOC552215 | 14.88039494 | 20.79843328 | 23.3549393 | 24.47125955 |
| LOC100577214 | 11.30909099 | 14.32847317 | 16.64407192 | 15.9705605 |
| LOC551566 | 2.16973865 | 1.587025884 | 1.262465511 | 1.318993544 |
| LOC726679 | 9.521976101 | 6.284180481 | 5.072378148 | 6.652828702 |
| LOC552548 | 3.960983507 | 1.936529943 | 1.760566195 | 2.205474802 |
| LOC552357 | 0.814435792 | 0.427870158 | 0.340548461 | 0.318440849 |
| LOC726414 | 7.174327038 | 11.81337886 | 14.36854958 | 13.32369871 |
| LOC551555 | 20.59892244 | 16.27063812 | 14.54803697 | 15.56327468 |
| LOC107963991 | 14.01583911 | 11.11800032 | 9.661695093 | 9.44266314 |
| LOC552530 | 3.674050213 | 2.256820338 | 2.19817534 | 2.29907017 |
| LOC408937 | 688.8457608 | 914.2179325 | 1159.504538 | 1038.368256 |
| LOC552283 | 239.0319824 | 335.6798594 | 416.9833428 | 364.9569704 |
| LOC724744 | 102.3418738 | 128.1265525 | 160.4550741 | 174.0330939 |
| LOC551572 | 14.70313432 | 25.68117647 | 23.46717617 | 20.92041932 |
| LOC411806 | 6.113725812 | 5.129452301 | 4.434620856 | 4.296390648 |
| LOC408551 | 84.78359117 | 165.1556085 | 232.3622359 | 181.3285368 |
| LOC552508 | 1.075711479 | 0.432899925 | 0.455074699 | 0.438750709 |
| LOC410884 | 0.676301614 | 2.112804822 | 2.089745289 | 1.885465673 |
| LOC725651 | 5.102264422 | 5.822054152 | 6.792429389 | 8.8377298 |
| LOC409493 | 7.014253364 | 5.717715461 | 3.832324563 | 4.472988162 |
| LOC411175 | 6.312298151 | 8.613625902 | 9.884200308 | 9.168666967 |
| LOC552731 | 8.081934337 | 6.035933296 | 4.556266646 | 5.163721214 |
| LOC100578928 | 1.778343968 | 0.9844259 | 0.894817856 | 0.815980879 |
| LOC411904 | 9.12907281 | 7.84574658 | 6.051402932 | 6.676527724 |
| LOC113218621 | 7.167258905 | 5.027682008 | 4.333735616 | 4.307862367 |
| - | 3.250313664 | 1.659581251 | 1.805380282 | 1.959810733 |
| Hex70c | 0.567011863 | 1.122738263 | 1.23167135 | 1.380390398 |
| LOC551803 | 0.698871511 | 1.08829882 | 1.103425803 | 1.21585703 |
| LOC726860 | 204.1767689 | 227.1000949 | 293.679161 | 276.991781 |
| LOC102655512 | 0.573863351 | 1.017858457 | 1.453791562 | 1.114276218 |
| LOC100577972 | 3.934281709 | 2.210052212 | 1.546803388 | 2.103040772 |
| LOC552149 | 15.58939521 | 45.78975231 | 35.51382527 | 29.73335756 |
| LOC725273 | 11.75568898 | 17.45058728 | 25.89944124 | 21.22421032 |
| LOC100578484 | 1.154210411 | 2.109060126 | 2.241180488 | 2.422415598 |
| LOC726608 | 6.287148935 | 5.364021007 | 4.690607764 | 4.567048033 |
| LOC725406 | 13.18358155 | 10.02420697 | 8.351716238 | 8.666446714 |
| LOC102656442 | 17.91267653 | 20.90040571 | 23.9326536 | 26.67147914 |
| LOC726997 | 7.573382334 | 16.40133765 | 13.35266649 | 12.95548733 |
| LOC100576563 | 19.80746679 | 22.91853137 | 27.29398761 | 27.91122544 |
| LOC552235 | 8.424076002 | 6.757133988 | 5.122101177 | 5.266227022 |
| LOC725882 | 6.578840289 | 9.71054388 | 11.94231233 | 11.35084227 |
| Fabp | 98.45292736 | 135.7585308 | 147.8324084 | 166.331043 |
| LOC726941 | 11.13278813 | 9.675768658 | 7.270859589 | 8.338354491 |
| LOC551765 | 36.47591703 | 60.61589117 | 73.32789987 | 62.7311871 |
| LOC726126 | 35.57764515 | 50.22410288 | 60.6951717 | 64.37130019 |
| LOC409740 | 8.625835729 | 15.95834965 | 22.59637542 | 15.19175205 |
| LOC409249 | 5.36295102 | 3.133413247 | 2.537026579 | 3.189790868 |
| LOC725641 | 19.4224072 | 22.6115177 | 32.93139876 | 28.37303449 |
| LOC408664 | 2.957635364 | 3.651280637 | 4.520648504 | 4.417649483 |
| LOC408329 | 56.62415234 | 87.20832021 | 124.7341202 | 102.5837982 |
| LOC727278 | 6.892747652 | 5.507526773 | 4.321319706 | 4.970334659 |
| LOC726949 | 15.49539528 | 12.78761232 | 8.457734019 | 10.69926867 |
| LOC725309 | 37.90177302 | 40.94733381 | 67.07397821 | 69.9955148 |
| Hbg2 | 0.223401655 | 1.745982106 | 0.627158974 | 0.853676129 |
| LOC100578813 | 1.207609046 | 2.559889177 | 3.182750088 | 2.885144492 |
| LOC409153 | 3.302324399 | 2.572747727 | 2.195331243 | 2.204603333 |
| LOC412332 | 16.76566579 | 13.33999517 | 10.61634236 | 11.75551663 |
| LOC411173 | 6.992285988 | 8.996306716 | 10.92970019 | 10.71456289 |
| LOC724740 | 3.154285914 | 3.869746481 | 4.668658263 | 5.167782065 |
| LOC411758 | 66.39652267 | 46.94088786 | 35.12068912 | 44.46111576 |
| LOC552101 | 17.01599966 | 21.04982312 | 22.82659282 | 25.93223315 |
| LOC100578201 | 2.804288756 | 1.996665264 | 1.756695072 | 1.828303234 |
| LOC408782 | 424.3412496 | 456.8036757 | 634.5983335 | 622.3444827 |
| LOC552468 | 9.424069428 | 5.698454801 | 4.706720594 | 5.468508246 |
| LOC725784 | 13.13418209 | 17.24003661 | 17.99127641 | 19.50926988 |
| LOC100577886 | 2.872147609 | 1.241272446 | 1.141269329 | 1.537955532 |
| LOC409325 | 11.5695947 | 6.994779502 | 6.59643434 | 7.249720134 |
| LOC100577587 | 16.1504191 | 13.22146738 | 9.23491976 | 10.34467189 |
| LOC725291 | 47.59548395 | 51.81657911 | 63.96803417 | 66.75577399 |
| LOC100578450 | 4.550970975 | 3.062819504 | 2.530198765 | 2.67085538 |
| LOC411609 | 8.657885844 | 10.80541541 | 15.57202231 | 14.57304643 |
| LOC551165 | 12.86672609 | 22.65571348 | 22.01495814 | 18.94975115 |
| LOC410070 | 1.791152017 | 0.932165507 | 0.772519814 | 0.959595506 |
| LOC409634 | 125.4042017 | 106.7851954 | 77.635747 | 89.16493036 |
| LOC552142 | 1.076120032 | 1.654097549 | 1.521175446 | 1.727007678 |
| LOC411791 | 16.99855015 | 13.83113036 | 10.5597907 | 12.28249229 |
| LOC408455 | 1.037826071 | 1.812675993 | 1.81319493 | 1.844690627 |
| LOC551150 | 14.2394084 | 11.105665 | 8.095899983 | 9.653280723 |
| LOC552773 | 22.12127039 | 25.50271912 | 32.0507495 | 33.47289052 |
| LOC552494 | 153.4876009 | 193.2246698 | 264.4010598 | 274.0796149 |
| LOC410718 | 6.537362393 | 8.006662197 | 10.74578877 | 10.10717078 |
| LOC413753 | 2.696383942 | 2.305754094 | 1.930132894 | 1.943544552 |
| LOC552481 | 7.425879955 | 8.379990504 | 11.43316005 | 10.92060386 |
| LOC552257 | 9.903078091 | 15.06564442 | 15.56864728 | 15.73075864 |
| LOC102654949 | 27.95711132 | 24.26483584 | 17.61585178 | 19.03443304 |
| - | 2.645882538 | 3.967617637 | 4.678203478 | 4.516083569 |
| LOC550981 | 39.23196703 | 44.59372562 | 60.77934759 | 55.5462843 |
| RpLP1 | 1016.063612 | 1236.054522 | 1604.173932 | 1562.164682 |
| LOC412643 | 6.485729484 | 3.895001806 | 2.815475848 | 3.69854635 |
| LOC725950 | 9.495603728 | 6.780713341 | 6.039749194 | 6.26529098 |
| LOC411555 | 12.96044716 | 11.27405109 | 8.74255534 | 9.340736924 |
| LOC726002 | 9.766226071 | 7.649471787 | 6.214321786 | 6.828610751 |
| PGRP-S3 | 24.73467718 | 31.3518255 | 43.59870693 | 39.49658433 |
| LOC726625 | 203.4368481 | 105.0587252 | 121.1704434 | 119.6201092 |
| LOC408759 | 4.66088792 | 5.853545604 | 6.308314344 | 7.044552321 |
| LOC409736 | 18.17740673 | 23.62389489 | 30.35660661 | 28.29806787 |
| LOC413099 | 5.754970971 | 4.275522289 | 3.348673206 | 3.995756549 |
| LOC552044 | 19.04062366 | 16.46489765 | 12.84097385 | 14.0458185 |
| LOC413984 | 9.035536481 | 6.168816163 | 4.792875364 | 5.526629212 |
| LOC725572 | 95.8143439 | 104.4986009 | 126.7076 | 130.0877053 |
| LOC551895 | 7.183449005 | 10.45645224 | 12.69415092 | 11.59537356 |
| LOC412081 | 4.942546853 | 4.323681985 | 3.696552226 | 3.669513122 |
| LOC409143 | 37.93305813 | 49.8284017 | 88.74983887 | 75.87569068 |
| LOC409282 | 13.9321673 | 9.84713966 | 7.55221378 | 9.413882599 |
| LOC725724 | 14.76514962 | 11.14076811 | 10.59333689 | 10.6381976 |
| LOC725266 | 53.41062307 | 64.81110245 | 87.8190835 | 79.19383793 |
| LOC552527 | 8.059071942 | 7.124661437 | 5.351314388 | 5.937114429 |
| TyHyd | 109.5928111 | 207.2422913 | 150.6361907 | 177.3509849 |
| LOC412157 | 6.239286592 | 5.583280928 | 4.670377611 | 4.418449427 |
| LOC552177 | 8.583911749 | 5.858323111 | 4.360352684 | 5.788521044 |
| LOC726766 | 13.45891022 | 10.17211301 | 8.661944364 | 9.165023435 |
| LOC409125 | 2.29265146 | 1.869490413 | 1.355378563 | 1.420721829 |
| LOC726746 | 35.20545319 | 44.79631723 | 44.64459375 | 52.94332283 |
| LOC551331 | 35.45778128 | 25.5257899 | 19.8517409 | 24.13475747 |
| LOC410982 | 17.05121954 | 21.37220883 | 31.56662417 | 26.70601018 |
| LOC409961 | 11.73567468 | 18.96871427 | 27.323508 | 20.59448736 |
| LOC726647 | 20.38687056 | 13.25984322 | 11.60744022 | 13.62675606 |
| LOC409549 | 175.5953244 | 211.3609018 | 269.7812103 | 240.2011995 |
| LOC411520 | 104.6968924 | 128.8678958 | 157.3072502 | 148.3147335 |
| LOC413689 | 9.518796555 | 8.153382855 | 7.225775561 | 6.79528833 |
| LOC551530 | 6.91304792 | 5.523421242 | 4.454692432 | 4.924391088 |
| LOC102654012 | 6.881433125 | 8.536885748 | 10.16873666 | 10.55415688 |
| LOC100578514 | 0.243066969 | 0.5803198 | 1.013988372 | 1.309428159 |
| - | 3.239820151 | 4.667155523 | 4.357031247 | 7.257519486 |
| LOC100578433 | 4.004516383 | 3.119345878 | 2.31956823 | 2.812805274 |
| LOC551686 | 12.94060177 | 9.37352626 | 8.973486643 | 9.198349825 |
| LOC724747 | 6.860932819 | 5.652819416 | 4.595661357 | 5.146245499 |
| LOC412468 | 16.01589451 | 13.88738379 | 13.1285937 | 12.60564741 |
| LOC100578257 | 10.19616481 | 6.099839567 | 5.2316025 | 5.808624794 |
| LOC551411 | 108.3435325 | 125.0553889 | 149.8524375 | 145.6978202 |
| rtGEF | 10.31090504 | 8.286864596 | 6.571699746 | 7.356312176 |
| LOC552658 | 8.041499367 | 6.526841737 | 5.892410784 | 6.06341935 |
| LOC100577903 | 5.354841001 | 2.421651783 | 1.671384177 | 2.571778093 |
| LOC726222 | 451.2873663 | 500.2796601 | 605.9049267 | 630.7883216 |
| LOC552792 | 1.987957958 | 1.210966915 | 1.74288311 | 1.284187939 |
| LOC726272 | 6.93700954 | 4.429972694 | 3.600818477 | 4.178365457 |
| LOC409921 | 8.320346237 | 10.65174448 | 11.76855481 | 11.48632584 |
| LOC100577918 | 0.81476133 | 1.455432375 | 2.958961356 | 1.917737728 |
| LOC725155 | 15.68060749 | 11.61956953 | 9.989710404 | 11.04606442 |
| arm | 28.94172987 | 37.46923636 | 48.80587154 | 46.93452559 |
| LOC409273 | 15.80572913 | 14.71024049 | 11.51910137 | 12.51828057 |
| LOC409789 | 29.68787967 | 38.85636121 | 49.53059163 | 45.47990776 |
| LOC100576330 | 13.69582844 | 21.38552606 | 24.21470905 | 21.64583863 |
| - | 4.231658057 | 2.198645611 | 1.849307121 | 2.154521935 |
| LOC410121 | 5.004498783 | 4.23336098 | 4.210814657 | 3.670807801 |
| LOC100577401 | 6.081894978 | 4.12249385 | 2.912808594 | 3.562359854 |
| LOC408850 | 21.86973789 | 18.55940322 | 15.15149152 | 16.9083139 |
| LOC726378 | 9.16108964 | 13.68522606 | 15.46244307 | 14.84166699 |
| Dnmt1a | 1.276582471 | 0.493386525 | 0.35885034 | 0.574140544 |
| LOC726438 | 3.432139536 | 1.953696396 | 1.634094843 | 1.997705338 |
| LOC410148 | 15.9828563 | 21.8711576 | 22.69823918 | 26.16490219 |
| LOC409930 | 474.1790286 | 513.6651703 | 620.721715 | 653.5535367 |
| LOC726350 | 5.310026773 | 10.00895621 | 14.23614564 | 10.09530193 |
| LOC408694 | 18.5783615 | 25.65492685 | 31.90191895 | 27.79408306 |
| LOC409203 | 16.18361425 | 25.37552354 | 24.2048131 | 23.41449914 |
| LOC725434 | 2.904581598 | 3.942190882 | 4.696501758 | 4.592489541 |
| UHRF1 | 1.871059937 | 1.546535883 | 1.140311936 | 1.146307095 |
| LOC725707 | 3.731217078 | 2.088949659 | 1.753092822 | 2.04071757 |
| LOC552061 | 5.931815542 | 3.740713674 | 2.97273804 | 3.476226696 |
| LOC411570 | 38.51710018 | 47.38756267 | 54.24878789 | 53.95571768 |
| LOC727128 | 500.3529673 | 661.5326589 | 802.7616119 | 798.8191457 |
| LOC102655243 | 12.15205862 | 26.15121137 | 37.94176149 | 25.6588615 |
| LOC408570 | 5.005607753 | 13.639976 | 10.42343114 | 10.09266771 |
| LOC408324 | 48.53876085 | 68.90355312 | 77.52294855 | 71.76203242 |
| LOC551061 | 11.52943172 | 10.01675582 | 7.416452595 | 8.579724998 |
| LOC413460 | 8.519969764 | 8.021574927 | 6.08519746 | 6.683068894 |
| LOC725248 | 7.434094398 | 5.535273438 | 4.950621638 | 4.813696274 |
| LOC726241 | 12.64781718 | 10.5624454 | 8.014005759 | 9.087786361 |
| LOC107964845 | 2.031572948 | 3.908766857 | 4.448517739 | 3.98897584 |
| LOC726582 | 2.73041953 | 3.950724197 | 4.769258172 | 4.532236014 |
| LOC410468 | 9.529609447 | 18.24405903 | 15.7656471 | 14.10029178 |
| LOC726178 | 38.06954156 | 34.22324751 | 30.38090795 | 30.80391894 |
| LOC408367 | 249.3484833 | 315.70337 | 384.7575791 | 355.8948384 |
| LOC100576402 | 2.903995909 | 1.738740664 | 1.492487462 | 1.820422195 |
| LOC100578865 | 2.03156005 | 2.41050905 | 2.973631651 | 3.026030908 |
| LOC409993 | 6.662429432 | 5.466524888 | 4.249288069 | 4.814671003 |
| LOC727127 | 4.251191092 | 2.937693 | 3.029836274 | 2.800491176 |
| LOC410795 | 8.469408574 | 12.83114763 | 14.08280476 | 14.1106074 |
| LOC409536 | 59.12068449 | 39.47631612 | 38.70624499 | 44.82268919 |
| LOC410563 | 2.585612854 | 3.184260105 | 3.993649197 | 4.442599412 |
| LOC113219383 | 5.734367526 | 7.402637123 | 6.775491033 | 8.364423619 |
| LOC725315 | 95.02357411 | 111.2807804 | 133.2828637 | 127.8315756 |
| LOC410322 | 8.727245386 | 18.72640476 | 11.69740147 | 12.12970404 |
| LOC410368 | 16.63577059 | 22.3923852 | 28.89708771 | 27.16134729 |
| LOC551804 | 1.892067181 | 3.005711117 | 3.134289151 | 3.165116916 |
| LOC552642 | 8.492307764 | 5.958709695 | 5.392083745 | 5.226549364 |
| LOC408699 | 34.94618196 | 21.79580692 | 17.21093485 | 21.07956681 |
| LOC408898 | 3.31659913 | 4.867492981 | 6.440543445 | 5.697598293 |
| - | 1.401966288 | 0.782668908 | 0.652324181 | 0.794758818 |
| LOC551710 | 62.10767185 | 71.64962089 | 99.70752164 | 83.61799509 |
| LOC724333 | 6.072400747 | 6.844901947 | 7.935090695 | 8.722716995 |
| LOC412837 | 3.12689611 | 3.892017761 | 5.088753317 | 4.609938483 |
| LOC726221 | 95.83329484 | 119.5926603 | 142.4969399 | 139.2364503 |
| LOC410092 | 7.591863926 | 8.74144435 | 11.30433022 | 10.64711 |
| LOC726826 | 17.16012542 | 13.33331677 | 11.74403287 | 11.61481756 |
| LOC725662 | 2.437369733 | 1.905034404 | 1.401894795 | 1.670350504 |
| LOC100577155 | 0.64175934 | 0.929398285 | 1.129323536 | 1.440399786 |
| LOC409316 | 14.01361152 | 12.74660737 | 10.15685019 | 10.4084334 |
| LOC100578618 | 3.903980738 | 7.713000803 | 7.170714814 | 7.345424431 |
| LOC107966086 | 0.988599678 | 1.858935453 | 1.750027992 | 1.884982542 |
| LOC410301 | 2.423605332 | 1.346267578 | 1.333744394 | 1.540294912 |
| LOC552462 | 21.17546094 | 24.34350984 | 33.79587055 | 30.98435214 |
| LOC102654448 | 1.072490884 | 1.252594009 | 1.265955544 | 1.581463774 |
| LOC551449 | 6.18577085 | 4.60150021 | 3.54339335 | 4.231247216 |
| LOC727407 | 4.899522101 | 3.855163268 | 3.494000757 | 3.393190494 |
| LOC410782 | 13.94408573 | 10.9894357 | 8.315128416 | 9.768314758 |
| LOC725528 | 29.95872853 | 21.60998289 | 17.57237221 | 20.50705054 |
| LOC552425 | 0.70128955 | 1.007576379 | 1.524233869 | 1.39923869 |
| LOC726204 | 6.071021244 | 7.693724739 | 10.46836975 | 10.30083957 |
| LOC100578434 | 8.925790062 | 6.280684936 | 4.89451811 | 6.413212814 |
| LOC726101 | 11.13399642 | 16.9274048 | 19.81943672 | 19.80231555 |
| LOC412130 | 16.34560257 | 14.32028135 | 11.39651516 | 12.35085008 |
| LOC411906 | 2.240235869 | 1.596017124 | 1.385420866 | 1.482630222 |
| LOC725088 | 4.349236777 | 2.876704339 | 2.499726459 | 2.956844758 |
| LOC551033 | 3.325332402 | 2.384254351 | 2.660471041 | 2.353749798 |
| LOC100578339 | 3.688797818 | 2.496338878 | 2.153858388 | 2.408099637 |
| LOC552531 | 126.7666803 | 142.0706849 | 215.2105695 | 172.5517292 |
| LOC410136 | 14.56962821 | 10.98561854 | 8.956813267 | 10.85486328 |
| LOC552377 | 13.15509795 | 21.91265455 | 25.60324526 | 24.62666168 |
| LOC410217 | 6.324489599 | 3.50063777 | 3.426824317 | 3.859355824 |
| LOC413423 | 6.237613942 | 8.99341277 | 11.89616824 | 10.34882911 |
| LOC408583 | 153.1011815 | 169.4934588 | 228.5095001 | 220.6655062 |
| LOC411515 | 390.5840234 | 547.0100311 | 650.2173924 | 642.9188966 |
| LOC102656283 | 9.63974097 | 6.523147166 | 4.410930564 | 5.783458517 |
| LOC411577 | 13.76525549 | 11.17663059 | 9.725245925 | 8.396975689 |
| LOC414009 | 2.670628589 | 1.962473144 | 1.410670575 | 1.641584348 |
| LOC724765 | 1.795554186 | 2.926995956 | 3.425968757 | 2.817261304 |
| LOC408649 | 4.322257137 | 7.741928406 | 10.33128402 | 7.661052523 |
| LOC102654083 | 6.126656457 | 5.308423599 | 4.593726321 | 4.350318992 |
| LOC410239 | 5.465905395 | 4.742186535 | 3.754976795 | 3.959055355 |
| LOC408509 | 31.12286318 | 40.95266156 | 46.83592883 | 42.02760716 |
| LOC552277 | 103.4215194 | 113.5500029 | 129.5142801 | 162.6271186 |
| LOC107964762 | 1.544360601 | 3.884168501 | 4.811983779 | 3.164817329 |
| LOC414017 | 4.541224299 | 2.16054206 | 2.257993248 | 2.464501886 |
| LOC725070 | 25.39391785 | 21.41774185 | 17.52801856 | 19.09352893 |
| LOC725668 | 1.810565838 | 1.227832076 | 0.965955307 | 0.961926152 |
| LOC726230 | 21.06109268 | 46.33383914 | 46.65144516 | 37.25759688 |
| LOC409243 | 3.706645834 | 1.586425332 | 1.281897946 | 1.936380125 |
| LOC552007 | 285.6563119 | 415.9225957 | 500.7213866 | 415.9896355 |
| LOC113218918 | 0.623598846 | 0.264722717 | 0.209225453 | 0.214916091 |
| LOC100578976 | 2.421740776 | 3.419056501 | 2.985663002 | 3.374361243 |
| LOC409217 | 7.447034233 | 6.2314392 | 4.98938936 | 5.446591849 |
| LOC409350 | 32.84069272 | 23.34396689 | 19.74394126 | 22.96597208 |
| LOC411014 | 98.24872104 | 139.0932295 | 179.7736856 | 152.6949385 |
| LOC412195 | 6.787020629 | 5.057477172 | 5.391704057 | 4.908629136 |
| LOC724367 | 30.45349954 | 52.69955263 | 50.76625491 | 57.5375081 |
| LOC410940 | 3.966439248 | 2.411042424 | 2.169533328 | 2.504374489 |
| LOC413787 | 12.3229512 | 10.5153187 | 8.55200508 | 9.023273308 |
| LOC107965449 | 31.08827303 | 26.50571294 | 23.88760322 | 22.00197228 |
| LOC552765 | 4.589812727 | 3.485366467 | 2.906675652 | 3.003737661 |
| LOC102656661 | 1.248378519 | 1.427363359 | 2.242963424 | 1.828613313 |
| LOC552210 | 21.25418708 | 23.72037172 | 35.29546969 | 29.76612391 |
| LOC413799 | 24.08615159 | 28.48954793 | 39.37573657 | 35.92912054 |
| Ant | 1803.31096 | 2489.375585 | 3033.863062 | 2532.195974 |
| LOC413408 | 3.779876297 | 7.796243428 | 7.299399085 | 6.039019273 |
| LOC726894 | 25.52842429 | 30.71113399 | 39.04783679 | 40.86066564 |
| LOC724915 | 1.969055075 | 1.669342062 | 1.184700114 | 1.195902496 |
| LOC410097 | 5.449077312 | 4.319405644 | 2.811546896 | 3.395454173 |
| LOC413125 | 0.199455678 | 0.784498224 | 0.706680328 | 0.500684385 |
| LOC100578515 | 9.370852156 | 18.10645343 | 17.91984165 | 15.63895062 |
| LOC726103 | 8.599697784 | 6.638065286 | 5.929348463 | 5.989662992 |
| LOC411238 | 2.389979863 | 5.922787401 | 7.333810811 | 5.33987157 |
| LOC413833 | 3.610808056 | 12.53984553 | 12.0800458 | 7.944795828 |
| LOC725065 | 2.644840844 | 1.66654418 | 1.7053422 | 1.612109994 |
| LOC409053 | 6.201185163 | 5.096392484 | 3.749898447 | 4.272543334 |
| LOC409492 | 23.47603793 | 19.91691735 | 16.66533063 | 17.41776209 |
| - | 2.210817482 | 1.422217575 | 1.412259488 | 1.109626981 |
| LOC411249 | 2.342772848 | 1.932257876 | 1.94717832 | 1.539687326 |
| LOC726904 | 5.006928365 | 4.026622774 | 3.6806958 | 3.643878909 |
| LOC413517 | 351.5221632 | 451.3565344 | 570.8546913 | 490.5399533 |
| LOC413369 | 6.410493348 | 3.823998225 | 4.042678977 | 3.9133824 |
| LOC413515 | 6.854841474 | 6.006608667 | 5.276143234 | 4.868268042 |
| LOC408909 | 134.3333651 | 159.7212794 | 195.4563783 | 188.0277679 |
| LOC409802 | 94.7870938 | 101.7138881 | 129.901802 | 123.1938083 |
| LOC726564 | 6.298860505 | 7.025103775 | 9.343272189 | 8.998737229 |
| LOC413457 | 6.848174843 | 5.226283278 | 4.240177935 | 4.570214241 |
| LOC410850 | 1.210240771 | 0.613844987 | 0.428892353 | 0.620603754 |
| LOC724468 | 0.479526045 | 0.751471176 | 0.59254958 | 0.913831186 |
| Ndufs5 | 333.195167 | 362.9504236 | 429.8267119 | 449.9635541 |
| LOC410236 | 5.650402863 | 9.414566803 | 9.986206398 | 8.603653667 |
| LOC725393 | 1.042906192 | 2.792534011 | 3.446649404 | 2.160804148 |
| LOC727246 | 30.19459925 | 22.09400268 | 15.81594807 | 20.95573657 |
| LOC412406 | 5.524629497 | 4.541254379 | 4.227658944 | 4.028287374 |
| LOC100578774 | 11.05818504 | 16.59764036 | 19.60991533 | 17.24486965 |
| LOC724215 | 5.152847373 | 4.064176295 | 3.507005792 | 3.501862541 |
| LOC413388 | 3.873346328 | 3.451982192 | 2.965768239 | 2.621653681 |
| LOC411846 | 7.303954536 | 6.887527145 | 6.289548932 | 5.305725962 |
| LOC100577669 | 11.43449611 | 17.12758346 | 17.47886133 | 17.88580693 |
| LOC113218789 | 147.3049953 | 208.4244382 | 237.8075879 | 243.5403126 |
| LOC551906 | 5.742765551 | 4.392353924 | 4.332319817 | 4.213343296 |
| LOC100578040 | 2.373562128 | 1.552438095 | 1.298937178 | 1.549565053 |
| LOC113219394 | 5.205628355 | 3.746226382 | 2.71980791 | 3.401137296 |
| LOC409764 | 14.52222788 | 12.0887059 | 11.36449387 | 11.41740324 |
| LOC100578621 | 7.89413082 | 11.54425776 | 12.74999119 | 11.20681841 |
| LOC411613 | 10.11787349 | 13.57136541 | 15.22999847 | 16.86084832 |
| LOC551477 | 16.59984141 | 19.96686815 | 26.04808552 | 22.88353097 |
| LOC727237 | 4.646137556 | 5.962803189 | 8.590436262 | 6.927461125 |
| LOC413324 | 361.1869867 | 432.606635 | 509.113052 | 546.7275289 |
| LOC409225 | 30.57751113 | 40.7413812 | 51.13745108 | 44.35259876 |
| LOC409395 | 6.425262758 | 4.707828642 | 4.595509488 | 4.533456574 |
| LOC100578248 | 2.212079745 | 3.249346074 | 3.539180751 | 3.729976844 |
| LOC412388 | 5.631442519 | 7.106573429 | 10.07659092 | 8.275413169 |
| LOC409117 | 11.59157352 | 8.659874776 | 7.557016276 | 8.014093435 |
| LOC551613 | 20.25645358 | 16.58685114 | 15.10406399 | 15.16192125 |
| LOC102655153 | 0.073153015 | 1.313667206 | 0.720009989 | 0.550566023 |
| LOC412169 | 8.039709787 | 7.157837686 | 6.248871904 | 5.636696575 |
| LOC725881 | 143.3626407 | 162.4975331 | 199.8955296 | 197.1541865 |
| LOC725059 | 32.17524977 | 36.44035724 | 42.99070205 | 46.58021865 |
| LOC410980 | 9.878358964 | 7.54130982 | 6.133125668 | 6.839944432 |
| LOC410253 | 12.35270678 | 14.68781662 | 16.20819675 | 17.5523794 |
| - | 0.697617704 | 0.517414463 | 0.208988092 | 0.121179229 |
| LOC412103 | 11.61837561 | 9.888393057 | 8.55285545 | 8.745093549 |
| LOC102655220 | 13.87272478 | 17.05272093 | 17.13871772 | 19.85595878 |
| LOC408526 | 487.4862232 | 895.1604338 | 1206.649529 | 875.5584665 |
| LOC409341 | 135.954319 | 180.5418996 | 222.3440376 | 192.325615 |
| LOC410108 | 3.72527635 | 2.975264745 | 2.548576332 | 2.734728058 |
| LOC412093 | 16.05691292 | 15.13218202 | 12.24404791 | 12.74706949 |
| LOC411849 | 13.36699447 | 10.60279522 | 7.992817652 | 9.288120837 |
| LOC412780 | 4.772232758 | 4.241397324 | 3.361160268 | 3.474395866 |
| LOC551660 | 162.486565 | 175.8400726 | 217.1078524 | 213.8769698 |
| LOC413596 | 3.36849934 | 12.66797316 | 6.226730893 | 6.569982743 |
| LOC411985 | 7.670199119 | 5.935671743 | 5.220155849 | 5.84535659 |
| LOC551087 | 5.6012549 | 3.874558746 | 4.379077946 | 3.853899031 |
| LOC100578429 | 0.754686461 | 0.501041441 | 0.475221955 | 0.406164063 |
| LOC409649 | 9.604226313 | 12.17436606 | 11.00195782 | 14.69786833 |
| LOC725760 | 0.859166843 | 0.481627614 | 0.388320229 | 0.310366429 |
| LOC102654111 | 13.36405361 | 19.16243865 | 20.37720904 | 17.59116261 |
| LOC412278 | 8.251804851 | 6.767986664 | 5.408906708 | 6.173921807 |
| LOC411296 | 0.43546948 | 0.932176878 | 1.151696937 | 1.012322105 |
| LOC725761 | 2.07914573 | 1.729245439 | 1.548828423 | 1.294691122 |
| LOC552322 | 148.7098785 | 175.7332432 | 223.0888321 | 198.1870533 |
| LOC726548 | 13.93879643 | 10.27888793 | 8.226721304 | 9.602398884 |
| LOC100578600 | 15.66036892 | 19.04468552 | 23.89583088 | 23.44042014 |
| - | 8.596875108 | 6.38845873 | 4.397559095 | 5.998996544 |
| LOC409385 | 4.685551149 | 3.846688279 | 2.961134165 | 3.228013882 |
| LOC411468 | 10.62065074 | 10.4426458 | 8.670692662 | 8.389824549 |
| LOC550827 | 179.1595268 | 212.56332 | 312.6016619 | 274.3710286 |
| LOC410099 | 8.988339616 | 6.980058254 | 5.942895204 | 6.668973189 |
| LOC726562 | 8.489770213 | 7.833907439 | 6.331212093 | 6.197267311 |
| LOC409114 | 701.4263004 | 964.431246 | 1176.245654 | 982.7340031 |
| LOC726019 | 6.702091421 | 7.108140299 | 8.811872326 | 9.01555861 |
| LOC102654282 | 45.33216731 | 52.21967548 | 63.56959434 | 58.45518568 |
| LOC410511 | 11.63760081 | 10.92588094 | 7.83503148 | 9.071820092 |
| LOC411361 | 11.38426295 | 9.906675546 | 7.738640744 | 8.667052543 |
| LOC100576879 | 12.09685863 | 7.62115692 | 5.987260412 | 8.024331042 |
| LOC410905 | 37.51959495 | 62.9065734 | 61.21155826 | 51.79820732 |
| LOC724346 | 19.08908327 | 22.54958827 | 28.31281198 | 25.939662 |
| LOC725419 | 10.02613485 | 7.127209261 | 7.045225695 | 7.110305833 |
| LOC102656620 | 7.197529287 | 5.501005114 | 4.410791234 | 5.125740741 |
| LOC724486 | 20.86785202 | 26.27587618 | 33.5830088 | 30.58621456 |
| LOC411406 | 9.166332144 | 11.32104314 | 16.67308722 | 14.92574565 |
| LOC408656 | 8.15834369 | 6.17754771 | 4.748075941 | 5.760198178 |
| LOC411980 | 4.750935781 | 4.387952401 | 3.786323058 | 3.540045383 |
| LOC551459 | 22.64576489 | 31.67170815 | 47.72178011 | 37.56479702 |
| LOC413821 | 6.745822153 | 5.593761719 | 4.181674021 | 4.922152891 |
| LOC408316 | 4.476159648 | 2.962050483 | 2.285345535 | 3.001950613 |
| LOC410087 | 18.70174337 | 25.49014761 | 24.34826569 | 37.23797389 |
| LOC409451 | 208.5624452 | 261.8582022 | 309.2016183 | 268.9721966 |
| LOC107964876 | 6.213763986 | 7.303998976 | 10.08152844 | 9.559943089 |
| LOC411748 | 1.710997662 | 2.367292286 | 2.080628963 | 2.470424656 |
| LOC726916 | 2.439271818 | 1.911274809 | 1.951978965 | 1.705435489 |
| LOC410621 | 15.5680058 | 30.23308579 | 31.21293623 | 26.56198834 |
| LOC409242 | 25.35004719 | 22.86132156 | 17.85600034 | 20.44295313 |
| LOC552476 | 60.64367885 | 78.36478244 | 96.4083424 | 84.17447994 |
| LOC726339 | 2.731177938 | 1.831821902 | 1.595971361 | 1.827359101 |
| Smb | 43.90259605 | 49.5265553 | 66.18738672 | 60.39214591 |
| LOC411930 | 3.751409705 | 2.181898833 | 1.874617134 | 2.324990703 |
| LOC726063 | 5.048161359 | 3.946522938 | 3.373810999 | 3.550896018 |
| LOC724593 | 7.004657256 | 4.588698394 | 3.969113328 | 4.398130296 |
| LOC411483 | 19.53910706 | 15.45229247 | 15.10600139 | 14.88461474 |
| LOC408852 | 36.64379623 | 43.2875634 | 57.93245584 | 54.04677509 |
| LOC411691 | 4.939500988 | 7.55344935 | 9.08757205 | 7.311491987 |
| LOC411780 | 10.1675243 | 9.308603844 | 7.095960416 | 7.717589745 |
| LOC725835 | 17.67802951 | 25.77441601 | 31.03150759 | 27.66259019 |
| LOC411206 | 109.2886949 | 124.235447 | 158.9939922 | 145.6897854 |
| LOC413928 | 29.92826922 | 35.29193953 | 51.76782867 | 41.46674729 |
| LOC725115 | 60.95666257 | 36.91393825 | 32.16976053 | 39.76017194 |
| LOC409326 | 793.043514 | 1216.260268 | 1469.848912 | 1266.72064 |
| LOC100578662 | 7.66621956 | 8.268982805 | 11.46192964 | 10.87318454 |
| LOC410171 | 29.9858431 | 26.08004067 | 26.12348527 | 24.21001694 |
| LOC726962 | 11.08870202 | 19.22168416 | 18.88478759 | 18.52368409 |
| LOC102653832 | 5.296469777 | 6.989238225 | 10.62580115 | 8.841960249 |
| LOC409323 | 15.90262044 | 14.10703766 | 10.9392216 | 12.34984012 |
| LOC726120 | 354.3859639 | 361.6296576 | 429.4679849 | 468.5747001 |
| LOC102655388 | 10.57844166 | 13.69117732 | 19.07250735 | 15.86308209 |
| LOC100576167 | 1.242214333 | 2.239597866 | 2.239746568 | 2.540790776 |
| Or51 | 0.137598195 | 0.032344149 | 0.021201631 | 0 |
| LOC412593 | 9.732122655 | 9.170945691 | 8.468354111 | 7.47122237 |
| LOC409890 | 247.6764849 | 304.0288497 | 400.7346441 | 333.7181678 |
| LOC100577102 | 3.60002055 | 2.2796251 | 2.46422261 | 2.50846366 |
| LOC727230 | 5.971374543 | 4.979558769 | 3.813620659 | 4.642242078 |
| LOC551694 | 33.71605825 | 37.06204581 | 48.75886289 | 44.04023264 |
| LOC551773 | 6.572430922 | 6.094777066 | 5.351322222 | 4.779013386 |
| LOC413014 | 215.1171524 | 220.6936198 | 280.2042188 | 283.4557447 |
| LOC113218965 | 4.819004151 | 3.161301161 | 2.378010986 | 3.216393266 |
| LOC410821 | 57.88703803 | 76.30288165 | 82.99469455 | 85.15524679 |
| LOC410920 | 1.874018239 | 4.273249633 | 5.325557875 | 4.360064241 |
| LOC724319 | 3.602673352 | 4.401459369 | 5.587393495 | 5.506923953 |
| ATP5G2 | 1741.284673 | 1895.949975 | 2329.301087 | 2268.322376 |
| LOC724524 | 4.05132272 | 3.158072023 | 3.079414289 | 2.935075485 |
| LOC408836 | 24.36283889 | 28.26986159 | 35.62322634 | 34.59931593 |
| LOC408622 | 14.21174319 | 18.24236529 | 23.32731773 | 21.65091831 |
| LOC551498 | 4.785136359 | 3.067573755 | 2.512828687 | 3.282617598 |
| Msx | 0.690387864 | 0.869238161 | 1.403297657 | 1.292113521 |
| LOC411763 | 1.523669814 | 0.907217531 | 1.173125033 | 0.91246528 |
| LOC551511 | 21.15858476 | 30.12687975 | 32.82203147 | 29.14518363 |
| LOC410170 | 27.63712903 | 20.98983789 | 14.17856094 | 18.43523721 |
| LOC551505 | 38.50640947 | 52.68972274 | 69.61114617 | 57.55327006 |
| LOC100576307 | 3.318573776 | 4.219209398 | 5.210569163 | 4.807288258 |
| LOC408666 | 41.77951768 | 67.48905441 | 74.18565037 | 66.04882565 |
| LOC413957 | 4.783908707 | 3.752764235 | 3.127633977 | 3.413265477 |
| LOC726735 | 9.358265078 | 7.130495726 | 6.521510352 | 6.979792013 |
| LOC100578534 | 26.02755945 | 31.88069346 | 40.53523835 | 37.70979711 |
| LOC724779 | 32.08737666 | 51.30161123 | 45.81186914 | 47.11630343 |
| LOC725456 | 5.769835419 | 6.251160624 | 8.427094564 | 8.436539063 |
| LOC411253 | 8.202820341 | 15.43591064 | 13.42331171 | 12.33026927 |
| LOC410570 | 11.09026878 | 13.63039852 | 14.89926667 | 15.11766324 |
| LOC413578 | 10.76597443 | 8.234067435 | 6.386283767 | 7.64498808 |
| LOC727370 | 7.395996159 | 6.601966844 | 5.667000751 | 5.612464202 |
| LOC102653862 | 167.0991911 | 198.2022994 | 243.5080159 | 225.8909604 |
| LOC726699 | 1.077511201 | 0.973772663 | 0.820792237 | 0.699675513 |
| LOC113218521 | 10.64350551 | 15.27912383 | 17.21769385 | 15.22841219 |
| LOC410766 | 14.13993584 | 9.517597618 | 9.075961977 | 10.44177161 |
| LOC725538 | 47.38634447 | 51.56173985 | 64.8999977 | 63.26169158 |
| LOC102654640 | 6.484953708 | 4.817643106 | 5.576794736 | 4.714597787 |
| LOC113218576 | 2.90250739 | 6.117869487 | 5.744603871 | 8.456259263 |
| LOC725895 | 4.506635296 | 4.303483898 | 3.231366939 | 3.481894659 |
| LOC727150 | 3.583894779 | 2.574170546 | 2.106284862 | 2.683674159 |
| LOC100577376 | 6.114243049 | 6.883048868 | 8.053710757 | 8.659087139 |
| LOC100578556 | 3.160223469 | 2.772936257 | 2.082583026 | 2.359287841 |
| - | 4.307892719 | 3.456705865 | 3.635999838 | 2.871232557 |
| LOC411385 | 57.19320929 | 63.13753677 | 79.00013067 | 76.30638286 |
| LOC408695 | 536.1978477 | 594.5319774 | 677.3867772 | 705.5214234 |
| LOC725272 | 15.57486726 | 17.99792585 | 22.20241988 | 23.59074519 |
| LOC107964398 | 2.292404699 | 1.473198677 | 0.963210603 | 1.005236074 |
| LOC413524 | 6.986161793 | 5.658215712 | 4.777550705 | 5.367766328 |
| LOC726912 | 0.244053708 | 0.421923646 | 0.490551463 | 0.52538664 |
| LOC551966 | 12.4047463 | 15.88132344 | 21.46415686 | 17.46441115 |
| LOC411754 | 8.020697647 | 7.443815456 | 6.823371253 | 5.909703887 |
| LOC412885 | 17.99233989 | 32.28104096 | 34.9130001 | 27.05088519 |
| LOC726369 | 14.96528663 | 16.3730076 | 21.67910082 | 20.08022939 |
| LOC726469 | 3.450635375 | 5.367212652 | 6.132167357 | 5.876089805 |
| LOC412217 | 16.91162524 | 13.87306545 | 10.99197499 | 12.04429913 |
| LOC550706 | 12.36119482 | 10.2227823 | 8.068849342 | 9.751674103 |
| LOC107964212 | 2.257777011 | 1.558996511 | 1.425390832 | 1.441104551 |
| LOC102655111 | 4.990318483 | 10.08317521 | 9.400795036 | 8.507609435 |
| LOC100576958 | 6.701664668 | 5.633899029 | 4.00620153 | 5.044741175 |
| LOC100576326 | 180.0251139 | 300.829571 | 286.6447934 | 251.3702524 |
| LOC726493 | 3.371872634 | 2.162060397 | 1.897708682 | 2.029195093 |
| Tpx-4 | 91.78365186 | 153.1928135 | 178.7036933 | 146.9223929 |
| LOC552809 | 603.3078021 | 662.6793449 | 756.4484663 | 815.6118087 |
| LOC408780 | 5.425278567 | 6.58177897 | 7.90923984 | 7.510378312 |
| LOC409098 | 4.797650078 | 3.094588364 | 3.149927569 | 3.464264791 |
| LOC412564 | 12.91841286 | 11.36973399 | 10.34922176 | 10.33594205 |
| LOC410558 | 5.150606281 | 3.218427186 | 2.839822121 | 3.294148304 |
| LOC725718 | 151.1038795 | 167.8298604 | 212.2882153 | 189.4735348 |
| LOC550992 | 44.34464561 | 49.51186754 | 62.66788904 | 59.81382669 |
| LOC551882 | 122.1693701 | 149.4621694 | 176.0245301 | 170.8115863 |
| LOC409189 | 135.1951733 | 181.2505383 | 219.0043874 | 184.5434585 |
| LOC107963964 | 6.527819226 | 5.10485819 | 5.268132562 | 4.129573566 |
| LOC413438 | 9.051328676 | 7.02219013 | 5.758998339 | 6.716028776 |
| LOC409628 | 14.72638367 | 21.73206842 | 24.7452932 | 21.06876322 |
| LOC550822 | 5.305542934 | 4.626031726 | 4.718768061 | 3.856510295 |
| LOC410552 | 2.732698839 | 3.248031739 | 4.649269166 | 5.080964603 |
| LOC552022 | 10.72580093 | 7.407668397 | 5.756951264 | 7.622635947 |
| - | 212.3334354 | 271.0600608 | 319.7921641 | 282.7876767 |
| LOC552031 | 9.542564959 | 7.822283899 | 6.16936819 | 7.308353166 |
| LOC113218605 | 10.50966402 | 8.721288304 | 6.154663444 | 7.599108151 |
| LOC412688 | 7.218428929 | 5.670617259 | 6.188730501 | 5.385081397 |
| LOC410867 | 15.45519701 | 19.63356564 | 19.38701275 | 22.72341729 |
| LOC727131 | 9.898580922 | 11.0497214 | 15.78662635 | 17.83073552 |
| LOC414043 | 6.437475987 | 3.889394584 | 3.754492119 | 4.392516771 |
| Eaat-2 | 31.30800194 | 43.23334789 | 40.36414211 | 41.72870081 |
| LOC100578560 | 60.50611733 | 67.9126149 | 83.87754515 | 83.98076074 |
| LOC412035 | 2.270032305 | 1.353057828 | 1.157675217 | 1.370229232 |
| LOC409552 | 742.9004446 | 992.0694147 | 1235.682574 | 1120.875553 |
| ecd | 5.116564447 | 4.126677701 | 3.719896182 | 3.855487958 |
| LOC726989 | 7.712942468 | 5.938074691 | 5.480976478 | 5.91301982 |
| LOC724793 | 110.4619135 | 133.728439 | 173.4502522 | 151.1118315 |
| LOC102653655 | 6.896446133 | 12.51784069 | 12.92826557 | 11.02233462 |
| LOC724214 | 7.831094562 | 6.321484958 | 6.031225098 | 6.204066438 |
| LOC411612 | 6.005021598 | 5.783011675 | 5.116009891 | 4.775133763 |
| LOC552641 | 73.6777712 | 84.91894174 | 107.3396214 | 103.1230091 |
| LOC410040 | 7.523056173 | 6.599227348 | 6.121154517 | 5.682255497 |
| LOC726601 | 13.94891794 | 10.71808705 | 9.146374518 | 10.21099349 |
| LOC408789 | 24.07133418 | 31.62973824 | 30.28292646 | 33.2898176 |
| LOC724244 | 2.922624441 | 2.395717076 | 2.255176165 | 2.067082623 |
| LOC726220 | 9.792511076 | 14.5831885 | 18.68920459 | 16.66684375 |
| LOC413551 | 17.42633595 | 13.70928978 | 12.53994326 | 12.96214277 |
| LOC413548 | 10.12564017 | 7.430341181 | 6.265399344 | 7.517623367 |
| Dcr-1 | 4.260869137 | 2.30391403 | 1.941830178 | 2.469608052 |
| LOC726525 | 5.966247177 | 5.10619456 | 4.718602309 | 4.202706614 |
| LOC100578725 | 35.79803025 | 37.68176174 | 49.20299873 | 48.41752924 |
| LOC413074 | 9.0642046 | 7.869742862 | 5.85446708 | 6.651420068 |
| LOC409838 | 4.519255017 | 3.723009072 | 3.071489618 | 3.324383448 |
| InR-2 | 0.298974254 | 0.51569248 | 0.567853742 | 0.553595667 |
| LOC409179 | 7.03523443 | 4.347025587 | 3.854321557 | 4.727760368 |
| LOC107964343 | 12.14473237 | 14.7748864 | 15.13783711 | 15.80262396 |
| LOC113218757 | 2.423845132 | 5.828102538 | 3.669732446 | 7.021246474 |
| LOC411338 | 1.366199637 | 1.105547216 | 1.065030246 | 0.950806041 |
| LOC411362 | 10.08305875 | 7.006687 | 6.455912616 | 7.485272555 |
| LOC727311 | 9.053419147 | 6.024945136 | 5.142138407 | 6.125850034 |
| LOC724899 | 18.09969724 | 22.46994984 | 31.59476367 | 31.70887538 |
| LOC411640 | 5.17722842 | 2.722277466 | 2.744691075 | 3.064557582 |
| LOC725977 | 38.33275889 | 41.88521499 | 54.42807737 | 50.80088242 |
| LOC412985 | 7.656533707 | 5.742674314 | 6.246701418 | 5.6406812 |
| LOC410776 | 2.560069829 | 3.323591695 | 4.000102287 | 3.686771826 |
| LOC413368 | 1.58863641 | 1.320516515 | 1.271201076 | 1.078228501 |
| LOC726651 | 2.262340927 | 1.286954032 | 1.323197153 | 1.502356316 |
| LOC411272 | 130.668785 | 139.7459678 | 198.1602261 | 171.6320801 |
| LOC411989 | 223.857705 | 320.5947173 | 385.7889396 | 316.3064126 |
| LOC413263 | 1.849965475 | 3.236155771 | 3.092002413 | 2.906988314 |
| LOC727238 | 3.16535977 | 2.600601175 | 1.855371649 | 2.355177746 |
| LOC100577367 | 3.432473728 | 3.22293348 | 2.253289724 | 2.584460796 |
| LOC409235 | 59.37372475 | 75.93794928 | 76.59262352 | 77.64400181 |
| LOC409620 | 6.676784757 | 5.252899906 | 4.225949135 | 4.424712836 |
| LOC113218892 | 40.27570362 | 47.57109351 | 59.74928239 | 56.81792352 |
| LOC552019 | 1.513729754 | 0.832928254 | 0.579200667 | 0.822790215 |
| LOC408661 | 1.643279735 | 2.180413124 | 2.265597126 | 2.411538721 |
| LOC411420 | 4.466622287 | 3.566256442 | 2.518454547 | 2.866190291 |
| LOC727568 | 53.39885005 | 73.23412862 | 102.2170361 | 94.42860066 |
| Tret1 | 32.31903883 | 50.98134752 | 45.26395511 | 45.85190478 |
| LOC411734 | 20.27059945 | 16.75310995 | 15.65830167 | 15.54852068 |
| LOC408332 | 5.971492154 | 5.337909161 | 4.029775079 | 4.784311833 |
| LOC552247 | 30.67855333 | 32.18687708 | 21.13018809 | 22.55288831 |
| LOC100577341 | 35.31261379 | 43.02177626 | 52.55534445 | 49.78167438 |
| LOC551652 | 46.2437405 | 50.58157065 | 57.30494822 | 60.44985702 |
| LOC551364 | 1.653125424 | 2.574943299 | 2.661336737 | 2.551724708 |
| LOC102656183 | 81.79143461 | 105.7757726 | 134.3546246 | 106.9496541 |
| LOC550977 | 7.515526094 | 6.062433493 | 4.809677814 | 5.333579533 |
| LOC409556 | 8.028910498 | 5.474391892 | 5.002504205 | 5.428270711 |
| LOC724144 | 57.56033291 | 76.91618805 | 100.5237303 | 82.88401312 |
| LOC410769 | 19.72628131 | 25.53375449 | 37.41244917 | 31.14799193 |
| LOC100577603 | 29.03230356 | 34.27320835 | 48.87360531 | 43.26243082 |
| LOC100576346 | 7.513199158 | 5.004057 | 4.127387384 | 4.848733404 |
| LOC726269 | 95.38191936 | 113.2846639 | 137.3532506 | 121.9914969 |
| LOC414039 | 8.150978643 | 9.581536064 | 14.93183671 | 12.32286217 |
| LOC413512 | 8.081677479 | 6.687878778 | 6.505777306 | 5.365614876 |
| LOC725675 | 11.31134868 | 15.51757115 | 15.93406401 | 16.24480454 |
| LOC409452 | 17.06380644 | 16.38275217 | 13.95193188 | 13.66367139 |
| LOC413176 | 4.715766936 | 2.652215056 | 2.940644853 | 3.269440263 |
| LOC725350 | 4.45704739 | 9.044499455 | 12.33366873 | 9.268281367 |
| LOC409468 | 25.17667445 | 28.39546105 | 33.15342761 | 32.44664019 |
| LOC100579049 | 5.030322262 | 4.16699537 | 4.048977921 | 3.949141412 |
| LOC100578823 | 7.309037861 | 3.898554231 | 3.63642688 | 4.48909635 |
| LOC102656652 | 37.73912733 | 42.51709232 | 61.25026545 | 52.07624033 |
| LOC724885 | 3.502307935 | 6.335316405 | 5.144574208 | 5.857506039 |
| LOC412408 | 5.449418868 | 4.001422768 | 4.030223431 | 3.76788481 |
| LOC100577237 | 6.143376294 | 4.439747954 | 3.326714946 | 4.095761822 |
| LOC725592 | 9.66666447 | 12.26698285 | 14.39151857 | 13.35742989 |
| LOC102656137 | 7.875541189 | 6.232875316 | 6.575342902 | 5.868942136 |
| LOC725150 | 11.1851362 | 8.296453722 | 7.321421207 | 8.55636587 |
| LOC100577268 | 4.730214194 | 6.460377358 | 7.883724618 | 6.706241506 |
| LOC100578661 | 3.578940147 | 2.631457622 | 2.260565301 | 2.678469545 |
| LOC552833 | 11.32304805 | 10.88398594 | 8.669544572 | 8.91657196 |
| LOC413605 | 181.3616449 | 218.4563254 | 255.5708793 | 231.8643616 |
| LOC552811 | 4.338109856 | 8.356616684 | 10.37541961 | 8.386747027 |
| LOC724719 | 106.8268647 | 118.9521434 | 130.7177935 | 145.6675186 |
| LOC409527 | 8.68638908 | 6.971506625 | 6.015654813 | 6.691710382 |
| LOC408677 | 17.51707471 | 22.10713945 | 26.06721177 | 26.28372951 |
| LOC552433 | 9.224627608 | 7.63798066 | 6.871981678 | 7.15824564 |
| LOC725960 | 401.1159003 | 432.8892901 | 449.4054512 | 572.66043 |
| LOC551515 | 20.72353548 | 17.35764477 | 15.40727021 | 16.64083082 |
| LOC413977 | 37.4001742 | 40.7975808 | 39.01378086 | 46.28039599 |
| LOC410600 | 4.120062847 | 5.225932265 | 6.327525762 | 5.778840477 |
| LOC409724 | 11.49320449 | 10.60534714 | 9.044764274 | 8.728062547 |
| - | 4.620438846 | 4.390639518 | 3.181748969 | 3.23481022 |
| LOC410820 | 3.150022237 | 3.782400971 | 3.640286008 | 4.171576849 |
| LOC413547 | 20.74060036 | 17.2958149 | 15.00712532 | 15.96254494 |
| LOC411351 | 19.45868963 | 16.32252003 | 16.27247156 | 14.31588091 |
| LOC550915 | 21.78577877 | 25.94200301 | 29.04589784 | 27.90916145 |
| LOC413195 | 5.510920498 | 4.092537433 | 3.40783209 | 3.953616135 |
| LOC550671 | 57.94039945 | 132.437556 | 129.3943569 | 106.5321631 |
| LOC107964839 | 1.951115351 | 1.189078842 | 1.167371125 | 1.280469283 |
| LOC410004 | 66.5339582 | 82.99117457 | 109.7087566 | 90.57337605 |
| LOC727199 | 107.651158 | 119.7846177 | 146.8273323 | 140.814858 |
| LOC410073 | 8.254458249 | 5.458295606 | 4.663491861 | 5.339847111 |
| LOC102654691 | 276.6535101 | 313.8486864 | 456.8855132 | 415.7569173 |
| LOC409613 | 73.04595647 | 90.65159831 | 116.9397813 | 95.70567686 |
| LOC726270 | 487.6665059 | 714.5604505 | 942.7948477 | 799.3028715 |
| LOC413592 | 12.38033875 | 7.346219666 | 6.864668057 | 8.167778321 |
| LOC552124 | 54.87927007 | 82.9350584 | 85.7401279 | 76.9296103 |
| LOC552028 | 11.97588794 | 12.26542947 | 15.19009439 | 16.12369182 |
| LOC408797 | 40.1658967 | 48.87042013 | 53.34729446 | 57.22727593 |
| LOC410280 | 56.78491954 | 78.79896226 | 112.847351 | 85.77167398 |
| LOC100576945 | 71.5332821 | 79.01385615 | 93.35640836 | 92.86124541 |
| LOC411265 | 7.261029825 | 5.131876269 | 4.383679715 | 4.976115838 |
| LOC725269 | 3.213487169 | 2.664091675 | 2.276376646 | 2.270269282 |
| LOC410044 | 6.102663981 | 7.906503854 | 9.546407375 | 8.70309946 |
| LOC552814 | 8.330466955 | 5.610572631 | 4.656394968 | 5.421346416 |
| LOC409010 | 8.355436377 | 8.096237861 | 6.334041225 | 6.39059185 |
| LOC112935903 | 12.0889819 | 7.99076842 | 6.971335145 | 7.426120426 |
| LOC411209 | 8.447875917 | 5.809629977 | 5.835073098 | 5.700076227 |
| LOC100577707 | 105.2216234 | 74.10096695 | 68.80820472 | 78.25288421 |
| LOC409082 | 6.530843422 | 5.441315351 | 4.843610952 | 4.717644844 |
| LOC551684 | 520.9794177 | 622.2949102 | 724.3039278 | 654.0959148 |
| LOC727272 | 10.76741536 | 8.795226784 | 9.130394961 | 7.878294796 |
| LOC412896 | 2.84716765 | 2.509824764 | 2.105284152 | 2.013083328 |
| LOC409647 | 11.30081605 | 7.30662475 | 7.591934892 | 7.921190575 |
| LOC552134 | 71.05290389 | 92.78617488 | 124.3519557 | 101.0383525 |
| LOC410733 | 143.9424302 | 211.289992 | 248.9924779 | 219.5472109 |
| LOC406097 | 24.96677413 | 24.03009373 | 18.43041417 | 18.48591993 |
| LOC412749 | 13.08166916 | 12.52753796 | 10.75650358 | 10.6385313 |
| LOC410456 | 4.093844992 | 7.214252851 | 6.746667014 | 6.152440352 |
| LOC411183 | 346.2188709 | 382.6517044 | 470.6967939 | 435.6871708 |
| LOC408473 | 3.988447445 | 4.321082212 | 4.579273061 | 5.737090112 |
| LOC551806 | 18.41590047 | 24.3446306 | 28.28488487 | 26.05345858 |
| LOC413955 | 9.410957462 | 8.143742748 | 7.314361408 | 7.467865944 |
| Gat-1B | 35.65573266 | 44.04122938 | 44.67919121 | 45.29834957 |
| LOC408327 | 63.02064647 | 82.19147739 | 91.39924161 | 91.35805666 |
| LOC413660 | 13.28363892 | 9.029476277 | 9.634117338 | 9.788966403 |
| LOC551494 | 22.97098457 | 18.7190532 | 13.71258612 | 16.33910431 |
| LOC726656 | 24.51536726 | 16.91421704 | 12.85714817 | 16.41218078 |
| GstS1 | 18.80352834 | 21.79300358 | 24.9296984 | 25.06045186 |
| LOC724286 | 41.05156768 | 52.68016158 | 62.1558243 | 54.93842009 |
| LOC100576972 | 6.055084164 | 4.771153466 | 3.971161829 | 4.610511603 |
| LOC408510 | 2.579554099 | 3.554960624 | 2.908887529 | 3.830533871 |
| - | 13.36774195 | 17.66171607 | 23.61810194 | 19.62971606 |
| LOC412756 | 5.681394364 | 2.980715917 | 2.729611203 | 3.470569858 |
| LOC552580 | 4.059055282 | 2.789914899 | 2.803704518 | 2.9197254 |
| LOC551637 | 8.982983567 | 7.679646427 | 7.131140327 | 6.994427648 |
| LOC413126 | 3.567386627 | 3.513007406 | 2.860687348 | 2.635631328 |
| LOC412396 | 417.4112321 | 482.1824985 | 558.6180522 | 532.1168213 |
| LOC409599 | 554.2868594 | 702.1534078 | 903.2595414 | 822.3385713 |
| LOC725983 | 8.790314147 | 5.516535694 | 5.352748754 | 5.849500998 |
| LOC410771 | 4.069137263 | 6.667372699 | 7.738244716 | 6.830281215 |
| LOC725550 | 18.69292138 | 15.85489218 | 13.67602866 | 14.72619669 |
| LOC727294 | 32.34084033 | 34.45061839 | 39.25534214 | 44.35296537 |
| LOC100578498 | 3.480481666 | 2.662575082 | 2.380319251 | 2.414819128 |
| LOC408616 | 5.035409134 | 4.125967868 | 3.723526033 | 3.996562676 |
| LOC726818 | 4.543378262 | 10.91558807 | 5.970172778 | 7.197091436 |
| LOC410780 | 3.74526077 | 7.195894905 | 6.595916411 | 5.848966481 |
| LOC408398 | 2.311203096 | 2.485883244 | 2.963746328 | 3.562302843 |
| LOC725480 | 16.66647193 | 23.09649471 | 27.0584311 | 22.6145432 |
| LOC409102 | 33.02497411 | 22.42664742 | 17.71467305 | 22.39541771 |
| LOC552172 | 2.248360293 | 1.595285407 | 1.326779063 | 1.558165431 |
| LOC408446 | 236.9671343 | 275.5494225 | 318.6941094 | 342.146584 |
| LOC411746 | 1.060752138 | 1.535368812 | 1.555069078 | 1.682157348 |
| LOC100578734 | 12.87979121 | 15.03929731 | 16.97275732 | 17.15174482 |
| LOC727121 | 3.074211383 | 5.022376734 | 6.301563212 | 5.104592668 |
| LOC100578563 | 2.766236326 | 1.915931835 | 1.315699645 | 1.857265899 |
| LOC551852 | 60.75056184 | 80.51880409 | 95.1429452 | 85.50688025 |
| LOC408361 | 43.88955973 | 100.4154076 | 85.85218285 | 70.90657062 |
| LOC551881 | 12.41274162 | 11.35821583 | 9.299425539 | 9.225775055 |
| LOC413233 | 33.17087304 | 20.78449942 | 19.97059707 | 23.48510839 |
| LOC413785 | 33.62420871 | 59.04352066 | 86.63314251 | 59.71268189 |
| LOC727108 | 16.46904456 | 13.44173501 | 10.52862555 | 12.03334616 |
| LOC726407 | 26.84672953 | 33.20282731 | 35.96561694 | 35.80624842 |
| - | 0.434046909 | 0.316501323 | 0.362088342 | 0.194638248 |
| LOC411759 | 10.58281809 | 8.480769984 | 7.022844694 | 8.379569733 |
| LOC727358 | 9.226610287 | 7.376649181 | 6.03225115 | 6.802639423 |
| LOC410304 | 0.546179789 | 0.981506903 | 0.956632873 | 0.909983155 |
| LOC100577504 | 41.81955669 | 59.95835721 | 65.65127207 | 62.03979482 |
| LOC724545 | 4.615667643 | 4.036130714 | 3.095353792 | 3.425206275 |
| LOC412459 | 14.44047442 | 12.41348441 | 9.463193708 | 10.38392632 |
| LOC100576876 | 9.180895361 | 7.40409968 | 5.51811987 | 6.455720052 |
| LOC409158 | 5.09229204 | 9.266789951 | 10.56175405 | 7.700200888 |
| LOC551794 | 37.71647345 | 43.06619263 | 51.28071738 | 49.80999888 |
| LOC410289 | 20.46385622 | 17.55134782 | 13.83648567 | 14.63641035 |
| LOC409801 | 4.955902392 | 42.89536741 | 8.519220868 | 7.821175916 |
| LOC102656248 | 0.785198649 | 1.025128685 | 1.011950265 | 1.256574814 |
| LOC552664 | 11.12310541 | 9.20213589 | 7.978174548 | 8.60212067 |
| LOC726670 | 12.31971799 | 10.3928026 | 9.44307319 | 9.950586305 |
| LOC410849 | 12.14700272 | 10.36343295 | 9.694902682 | 9.617149683 |
| LOC409977 | 5.441920855 | 4.10020305 | 3.201857276 | 3.880394718 |
| LOC724994 | 2.265763747 | 2.751275857 | 4.058690373 | 3.831607923 |
| LOC412027 | 6.558747095 | 4.998229037 | 4.517974346 | 5.013146538 |
| LOC724971 | 10.36875722 | 11.33376345 | 13.00165098 | 14.66703136 |
| LOC551548 | 9.847300556 | 8.339841351 | 6.487476916 | 7.643741065 |
| Emc | 40.66607032 | 51.79533563 | 46.66542148 | 53.40922858 |
| LOC725500 | 5.702953807 | 6.423772915 | 8.05360455 | 8.518174713 |
| LOC551375 | 13.13336804 | 11.74289614 | 7.480348092 | 9.588595889 |
| LOC551207 | 6.928700973 | 5.259861014 | 4.794290742 | 4.791670603 |
| LOC100577178 | 0.049688333 | 0.029779741 | 0.022137141 | 0 |
| DHRS4 | 36.76468534 | 43.8631552 | 54.31847954 | 49.96325115 |
| LOC408345 | 12.07627653 | 10.52965483 | 8.761082881 | 9.451929333 |
| LOC551825 | 4.535384091 | 4.106419026 | 3.446751925 | 3.378497472 |
| LOC102654426 | 368.016183 | 666.845259 | 873.8065054 | 672.4087805 |
| Dll | 2.822184747 | 4.101876738 | 3.973378944 | 3.944992486 |
| LOC413133 | 6.73957549 | 6.637943309 | 4.644170962 | 5.076497716 |
| LOC727021 | 0.590394625 | 1.039499117 | 0.876149315 | 1.459886037 |
| LOC409333 | 5.121385268 | 9.613398187 | 10.17303186 | 7.504348683 |
| LOC725704 | 4.500799606 | 3.041102826 | 2.742797536 | 3.160020622 |
| LOC724599 | 7.989536498 | 7.924266642 | 6.842549817 | 6.633861241 |
| LOC725092 | 1.182354439 | 2.000070256 | 2.55556318 | 2.231152795 |
| LOC724943 | 18.73255031 | 19.93945505 | 26.54869977 | 24.37184355 |
| LOC725613 | 5.496426707 | 3.218023751 | 2.572241223 | 3.544898252 |
| LOC551664 | 45.08464764 | 61.07729692 | 66.66026845 | 59.69995286 |
| LOC413351 | 7.313528957 | 6.609300803 | 6.906264748 | 5.854383485 |
| LOC552421 | 17.79222671 | 16.87903474 | 12.22628368 | 14.46306597 |
| LOC100578386 | 2.450684632 | 1.289432832 | 1.136548415 | 1.640789597 |
| LOC551805 | 11.59182371 | 12.06592085 | 16.75677776 | 17.33970053 |
| Ecr | 3.357499624 | 5.221842327 | 4.88276469 | 5.165552705 |
| LOC102656492 | 2.274273535 | 1.464228162 | 1.524802827 | 1.409461977 |
| MsrA | 35.86578191 | 38.71714551 | 39.24094057 | 49.57342306 |
| LOC409236 | 296.4189322 | 363.9450526 | 406.4977167 | 371.6502026 |
| - | 13.66977291 | 22.39586289 | 22.78381937 | 38.9058997 |
| LOC100577913 | 12.59909713 | 11.64311076 | 8.059923551 | 9.870654557 |
| LOC411021 | 2.007779847 | 3.0119898 | 3.679745513 | 3.427234805 |
| LOC727285 | 3.286348975 | 2.712294877 | 1.870892448 | 2.193129372 |
| LOC100576446 | 1.971093403 | 4.402704659 | 6.419663577 | 3.882960097 |
| LOC412161 | 2.485954952 | 3.497326838 | 3.052880503 | 3.736949545 |
| LOC410062 | 12.04680947 | 17.83289431 | 18.64415378 | 20.09984063 |
| LOC408918 | 69.19717004 | 109.6728376 | 125.8533901 | 108.1460565 |
| LOC726771 | 9.406631724 | 8.359839195 | 6.394477257 | 6.567001173 |
| LOC725562 | 2.238391092 | 1.541581981 | 1.736441405 | 1.45866699 |
| LOC724141 | 76.17575227 | 100.8153926 | 99.1966485 | 98.31088923 |
| Hex70a | 47.05369585 | 70.4973221 | 89.65770074 | 72.1712934 |
| LOC552103 | 3.680865119 | 4.026191374 | 7.328887358 | 5.532928372 |
| LOC551486 | 12.76702728 | 12.24518509 | 9.638752673 | 10.45461126 |
| - | 1.897625035 | 1.873847326 | 3.931207056 | 6.458711114 |
| LOC552129 | 2.872753998 | 1.963572013 | 1.634190095 | 1.980920963 |
| B-gluc1 | 34.2209348 | 43.06045543 | 46.44723216 | 43.44848242 |
| LOC409645 | 24.52725868 | 28.01343865 | 38.67550885 | 31.46150667 |
| LOC551169 | 134.5645206 | 160.3259233 | 190.2121512 | 168.032702 |
| LOC552743 | 10.80350423 | 13.49181894 | 16.92119611 | 15.78120215 |
| - | 0.289346959 | 0.065513977 | 0 | 0.014257619 |
| LOC411525 | 93.31904372 | 80.96033482 | 72.63982752 | 76.56133243 |
| LOC408280 | 54.13065548 | 109.99938 | 71.52346979 | 83.82070153 |
| - | 0.243153764 | 0.416218135 | 0.438821865 | 0.518566198 |
| LOC412251 | 2.305034081 | 1.737604829 | 1.540018237 | 1.659577825 |
| LOC408565 | 12.64986874 | 12.30842869 | 10.03437161 | 9.88696406 |
| LOC725422 | 13.00055787 | 21.07124458 | 27.32324277 | 20.84783013 |
| LOC100576321 | 1.136783123 | 1.094462061 | 0.804087787 | 0.845121233 |
| LOC551497 | 11.65883729 | 15.13438572 | 17.63101408 | 15.90409301 |
| LOC413345 | 4.89950613 | 4.104301439 | 3.566150137 | 3.602759079 |
| LOC410120 | 67.51642101 | 67.77411861 | 84.42283884 | 83.31498298 |
| LOC100578674 | 2.101313426 | 1.843478389 | 2.102706015 | 1.441793818 |
| LOC412721 | 5.075095978 | 4.651563801 | 4.195494713 | 4.045774614 |
| LOC113218545 | 1.14696768 | 1.775850837 | 1.662868186 | 2.014082178 |
| LOC107964338 | 5.674924589 | 5.296606937 | 7.292130091 | 8.08573466 |
| LOC408815 | 2.959916424 | 2.092024533 | 1.635912346 | 2.015141778 |
| LOC726636 | 7.378948207 | 5.386779978 | 4.954091077 | 5.475038939 |
| LOC725899 | 37.60858552 | 46.62367102 | 50.98491257 | 47.79704807 |
| LOC408481 | 35.2838007 | 51.4719768 | 60.98494886 | 50.24060169 |
| LOC408418 | 60.7429652 | 72.40706365 | 86.42399852 | 77.21671341 |
| LOC724721 | 100.3336655 | 94.80098554 | 158.4570263 | 140.0699195 |
| LOC408508 | 4.89835881 | 5.188995571 | 6.970523673 | 6.992505933 |
| LOC552579 | 67.38581555 | 70.97367514 | 93.49135267 | 87.30393028 |
| LOC410968 | 3.119191902 | 2.655322263 | 2.420816173 | 2.260853975 |
| LOC724634 | 15.68448634 | 11.8852978 | 10.52365201 | 12.24343062 |
| LOC100577696 | 1.091627671 | 1.169009947 | 1.350474376 | 1.707269873 |
| LOC411380 | 427.1355719 | 594.4176705 | 712.5584571 | 649.9067542 |
| LOC406073 | 2.036654966 | 3.157650248 | 3.018937254 | 3.325730332 |
| LOC413060 | 0.697667415 | 1.733112728 | 1.443375155 | 1.347341144 |
| LOC552280 | 8.892067811 | 6.334741185 | 5.726408219 | 6.5382201 |
| LOC102654405 | 0.062665049 | 0.168609884 | 0.317454791 | 0.359150404 |
| LOC413997 | 77.02240445 | 86.19926828 | 106.8832841 | 100.630976 |
| LOC408302 | 7.727886105 | 18.58743389 | 16.83226084 | 12.87037495 |
| LOC100577778 | 0.605029899 | 1.405871898 | 0.965156294 | 1.063431185 |
| LOC100577397 | 10.40570147 | 11.68925497 | 16.8286801 | 14.86341242 |
| LOC408811 | 18.30273645 | 15.22155918 | 9.915285577 | 13.35043762 |
| LOC552118 | 16.27675913 | 19.74552313 | 22.62924252 | 21.39751173 |
| LOC102655778 | 13.02096438 | 9.231145267 | 11.23256211 | 10.10078253 |
| LOC411366 | 8.734854511 | 12.03030564 | 16.19389333 | 13.04576336 |
| LOC408989 | 21.95860087 | 30.88904001 | 36.46120773 | 34.10957086 |
| LOC413341 | 10.65355054 | 8.632406209 | 8.048154809 | 8.492409981 |
| LOC726862 | 7.496385434 | 5.138908459 | 4.779239916 | 5.411947221 |
| LOC411365 | 16.05098842 | 26.30107582 | 32.36823944 | 23.55426042 |
| LOC724423 | 1.466992593 | 0.854061775 | 0.797970087 | 0.866184476 |
| LOC408464 | 4.860522908 | 3.795875982 | 4.290237361 | 3.772210627 |
| LOC410402 | 2.075248776 | 1.933562488 | 1.657852271 | 1.510733404 |
| LOC408449 | 7.406934491 | 11.72346198 | 9.262616467 | 10.18860286 |
| LOC107964405 | 6.724457866 | 7.323047405 | 9.825612657 | 10.70006113 |
| LOC408382 | 3.901720939 | 2.696990513 | 2.090385117 | 2.489275467 |
| LOC413692 | 5.18997326 | 4.491360996 | 4.065137219 | 3.81941937 |
| LOC113219369 | 0.163726705 | 0.298333838 | 0.591440848 | 0.463002399 |
| LOC412789 | 42.9184687 | 33.863833 | 28.21264373 | 32.65618651 |
| LOC409023 | 7.532807543 | 8.676838007 | 14.37354708 | 12.92158401 |
| LOC410565 | 13.16469109 | 11.58730552 | 9.94428111 | 10.49202417 |
| LOC100576159 | 0.657974068 | 0.254340971 | 0.265484857 | 0.298128496 |
| LOC410793 | 3.868461658 | 4.674723766 | 6.756218587 | 5.916718863 |
| LOC113218866 | 7.937557031 | 4.024608817 | 3.625425128 | 5.06662748 |
| LOC409259 | 8.173285786 | 6.912139817 | 5.684673712 | 6.084458542 |
| LOC408634 | 13.05730947 | 15.98912678 | 18.09756471 | 16.78909702 |
| LOC724380 | 12.17738416 | 10.35113964 | 8.170125208 | 9.067877602 |
| LOC412541 | 16.22609608 | 16.85589599 | 20.79546799 | 20.84333445 |
| LOC550801 | 13.8631801 | 9.445777708 | 7.197878145 | 9.870090322 |
| LOC408452 | 55.27818624 | 75.13938999 | 86.26937687 | 78.82647484 |
| LOC100577797 | 5.289897639 | 8.068650269 | 11.21661188 | 8.728296024 |
| LOC412288 | 11.23545926 | 10.82968912 | 7.997376665 | 8.87551735 |
| LOC412279 | 2.669239709 | 2.25383515 | 2.014267227 | 1.816755542 |
| LOC408504 | 16.99070207 | 17.51745739 | 22.99606046 | 23.6545627 |
| LOC100577735 | 19.94584766 | 12.47897642 | 12.33044964 | 13.51136683 |
| LOC102656021 | 10.47292384 | 9.466320206 | 7.091046017 | 8.341778359 |
| LOC726205 | 13.44663779 | 17.11309601 | 22.2961426 | 20.03155017 |
| LOC100576404 | 12.24376638 | 14.88753604 | 22.61724345 | 18.19480113 |
| LOC552654 | 66.32748668 | 72.55097953 | 88.45919433 | 82.92079991 |
| LOC100577394 | 1.76368027 | 1.507601225 | 1.272493701 | 0.987362095 |
| LOC100576795 | 7.561177301 | 7.029729709 | 6.030511935 | 5.851782532 |
| LOC408400 | 57.29782611 | 65.60482858 | 86.21548197 | 75.41608464 |
| LOC410017 | 21.43493163 | 24.09206637 | 35.85399307 | 29.08156346 |
| LOC725578 | 19.06660754 | 19.53922877 | 22.86260385 | 24.60078146 |
| LOC410039 | 21.50989666 | 24.87905326 | 28.88045278 | 28.48752317 |
| LOC413029 | 15.83329099 | 12.62332389 | 9.494780603 | 11.61440948 |
| LOC725963 | 0.857535587 | 0.521074979 | 0.420464811 | 0.478990222 |
| LOC726721 | 145.1168479 | 179.5806276 | 251.78305 | 243.3838074 |
| LOC113219049 | 5.076493961 | 4.284330001 | 3.55887764 | 3.750271846 |
| LOC410589 | 8.130843565 | 10.09968235 | 12.27679107 | 11.28445441 |
| LOC724853 | 1.615922766 | 2.227192219 | 2.087307497 | 2.208213447 |
| CYTB | 2676.450187 | 2857.453512 | 3092.17213 | 3793.318875 |
| LOC410663 | 35.01087113 | 48.35711034 | 57.75953942 | 53.22576691 |
| LOC410023 | 14.03584701 | 17.83349925 | 19.63137722 | 18.58028788 |
| LOC408354 | 5.364102475 | 9.736279767 | 9.836252932 | 9.031114005 |
| LOC726903 | 2.02168138 | 2.951591619 | 3.705508557 | 3.389955983 |
| LOC411474 | 3.102738213 | 2.531243505 | 2.265776629 | 2.420132986 |
| LOC410045 | 10.57898159 | 9.910892786 | 6.867997992 | 8.475107038 |
| LOC409784 | 63.4857302 | 49.19823476 | 44.97504875 | 45.78024497 |
| - | 1.260365568 | 1.797512474 | 2.078207031 | 2.122481566 |
| LOC724508 | 5.709900627 | 4.613860667 | 3.699195479 | 4.234878033 |
| LOC727260 | 2.934976311 | 2.137349699 | 2.011378841 | 2.070078805 |
| LOC411832 | 932.6669752 | 1198.140168 | 1366.276892 | 1213.716315 |
| LOC413755 | 5.194811556 | 8.142844619 | 9.828435076 | 8.039887354 |
| LOC411255 | 29.13824102 | 38.61509147 | 50.07845708 | 40.51430373 |
| LOC411905 | 12.3076428 | 11.4120326 | 9.888318186 | 10.10704893 |
| LOC552272 | 974.2548522 | 1463.173381 | 1752.593837 | 1511.415014 |
| LOC102655737 | 1.175137835 | 1.566411399 | 1.662950136 | 1.728100418 |
| LOC100578161 | 9.895490528 | 7.837345478 | 7.171102654 | 8.040496566 |
| LOC409548 | 13.39885723 | 10.74222166 | 8.697338985 | 10.40889186 |
| LOC411088 | 14.599057 | 19.15531726 | 24.380239 | 19.07720762 |
| LOC100578467 | 4.146398799 | 4.493711909 | 5.599052629 | 5.769679784 |
| LOC726779 | 7.69907504 | 8.208944763 | 10.9402274 | 11.51392756 |
| LOC552498 | 3.498485661 | 2.848674776 | 2.568713929 | 2.436771093 |
| LOC725884 | 1301.749022 | 1856.371218 | 2367.028207 | 2134.8948 |
| LOC107963970 | 65.75619798 | 111.6702748 | 97.27735969 | 91.78863434 |
| LOC100576859 | 2.242190993 | 2.028825511 | 1.96251862 | 1.447984579 |
| LOC107965022 | 1.67292985 | 1.147084273 | 1.263145214 | 1.128839327 |
| LOC113218987 | 5.926700117 | 3.696582935 | 3.737336654 | 4.34942169 |
| LOC552555 | 28.25917899 | 32.90559067 | 39.08877555 | 36.24948277 |
| - | 0.496374923 | 0.861253756 | 0.617942876 | 0.858567609 |
| LOC551885 | 14.7858866 | 10.62679206 | 9.637626216 | 10.86215611 |
| LOC102653957 | 1.59256832 | 1.303273448 | 0.667529654 | 0.877575093 |
| LOC411889 | 14.83396079 | 23.00375948 | 26.42390426 | 21.47143084 |
| Hex110 | 1.907689173 | 5.367464473 | 2.325257073 | 3.917177015 |
| LOC551451 | 20.92812848 | 15.83087815 | 14.16016715 | 16.00538079 |
| LOC102656491 | 56.31687755 | 78.06175799 | 108.3146504 | 94.21450697 |
| LOC724993 | 29.39110523 | 93.9659965 | 44.68776766 | 41.93053835 |
| LOC551867 | 446.973346 | 630.9642753 | 789.4794172 | 681.9939786 |
| LOC100577727 | 16.11060946 | 18.57220072 | 20.51527453 | 23.20945788 |
| LOC409450 | 303.2987301 | 469.4162914 | 553.1793536 | 464.4988155 |
| LOC409474 | 12.39633811 | 10.44736482 | 8.796715905 | 9.819203564 |
| LOC410804 | 4.488598554 | 4.295121506 | 3.851100381 | 3.570145564 |
| LOC725687 | 3.846263026 | 2.388020734 | 2.301298507 | 2.517602856 |
| LOC552144 | 10.2843619 | 9.304580782 | 9.391056913 | 8.189633208 |
| LOC550711 | 416.2236505 | 742.9860608 | 920.5779691 | 660.4110827 |
| LOC100577801 | 1.324859212 | 1.463426039 | 2.005210369 | 1.852226259 |
| LOC100578597 | 7.579315828 | 6.335276995 | 5.26322291 | 6.095624821 |
| LOC100578537 | 4.528834465 | 3.502129729 | 2.765213534 | 3.068432051 |
| LOC410761 | 0.070722885 | 0.19878789 | 0.299667786 | 0.202298077 |
| LOC102654169 | 170.3276006 | 172.2763007 | 211.6541905 | 214.1224006 |
| LOC724706 | 6.391484224 | 3.267030099 | 3.102989678 | 4.041712913 |
| LOC409924 | 23.62426089 | 30.6917399 | 33.42807321 | 31.67338241 |
| LOC726905 | 0.505581209 | 1.148158876 | 1.161789796 | 0.91634069 |
| LOC409530 | 4.535342237 | 3.601551709 | 2.730730292 | 2.969640127 |
| LOC410546 | 3.353927229 | 3.394895492 | 2.207131004 | 2.435612627 |
| LOC100577002 | 34.27145844 | 58.48788552 | 63.55631853 | 48.07600926 |
| LOC413484 | 26.55635285 | 20.78860731 | 20.09891799 | 20.83301128 |
| LOC726289 | 2.502305191 | 4.075257849 | 3.953943245 | 3.424114317 |
| LOC409406 | 67.49936203 | 76.94029233 | 107.811771 | 88.30120094 |
| LOC551206 | 15.91607449 | 19.26777594 | 23.37726526 | 21.14548754 |
| LOC113218639 | 8.221274215 | 5.746289974 | 6.055805369 | 6.085590377 |
| LOC410332 | 12.32613601 | 14.38485213 | 14.00971992 | 16.75304508 |
| LOC725603 | 2.048499992 | 2.692847449 | 2.613872619 | 3.020310663 |
| LOC411011 | 4.076432503 | 5.576036956 | 4.694614406 | 5.615826617 |
| LOC552105 | 21.44950691 | 21.68163763 | 24.51302407 | 27.20267384 |
| LOC102655124 | 1.894300916 | 2.987160604 | 2.920950835 | 3.012904796 |
| LOC412613 | 8.432211288 | 6.994476365 | 7.391154093 | 6.557726386 |
| LOC412331 | 3.708065901 | 3.394275661 | 2.864008526 | 2.953327285 |
| - | 2.125030816 | 2.630940933 | 3.22090192 | 3.584407943 |
| LOC408920 | 21.10774812 | 24.0705454 | 24.15006173 | 26.88403849 |
| LOC107964020 | 16.65828594 | 19.88995515 | 23.71029637 | 23.28760248 |
| LOC725102 | 27.98833444 | 30.1060265 | 43.56333304 | 41.28294702 |
| LOC726950 | 0.122160655 | 0.473742099 | 0.549575574 | 0.607524314 |
| - | 0.410837385 | 0.194316514 | 0.075845251 | 0.150037139 |
| LOC725756 | 31.80798731 | 46.56716208 | 64.21895952 | 45.1799694 |
| LOC726232 | 6.223048724 | 7.001524673 | 8.204108321 | 9.27060504 |
| LOC412602 | 375.1817184 | 657.2838432 | 869.3391713 | 651.9935719 |
| LOC102655272 | 32.70608538 | 42.58294318 | 72.8889488 | 52.80650372 |
| LOC100578370 | 4.54706555 | 3.448941426 | 3.654213441 | 3.343720453 |
| LOC410887 | 7.095694672 | 7.013016401 | 5.922459842 | 5.882463696 |
| LOC725453 | 88.13810755 | 69.07577465 | 51.49232611 | 61.0697138 |
| LOC408304 | 40.09981858 | 40.83053237 | 66.9325992 | 53.54193682 |
| LOC409891 | 5.656845652 | 4.015477867 | 3.577385129 | 4.094971077 |
| LOC408979 | 57.06052813 | 75.60713486 | 87.31121214 | 80.04234098 |
| LOC726184 | 22.69129956 | 34.87804233 | 45.26726911 | 35.5869196 |
| LOC725918 | 46.61038485 | 54.31125354 | 73.1114673 | 59.99407405 |
| LOC551897 | 3.188027561 | 1.819691457 | 1.715055753 | 1.924413935 |
| LOC409882 | 22.08719219 | 20.09561124 | 15.49846897 | 18.21038582 |
| LOC408826 | 7.715963015 | 15.40091732 | 16.5473314 | 13.27082109 |
| LOC413371 | 3.181201263 | 3.067750486 | 3.704681419 | 4.264898506 |
| LOC409723 | 50.09325391 | 52.5566811 | 72.50133532 | 64.51959975 |
| LOC102654165 | 6.083332829 | 9.376989393 | 9.90444316 | 9.470162931 |
| LOC551042 | 116.1533076 | 120.4532069 | 149.6896739 | 146.9799304 |
| LOC551946 | 32.11796934 | 23.23427028 | 21.10945213 | 24.87788669 |
| LOC551823 | 7.932047219 | 5.869962188 | 5.462646071 | 5.716799896 |
| LOC725938 | 19.90775393 | 16.78721278 | 13.40218721 | 15.72131366 |
| LOC411981 | 4.66853639 | 3.691737638 | 2.731152139 | 3.577369679 |
| LOC408668 | 11.23244748 | 8.748912883 | 8.453993309 | 8.751696988 |
| LOC412944 | 4.237454415 | 4.113944448 | 3.367265183 | 3.397181273 |
| LOC410031 | 7.717673165 | 7.511534897 | 5.117321178 | 6.333322769 |
| LOC413732 | 4.69972798 | 3.888215689 | 3.945926944 | 3.411520779 |
| LOC410255 | 3.851005909 | 3.262941429 | 3.532844888 | 2.993502075 |
| LOC725904 | 19.1444974 | 23.37406951 | 28.19122332 | 26.25532358 |
| LOC550715 | 299.1714114 | 466.4557036 | 567.4667656 | 493.3927274 |
| LOC412543 | 125.9034395 | 158.862268 | 174.1121494 | 164.4159264 |
| LOC551463 | 25.55708942 | 21.51977829 | 22.21056793 | 20.18690494 |
| LOC551158 | 44.25739109 | 49.44180125 | 64.44249037 | 57.41813917 |
| LOC102654791 | 5.632045082 | 4.140579467 | 3.768980904 | 4.046701399 |
| LOC726953 | 3.260881916 | 2.795108707 | 1.88773671 | 2.002513751 |
| LOC410043 | 29.60408065 | 45.85497129 | 47.42491502 | 39.55558718 |
| LOC413376 | 5.935015809 | 4.414528476 | 3.444424112 | 4.209872097 |
| LOC100578006 | 38.98882427 | 45.90745886 | 63.32692689 | 50.89433116 |
| LOC100576097 | 3.29716472 | 2.167901239 | 2.601953445 | 2.285031734 |
| LOC410986 | 23.78158232 | 31.21145311 | 36.39236287 | 32.06117833 |
| LOC410601 | 1.25104066 | 0.740347307 | 0.725924365 | 0.792262214 |
| LOC411727 | 3.876442224 | 2.948624449 | 2.764443506 | 2.950258811 |
| LOC100576826 | 20.57501808 | 35.77871864 | 40.90441755 | 33.1656442 |
| LOC410612 | 20.01266564 | 26.25792018 | 28.21611885 | 26.377811 |
| LOC727223 | 6.058810619 | 5.073059704 | 4.517334758 | 4.982622147 |
| LOC100577988 | 3.927624578 | 2.492809319 | 2.639033803 | 2.609627492 |
| LOC725717 | 2.486194786 | 2.685409787 | 3.341286331 | 3.598473875 |
| LOC410553 | 0.847132242 | 1.059729551 | 1.678816335 | 1.725829162 |
| LOC551521 | 6.169100856 | 4.526717388 | 3.785984767 | 4.537380532 |
| LOC412451 | 12.67189846 | 10.37290801 | 9.055174876 | 9.86061772 |
| - | 11.79430039 | 12.37701612 | 16.78262414 | 16.27139539 |
| LOC409285 | 15.56271117 | 13.97069652 | 13.08763075 | 12.14306896 |
| LOC412506 | 22.04127473 | 20.5209681 | 17.40242591 | 18.12994745 |
| otd2 | 3.919326912 | 4.166346674 | 4.913689853 | 5.274045712 |
| LOC552501 | 24.17168526 | 27.894176 | 35.11803737 | 32.56578282 |
| LOC410128 | 46.19585718 | 48.22701878 | 58.4306842 | 57.46407416 |
| LOC409752 | 16.64521675 | 15.00707966 | 11.83061616 | 12.41064025 |
| LOC100578532 | 1.585241198 | 2.112420861 | 2.03543709 | 2.833773231 |
| LOC102655244 | 3.323585707 | 2.727191734 | 2.659568295 | 2.660161562 |
| LOC409595 | 69.72348775 | 100.5142573 | 133.0408047 | 104.6504591 |
| LOC725949 | 11.48837559 | 9.079434971 | 7.299393462 | 8.561067701 |
| LOC412198 | 10.92274839 | 9.890528421 | 9.432098788 | 8.589833979 |
| LOC412617 | 12.27510161 | 12.14072737 | 8.490304232 | 9.082372153 |
| LOC550884 | 5.918365934 | 5.226301195 | 5.573555766 | 4.456454216 |
| LOC409038 | 7.010920648 | 4.977518083 | 4.226893153 | 5.193514733 |
| LOC725366 | 0.954575823 | 0.770822054 | 0.646679316 | 0.591468172 |
| GlnS | 119.6970005 | 248.6111157 | 220.4840988 | 179.7793977 |
| LOC410211 | 67.68925003 | 73.04108167 | 82.21067089 | 84.24280611 |
| LOC725238 | 15.56304383 | 18.32002775 | 23.25950986 | 21.9292962 |
| LOC409796 | 102.7648536 | 133.396859 | 167.6415848 | 142.9265606 |
| LOC100576143 | 2.117381886 | 2.108030926 | 3.119572604 | 2.967725856 |
| Cpap3-c | 152.0931207 | 174.584522 | 213.6950902 | 223.6005199 |
| LOC410385 | 23.53492308 | 28.6990658 | 34.51836205 | 29.58026981 |
| LOC551330 | 520.7189696 | 966.6592498 | 1203.541217 | 894.1863609 |
| LOC414048 | 4.061443887 | 2.481631123 | 3.013419842 | 2.807587205 |
| LOC413418 | 2.185672463 | 1.388673965 | 1.187664178 | 1.42570912 |
| LOC414029 | 12.08548853 | 10.85912794 | 8.255633962 | 9.416473136 |
| RpL32 | 381.1435634 | 523.5747599 | 616.8285211 | 563.2566719 |
| LOC726478 | 3.29240382 | 3.701218658 | 4.762963085 | 4.48581743 |
| LOC551722 | 9.552547014 | 7.832574145 | 7.211233662 | 7.511456735 |
| LOC724294 | 8.449193993 | 5.79372278 | 5.467031724 | 6.286258289 |
| LOC412454 | 12.40033058 | 7.797527862 | 7.180856568 | 8.780766863 |
| - | 0.298226464 | 0.708360535 | 0.485476507 | 0.668946632 |
| LOC100577120 | 1.769162943 | 1.167654644 | 1.204110463 | 1.204595019 |
| LOC725482 | 0.560263653 | 0.290180279 | 0.143967548 | 0.284158551 |
| LOC552571 | 22.85157205 | 33.85988409 | 36.84280031 | 31.87508327 |
| - | 0.406267982 | 0.187860991 | 0.196127337 | 0.18587422 |
| LOC726900 | 19.00322944 | 26.26761768 | 30.43681398 | 27.29405408 |
| LOC411038 | 3.180353962 | 3.161842143 | 4.160523789 | 4.311796291 |
| LOC410003 | 13.75896846 | 11.70613455 | 9.936316202 | 11.75417636 |
| LOC412549 | 29.69291781 | 32.7892954 | 36.40463549 | 38.12640952 |
| LOC412431 | 10.54347095 | 11.18582312 | 13.17698772 | 13.99569108 |
| LOC552657 | 5.123244637 | 4.495660145 | 4.527639903 | 4.110443019 |
| LOC113218664 | 6.554713401 | 5.734162699 | 5.260292895 | 5.23101371 |
| - | 0.384217526 | 0.471393968 | 0.732185863 | 0.673646947 |
| Reck | 10.64846146 | 15.40185977 | 14.3152332 | 14.10110073 |
| LOC552471 | 21.77534986 | 29.7648392 | 36.8565922 | 31.48212634 |
| LOC724914 | 0.353737944 | 0.470067554 | 0.446718568 | 0.628885192 |
| LOC725061 | 4.920133575 | 3.978889261 | 3.930306572 | 4.012559903 |
| LOC726107 | 13.72542674 | 12.23062666 | 9.836237051 | 10.69641328 |
| LOC551960 | 35.62582658 | 52.62523909 | 63.80572872 | 52.59920855 |
| LOC410486 | 549.7791403 | 882.0942018 | 1058.919402 | 858.2108015 |
| LOC411348 | 17.53072059 | 16.40558386 | 16.09731392 | 14.5397258 |
| LOC100576747 | 1.506434427 | 1.110181487 | 1.138378884 | 1.126222076 |
| LOC724762 | 3.358332509 | 2.064241461 | 2.436057395 | 2.218470168 |
| LOC725405 | 2.16648957 | 1.460196954 | 1.682370059 | 1.65328361 |
| LOC411327 | 0.906211258 | 0.574688328 | 0.746370346 | 0.596338124 |
| LOC551884 | 28.73060437 | 23.47552244 | 21.45219657 | 22.24539034 |
| LOC102653640 | 2.967341441 | 1.861914238 | 1.944064816 | 2.050611503 |
| LOC724487 | 42.64454984 | 45.73111341 | 62.1818744 | 54.71041466 |
| LOC100576482 | 2.442765553 | 1.705031375 | 1.453942248 | 1.801037913 |
| LOC551796 | 23.61227717 | 26.72025284 | 29.22742106 | 31.72489785 |
| LOC551114 | 16.00386502 | 20.81684234 | 26.51998053 | 22.02789034 |
| LOC102655661 | 0.783216436 | 1.954504848 | 1.420739132 | 1.443586103 |
| LOC552173 | 2.752676881 | 2.378670239 | 1.638191966 | 2.082793262 |
| LOC412455 | 9.001970141 | 6.226504582 | 6.393362725 | 6.330643257 |
| LOC100578420 | 16.45071821 | 19.90246973 | 20.29812792 | 24.03052216 |
| LOC413720 | 5.233066472 | 3.930560298 | 4.945955239 | 4.157117129 |
| LOC727349 | 14.75995607 | 14.76702805 | 17.82100975 | 18.84579867 |
| LOC410748 | 1.066968591 | 0.545106969 | 0.595364311 | 0.745452806 |
| LOC726009 | 27.45745724 | 27.85341061 | 38.8058256 | 35.56613089 |
| LOC409778 | 8.094020527 | 8.482326033 | 11.18631931 | 11.47245057 |
| LOC551962 | 28.15841255 | 40.7810747 | 47.84130253 | 38.88088822 |
| LOC552275 | 6.284449913 | 5.692668463 | 4.057187668 | 4.730798136 |
| LOC726778 | 5.00943345 | 4.278986462 | 3.987301167 | 3.865171921 |
| LOC725363 | 9.116699139 | 12.33856709 | 16.4121298 | 13.33507831 |
| LOC100188940 | 21.04998308 | 26.80814121 | 28.40551891 | 30.15960022 |
| LOC100578356 | 1.707380962 | 1.32035313 | 1.056626918 | 1.15914814 |
| LOC725358 | 30.26911282 | 36.73285778 | 46.94925622 | 42.00806041 |
| LOC725908 | 1.436288559 | 1.045462449 | 0.902948313 | 1.018730746 |
| LOC408752 | 12.21275255 | 7.895138295 | 8.049862063 | 9.381588995 |
| Prpf3 | 3.701170126 | 3.494286968 | 2.614208967 | 2.786885888 |
| LOC724322 | 3.482778082 | 4.383011955 | 5.09851745 | 5.108283866 |
| LOC408322 | 26.66437595 | 19.33097712 | 14.82977667 | 19.90962161 |
| LOC551297 | 10.22216125 | 9.796374923 | 8.021010246 | 7.792470478 |
| LOC409403 | 9.058955404 | 8.551601249 | 7.647179329 | 7.350915951 |
| LOC724563 | 13.01606362 | 15.68248497 | 16.39819097 | 17.09573106 |
| LOC724622 | 1.372280084 | 1.612949794 | 2.209947017 | 1.946296192 |
| Fem | 26.85379598 | 20.49791893 | 18.7297588 | 20.20232783 |
| LOC551232 | 0.699364113 | 0.971821887 | 0.86445975 | 1.073362261 |
| LOC409865 | 17.96759474 | 18.00621501 | 13.78735525 | 14.84188683 |
| LOC409735 | 12.30718138 | 12.31231589 | 8.87908595 | 9.901932983 |
| LOC725837 | 4.837047201 | 3.558223982 | 3.979528086 | 3.810966189 |
| LOC410851 | 3.119159219 | 4.660085951 | 5.102606342 | 4.831044305 |
| LOC100576348 | 8.671123435 | 9.66481059 | 11.35537757 | 11.21061867 |
| LOC102656136 | 35.01073676 | 36.88440836 | 46.54555322 | 45.44045606 |
| LOC409476 | 5.459631448 | 4.849675366 | 4.923327556 | 4.131571379 |
| LOC411172 | 7.65908651 | 6.318398897 | 6.220668501 | 6.098455029 |
| LOC100577912 | 13.50704078 | 14.67250045 | 15.70990598 | 18.38091883 |
| LOC410674 | 1.799496167 | 3.01899579 | 2.942488828 | 2.805033237 |
| LOC100577493 | 5.696716396 | 4.350819958 | 3.745556521 | 4.419330571 |
| LOC102654353 | 8.359479287 | 10.74754934 | 16.36544288 | 13.01781251 |
| LOC551644 | 499.6763636 | 694.1027137 | 816.5421233 | 755.2326595 |
| LOC100578388 | 6.879568334 | 5.01613878 | 4.110905416 | 5.083440477 |
| LOC552266 | 466.5462937 | 728.6066794 | 874.107637 | 750.5612627 |
| LOC410567 | 9.62210156 | 13.15245695 | 16.52037344 | 13.85811498 |
| LOC100576745 | 24.10794113 | 27.66022756 | 33.66806718 | 33.87704068 |
| LOC725621 | 40.86686536 | 64.1097095 | 55.00964743 | 59.23232662 |
| LOC552774 | 310.1025966 | 424.3463867 | 548.3349135 | 441.7430083 |
| LOC724531 | 455.0136949 | 645.0298448 | 836.359819 | 681.9056935 |
| LOC409129 | 28.94993668 | 34.23456162 | 37.04525401 | 37.62929619 |
| LOC410106 | 7.699142142 | 6.997209804 | 5.761902699 | 6.083543367 |
| LOC552424 | 100.8346096 | 110.1585275 | 137.6190476 | 127.085326 |
| ND1 | 280.6374762 | 435.3804176 | 337.145242 | 480.9572799 |
| LOC408412 | 72.92392738 | 82.05215939 | 113.9287637 | 96.18593883 |
| LOC724505 | 3.766293631 | 3.787533238 | 3.111682604 | 3.05907652 |
| LOC552075 | 54.89992884 | 73.40743353 | 75.9701217 | 71.34122343 |
| LOC410061 | 16.27241471 | 15.41379182 | 11.64931616 | 13.92612189 |
| LOC724695 | 9.672318439 | 7.333147109 | 6.647743373 | 7.376221383 |
| LOC409912 | 18.67386885 | 14.91136337 | 11.73531532 | 14.18362948 |
| LOC551709 | 2.23752547 | 1.913750433 | 1.649704636 | 1.500685956 |
| LOC726405 | 5.042150216 | 4.588737253 | 4.034702321 | 3.910495877 |
| LOC551167 | 31.60438767 | 37.13985082 | 42.64285437 | 40.97599967 |
| LOC552429 | 85.57870974 | 86.38314584 | 103.7775836 | 107.1833235 |
| Y-h | 1.878026433 | 1.607211434 | 1.907820505 | 1.134573275 |
| LOC725886 | 3.896989317 | 3.239987933 | 3.043504266 | 2.940596669 |
| LOC410987 | 9.838566827 | 8.558208041 | 6.845358743 | 8.068354597 |
| LOC411715 | 10.88835102 | 9.27630023 | 8.661464603 | 8.893874501 |
| LOC410885 | 1.41199423 | 1.280219185 | 0.697138175 | 0.945859998 |
| LOC552108 | 3.228102047 | 2.322632964 | 2.091946828 | 2.150359285 |
| LOC726325 | 7.127828856 | 5.597443196 | 4.982257671 | 5.73487917 |
| LOC409347 | 6.607087308 | 4.91280304 | 4.582523208 | 4.9135927 |
| LOC411922 | 11.68333822 | 8.210658933 | 7.91427241 | 8.434161275 |
| LOC412770 | 1.543619349 | 1.728246268 | 2.262142461 | 2.170364862 |
| nAChRa9 | 1.762427465 | 2.752703484 | 3.545618706 | 3.776780623 |
| LOC410441 | 12.80541465 | 22.63227906 | 27.53752844 | 19.42426842 |
| LOC413113 | 10.44074832 | 11.24826067 | 13.60186839 | 14.12939247 |
| LOC725665 | 18.98348714 | 23.2409763 | 27.51137477 | 25.05003173 |
| LOC724607 | 0.545470104 | 1.212842935 | 1.0362032 | 1.025382302 |
| LOC725156 | 11.01716008 | 9.414587807 | 7.419272983 | 8.227162714 |
| LOC551606 | 16.63319937 | 20.4434098 | 26.60839953 | 23.13618311 |
| LOC552680 | 8.719156091 | 7.096199417 | 6.848985265 | 6.452889458 |
| LOC552782 | 4.001297005 | 3.238929987 | 3.120213845 | 3.196156337 |
| LOC552070 | 2.717381131 | 2.288203344 | 1.842598938 | 1.986359406 |
| LOC411891 | 6.245211245 | 6.060767537 | 4.673971837 | 4.947846859 |
| LOC552074 | 12.70298738 | 12.35245354 | 9.826486036 | 10.81669182 |
| LOC410234 | 7.48288971 | 6.260528474 | 6.14150331 | 6.078041721 |
| LOC408359 | 17.2990514 | 20.73063203 | 27.91164314 | 22.91680774 |
| LOC411607 | 37.39553476 | 50.49617242 | 69.00977178 | 56.04547518 |
| LOC725019 | 103.0521712 | 78.38524184 | 105.3887119 | 80.16190668 |
| LOC409408 | 6.407350143 | 5.398056657 | 5.296572333 | 4.907233223 |
| LOC725294 | 2.914915638 | 5.244336964 | 4.267552784 | 4.071583938 |
| LOC411721 | 7.212164923 | 4.121454056 | 4.131057846 | 5.042625354 |
| LOC100576864 | 1.910699836 | 1.126022716 | 1.163282648 | 1.281161861 |
| LOC724431 | 3.696918773 | 3.379479907 | 3.134791301 | 2.895487836 |
| LOC410307 | 4.756561527 | 4.392841139 | 3.554521776 | 3.841674422 |
| LOC100578715 | 3.492630062 | 3.232260371 | 3.374404443 | 2.475998776 |
| LOC725154 | 1.575317878 | 5.450908459 | 3.811941089 | 3.198670336 |
| LOC725530 | 87.24955805 | 112.6412577 | 136.4166586 | 124.9313856 |
| LOC413204 | 3.331014429 | 2.076073179 | 2.660712204 | 2.301426126 |
| LOC100576968 | 6.546129325 | 6.108837484 | 5.085795972 | 4.944277599 |
| LOC413407 | 2.276659373 | 1.813140593 | 1.869526213 | 1.720702457 |
| LOC411047 | 7.640641468 | 7.230557269 | 6.099226464 | 6.049702459 |
| LOC551888 | 4.913542827 | 4.32674845 | 4.091312312 | 3.969443697 |
| LOC409294 | 632.4480855 | 1002.100868 | 1239.77567 | 1010.059481 |
| LOC411059 | 2.538423128 | 1.659930323 | 1.641712433 | 1.652214504 |
| LOC413207 | 23.36907973 | 45.49827136 | 35.10204263 | 31.46132273 |
| LOC413976 | 11.97848357 | 17.81478678 | 20.04673815 | 16.33197119 |
| LOC102655030 | 28.22973465 | 38.63061893 | 37.46454659 | 36.36932088 |
| LOC410684 | 0.304458517 | 0.343042225 | 0.419369646 | 0.522324362 |
| LOC727222 | 5.862561925 | 4.884328832 | 5.114700232 | 4.637127857 |
| LOC551225 | 16.24956089 | 14.76539112 | 12.61653826 | 13.03215961 |
| LOC410122 | 513.272802 | 714.5066359 | 732.8500304 | 645.9565153 |
| LOC408712 | 7.931328259 | 4.782071088 | 4.4097398 | 5.533798532 |
| LOC406147 | 97.44884107 | 86.22439132 | 91.67285213 | 74.26155079 |
| LOC412584 | 4.080909232 | 3.582474793 | 3.823611513 | 3.099623132 |
| LOC413450 | 15.46597921 | 12.9049324 | 13.64508261 | 12.39087103 |
| LOC724156 | 0.115545357 | 0.211756545 | 0.286065983 | 0.246401595 |
| LOC411495 | 3.300113753 | 2.235095319 | 1.761268618 | 2.13786183 |
| LOC100579053 | 12.61292139 | 9.518894572 | 9.439757192 | 7.38672439 |
| LOC413844 | 5.451982212 | 6.688613452 | 8.708557394 | 7.655067424 |
| LOC100577590 | 0.331050796 | 0.213486669 | 0.126582496 | 0.134869902 |
| LOC725395 | 11.36916074 | 9.432168353 | 9.651599813 | 9.04632044 |
| LOC412711 | 4.276666838 | 3.557905163 | 3.100760716 | 3.081471013 |
| LOC552268 | 20.73933903 | 26.58332366 | 32.60584467 | 29.23228473 |
| LOC727331 | 14.15519558 | 12.07217582 | 10.30241471 | 11.67643058 |
| LOC412700 | 13.17134612 | 10.60518511 | 9.677829999 | 10.86940046 |
| LOC413668 | 2.749378005 | 2.411560868 | 1.473812124 | 2.06515938 |
| LOC408691 | 48.91002704 | 55.99999918 | 68.77367229 | 61.49097576 |
| LOC552318 | 701.5528133 | 1066.876128 | 1323.544492 | 1084.009767 |
| LOC551841 | 11.79796601 | 15.50923534 | 17.73656574 | 16.17579837 |
| LOC724192 | 17.57772715 | 16.35765035 | 12.0297166 | 13.61035065 |
| LOC410505 | 23.47111619 | 13.50266704 | 16.34127341 | 16.8898746 |
| LOC408542 | 11.34720611 | 7.611590111 | 6.435755864 | 8.096618209 |
| LOC409673 | 0.606183647 | 0.446113887 | 0.535148281 | 0.428626265 |
| LOC100578773 | 6.094207656 | 4.225684959 | 4.001500936 | 4.412789591 |
| LOC113218958 | 112.0148509 | 117.4513076 | 125.4056187 | 135.0012033 |
| LOC726148 | 1.657102519 | 1.694425547 | 2.131943321 | 2.249717658 |
| LOC410274 | 6.334518699 | 6.246732183 | 4.96436287 | 5.17236499 |
| LOC551590 | 12.20927039 | 8.596033934 | 7.736151015 | 9.386470061 |
| - | 0.115998252 | 0.139056832 | 0.187224739 | 0.500536207 |
| LOC107963990 | 3.112855289 | 2.371614322 | 2.17206706 | 2.53412119 |
| LOC412018 | 10.64807152 | 8.27204678 | 6.662838546 | 8.030139224 |
| LOC410424 | 15.42463172 | 18.09496382 | 18.91729256 | 19.61030197 |
| LOC410286 | 46.96908988 | 36.79816751 | 33.25215465 | 37.64108091 |
| LOC724142 | 225.7056363 | 289.2606191 | 384.935245 | 321.747433 |
| LOC408911 | 9.166006487 | 8.100932263 | 7.318858408 | 7.449409324 |
| LOC724613 | 3.156874151 | 3.97351993 | 4.676993152 | 4.327038751 |
| LOC726164 | 50.97779619 | 61.53165674 | 69.51418898 | 66.89605477 |
| LOC412326 | 5.455388843 | 5.331089773 | 4.850024971 | 4.361689076 |
| LOC726718 | 4.255609576 | 5.858915983 | 6.862035309 | 6.57395611 |
| CSP1 | 382.7288661 | 619.1572721 | 681.7648513 | 613.0269942 |
| LOC408407 | 46.81040331 | 51.79379347 | 66.9934962 | 59.65475788 |
| LOC551695 | 25.40846226 | 26.80299446 | 34.77416415 | 32.15793422 |
| LOC409586 | 98.90283729 | 109.7581536 | 127.8992353 | 125.6999228 |
| LOC411679 | 8.478580219 | 8.676899386 | 6.921570803 | 6.971605964 |
| LOC551708 | 3.51112764 | 2.964048059 | 2.53388606 | 2.688374643 |
| LOC725051 | 127.4684753 | 62.20342186 | 73.68188263 | 90.85445798 |
| LOC726498 | 37.93936352 | 44.55417483 | 59.23998867 | 53.91826935 |
| LOC102655890 | 0.982564086 | 0.770838984 | 0.380157718 | 0.373053651 |
| LOC726724 | 3.654020398 | 3.804945727 | 5.250583693 | 4.974104758 |
| LOC410499 | 2.307748928 | 1.631385912 | 1.457215167 | 1.53583424 |
| LOC725618 | 4.85786744 | 3.243151824 | 3.813379919 | 3.586934911 |
| LOC410626 | 4.907826057 | 20.75228436 | 14.31701833 | 12.98604426 |
| LOC408405 | 1.554174632 | 1.728740494 | 2.340993719 | 2.250404374 |
| LOC100578260 | 7.112389366 | 6.075250178 | 5.141700566 | 5.486106134 |
| LOC412154 | 26.09127919 | 22.07157179 | 21.73267645 | 20.70799578 |
| Obp11 | 12.22014819 | 8.227888679 | 11.19501744 | 8.108634486 |
| LOC411105 | 8.604129439 | 6.465215503 | 5.537395538 | 6.313505545 |
| LOC412880 | 11.80404031 | 9.759492887 | 9.047967128 | 8.857652038 |
| LOC726171 | 684.5126639 | 1064.66784 | 1319.690654 | 1051.782642 |
| LOC551106 | 52.76826919 | 76.51749957 | 89.25311216 | 80.80668784 |
| LOC724466 | 1.070841071 | 1.906673947 | 1.68744519 | 1.701387704 |
| LOC550734 | 43.88629746 | 55.15520702 | 65.13277243 | 53.64561367 |
| LOC409804 | 33.51496314 | 33.02330673 | 22.1633864 | 29.10530222 |
| LOC551766 | 715.8690416 | 944.3797724 | 1126.715094 | 924.2162756 |
| - | 0.203754299 | 0.092039989 | 0.071360382 | 0.072779836 |
| LOC409614 | 12.89447462 | 18.31038165 | 24.47032798 | 18.74748512 |
| LOC410235 | 2.177713755 | 4.681471239 | 7.295182304 | 5.725554434 |
| LOC102654127 | 20.55958211 | 19.48788869 | 15.11840514 | 16.24511896 |
| LOC551044 | 37.96475319 | 243.2375831 | 186.5867202 | 412.375218 |
| LOC551673 | 8.65183144 | 8.820512687 | 7.106276503 | 7.421029965 |
| LOC551539 | 13.66379672 | 13.93290054 | 19.21094645 | 17.66508986 |
| LOC412918 | 13.59642846 | 9.159411635 | 6.981715899 | 9.681757167 |
| LOC551374 | 11.40284 | 8.576455004 | 7.533909825 | 8.96990105 |
| LOC409722 | 13.30802126 | 16.31757685 | 20.54780263 | 17.48963351 |
| LOC724407 | 51.40250435 | 92.39402669 | 133.7079089 | 90.44360067 |
| - | 1.416932476 | 1.178249597 | 0.940527632 | 1.004183886 |
| LOC726042 | 562.6319458 | 579.526116 | 692.4992394 | 704.1856085 |
| LOC725211 | 7.536770927 | 6.750220403 | 7.006968631 | 5.989677507 |
| LOC551830 | 7.012586654 | 6.120587502 | 5.266661939 | 5.405584347 |
| LOC552372 | 49.88369483 | 56.12256854 | 68.89876263 | 62.88032226 |
| LOC552492 | 26.88098222 | 27.85250206 | 32.95377324 | 34.34054365 |
| LOC551586 | 17.38606168 | 17.65710207 | 20.61441898 | 22.2162196 |
| LOC411603 | 14.70235009 | 12.08523209 | 10.33763942 | 11.73170487 |
| LOC725106 | 0.266479103 | 0.196627104 | 0.145265723 | 0.1101813 |
| LOC726295 | 586.9884592 | 863.6498881 | 1038.186983 | 892.9615404 |
| LOC409969 | 8.902620651 | 6.817047262 | 6.7679946 | 6.345997664 |
| LOC551430 | 4.878314031 | 4.825108073 | 3.715347718 | 3.816978905 |
| LOC552835 | 30.03234637 | 30.14196176 | 40.76808094 | 38.79076154 |
| LOC411003 | 6.795107057 | 5.837407253 | 5.16714678 | 5.591237647 |
| LOC100577499 | 1.33742133 | 1.469927218 | 1.856671726 | 2.063529373 |
| LOC725566 | 122.9283265 | 130.251265 | 156.6353747 | 149.5959218 |
| LOC412976 | 0.390431037 | 0.315555614 | 0.24017279 | 0.212485422 |
| LOC552674 | 3.66139658 | 2.235762958 | 2.536910663 | 2.633572754 |
| LOC552227 | 115.2450399 | 168.8815798 | 215.7824785 | 190.4594071 |
| LOC408358 | 2.498180112 | 5.707311798 | 4.542215022 | 4.348684889 |
| LOC726650 | 11.32229443 | 7.638064615 | 8.267371886 | 9.423868661 |
| LOC552647 | 8.299173471 | 7.797396891 | 6.282099189 | 6.668732081 |
| LOC552688 | 6.638946307 | 10.58721708 | 12.03307143 | 9.104587646 |
| LOC551146 | 22.09389031 | 17.6570458 | 19.81730479 | 18.8584002 |
| LOC724757 | 56.01791528 | 61.98583335 | 71.17260574 | 69.01530471 |
| LOC410785 | 3.418776313 | 5.250473249 | 5.465708076 | 4.843926858 |
| LOC408292 | 3.908356423 | 6.079738413 | 5.459160881 | 5.808698769 |
| LOC411085 | 16.13845853 | 16.10993635 | 22.23139365 | 20.83692912 |
| LOC100577231 | 0.965998402 | 0.830898617 | 0.540919214 | 0.674653935 |
| LOC100576439 | 2.848664881 | 2.80755558 | 2.620291682 | 1.957196571 |
| LOC726824 | 16.39782924 | 18.83019849 | 20.86011864 | 20.20038114 |
| LOC551599 | 9.265476969 | 6.749067404 | 6.624705639 | 7.019496677 |
| LOC503505 | 1438.632755 | 2102.35019 | 2251.166266 | 1895.285134 |
| LOC725987 | 5.071539785 | 5.491189341 | 5.734359799 | 6.711745315 |
| - | 0.158127441 | 0.776033647 | 0.614327422 | 0.554166874 |
| LOC724698 | 0.326104625 | 0.145524152 | 0.148974478 | 0.115972378 |
| LOC107964039 | 1.103518586 | 1.514304912 | 1.703365886 | 1.870148254 |
| LOC412731 | 7.130209754 | 5.451847054 | 4.33977915 | 5.36021148 |
| LOC724128 | 50.58633421 | 51.26774617 | 62.81496506 | 63.42011216 |
| LOC406081 | 1050.772643 | 3875.129871 | 4008.042127 | 6403.008444 |
| LOC102656890 | 35.44771947 | 47.89102748 | 60.00241443 | 52.76941262 |
| LOC552482 | 11.39184932 | 10.96435201 | 9.238472621 | 8.315482217 |
| LOC410188 | 581.3132169 | 820.5652111 | 995.3235306 | 853.6996128 |
| LOC725390 | 14.03363156 | 14.84809282 | 19.51198949 | 18.12946335 |
| IF-2mt | 14.62395121 | 9.642077349 | 9.280106113 | 11.07333814 |
| LOC724663 | 5.92269465 | 4.133302449 | 3.623582007 | 4.194286278 |
| LOC100576768 | 3.837379649 | 2.528049824 | 2.424066007 | 2.732812037 |
| LOC552097 | 32.9273628 | 40.26766514 | 49.05478355 | 42.08549812 |
| LOC726540 | 3.588136674 | 2.618501384 | 2.434967964 | 2.597299607 |
| LOC409206 | 8.344851312 | 6.976565054 | 5.714175451 | 6.435498105 |
| LOC100577739 | 1.193402232 | 0.869039784 | 0.864759715 | 0.838454232 |
| LOC551443 | 3.246763829 | 2.241962735 | 1.8453934 | 2.258566044 |
| LOC410261 | 1.127724515 | 1.196860178 | 2.054435485 | 1.551682708 |
| LOC552744 | 22.61386754 | 20.68559828 | 18.53766948 | 19.23597527 |
| LOC413145 | 60.83913944 | 88.4249666 | 124.8618572 | 92.28160512 |
| LOC100578769 | 13.69645535 | 19.94777368 | 21.66336943 | 19.03243365 |
| LOC411079 | 1.038516656 | 1.840247727 | 1.840563498 | 1.630124839 |
| LOC726838 | 2.107572693 | 2.45055732 | 3.602599495 | 3.439822663 |
| LOC408314 | 6.41964653 | 4.766989979 | 5.165792326 | 4.98919802 |
| LOC412950 | 7.341092782 | 5.417634908 | 4.554180655 | 5.218258011 |
| LOC411663 | 9.362609772 | 8.221534596 | 7.199910054 | 6.839565804 |
| LOC410386 | 4.864875409 | 5.034611631 | 5.942013445 | 6.145085488 |
| LOC551433 | 2.359427219 | 1.690383499 | 1.11144781 | 1.580060144 |
| LOC552410 | 164.4861711 | 189.9408475 | 224.0995815 | 210.9478311 |
| LOC412197 | 37.96382202 | 60.63528846 | 59.20388888 | 52.96773295 |
| LOC552403 | 58.27046281 | 60.08856196 | 79.20768776 | 74.47209399 |
| LOC725893 | 15.0097986 | 18.23853501 | 18.2545082 | 20.01100435 |
| LOC727648 | 11.11598812 | 10.91380186 | 9.393143389 | 9.092889858 |
| LOC726176 | 52.78843153 | 55.86765514 | 64.72754812 | 66.81782047 |
| LOC552261 | 57.01956541 | 49.24926848 | 36.11292401 | 43.28081623 |
| LOC726706 | 18.72805151 | 21.18668964 | 29.46501263 | 23.49904283 |
| LOC726978 | 65.00026377 | 76.77536707 | 96.30676101 | 92.76394006 |
| LOC410992 | 23.27197834 | 22.91464035 | 29.20022386 | 29.33271834 |
| Obp21 | 203.9049859 | 257.265331 | 300.0926419 | 291.6028152 |
| LOC724585 | 8.808353023 | 14.37333185 | 15.99583053 | 11.97579696 |
| LOC100578853 | 12.67496562 | 13.20253382 | 12.74264778 | 16.15580237 |
| LOC409926 | 48.70578759 | 49.82006691 | 62.17261233 | 60.67332203 |
| LOC102656433 | 13.11824796 | 9.779024244 | 7.986155787 | 10.06402264 |
| LOC411642 | 8.71350643 | 7.372869955 | 7.613609928 | 6.571873134 |
| LOC100576759 | 29.63909737 | 55.38912304 | 70.63610308 | 46.40407147 |
| LOC408429 | 38.4846544 | 48.5339917 | 50.65348137 | 50.79492038 |
| LOC410370 | 16.91318944 | 23.1763095 | 22.55858513 | 23.86314279 |
| LOC552182 | 13.84291599 | 12.12512671 | 13.35942866 | 11.3115185 |
| LOC552354 | 35.95893545 | 47.03787871 | 51.53276153 | 47.93489881 |
| LOC412266 | 328.3712912 | 433.6597098 | 535.7989447 | 465.3819046 |
| LOC408871 | 29.17391625 | 31.57850582 | 43.61770984 | 44.6154414 |
| LOC408730 | 8.674657363 | 8.371578635 | 7.029164771 | 6.698920656 |
| LOC551102 | 32.05205912 | 33.62692595 | 44.51619254 | 39.41020457 |
| LOC411881 | 3.209167975 | 2.888569295 | 2.322502477 | 2.512461376 |
| LOC409068 | 22.35828039 | 32.63025859 | 36.99619493 | 29.65381677 |
| LOC726056 | 438.9729602 | 654.1541795 | 777.7046721 | 661.3485349 |
| LOC724704 | 2.621777722 | 5.239221674 | 5.021915862 | 4.160417099 |
| LOC411065 | 1.331296098 | 1.707634978 | 2.282558551 | 2.05628524 |
| LOC409017 | 4.653159519 | 4.263568223 | 3.77159147 | 3.668275429 |
| LOC412034 | 1.695852264 | 1.402816289 | 1.170648084 | 1.21110548 |
| LOC724383 | 12.19754969 | 9.563797067 | 8.460355906 | 9.757988374 |
| LOC411918 | 4.498684231 | 4.190361777 | 3.711871265 | 3.650097205 |
| LOC102655319 | 1.944244496 | 2.975483451 | 3.229656225 | 3.467396573 |
| LOC552067 | 30.86306261 | 36.38929377 | 44.83110746 | 40.60844413 |
| LOC409315 | 11.90689798 | 11.23405744 | 10.20071885 | 10.03515191 |
| LOC552459 | 370.783242 | 424.4340109 | 392.5977214 | 464.4434691 |
| LOC412629 | 13.16644614 | 11.04096271 | 9.154899406 | 10.60844869 |
| LOC726361 | 4.283662294 | 5.217609359 | 5.92384259 | 5.903488325 |
| LOC100576515 | 36.63294006 | 46.86334173 | 53.62310837 | 53.57665835 |
| LOC411508 | 12.99503094 | 10.14874419 | 9.952310489 | 10.05374782 |
| LOC552604 | 12.86363067 | 11.74420001 | 9.660826955 | 10.54871829 |
| LOC413399 | 8.945908369 | 10.35636254 | 12.66089406 | 12.61723394 |
| Tpi | 118.0600556 | 146.8598883 | 165.2517305 | 149.4027618 |
| Rfwd3 | 14.90924531 | 11.28909301 | 8.724333909 | 11.63138768 |
| LOC726093 | 48.94414227 | 44.79585534 | 42.95017515 | 42.79417932 |
| LOC411046 | 6.381732212 | 9.95701827 | 9.247132609 | 9.181981313 |
| LOC409456 | 4.835749995 | 3.133541323 | 2.735895078 | 3.289925246 |
| LOC725754 | 1.714319664 | 2.580376284 | 2.284045645 | 2.392458984 |
| LOC409270 | 28.58003398 | 22.62646601 | 22.0700269 | 22.64163908 |
| LOC724831 | 14.23106598 | 10.12115341 | 8.841439594 | 10.19489996 |
| LOC411546 | 12.78330599 | 14.8534042 | 17.1194803 | 16.8386571 |
| LOC413690 | 3.225815136 | 2.49717588 | 2.408780528 | 2.638092084 |
| LOC725178 | 4.480682397 | 3.503374636 | 2.713387083 | 3.160447787 |
| LOC724553 | 8.14244981 | 10.41356575 | 12.64146104 | 10.44082117 |
| LOC411503 | 6.548371689 | 6.852611864 | 6.486396187 | 5.260916777 |
| LOC413001 | 14.80166479 | 13.07806675 | 11.50489546 | 12.12044814 |
| LOC724808 | 23.88045476 | 26.27566936 | 32.09889203 | 29.56082811 |
| LOC100578776 | 1.385962388 | 1.739800042 | 1.611686444 | 1.881562587 |
| LOC409564 | 15.76112619 | 12.46507177 | 11.61475867 | 12.79946141 |
| LOC409290 | 716.2903972 | 950.2375207 | 1190.938846 | 948.9459562 |
| LOC409488 | 51.90075802 | 59.71503783 | 59.00222299 | 67.90389868 |
| LOC413947 | 16.43842381 | 12.25228717 | 9.40853514 | 12.1554261 |
| LOC412464 | 2.206020588 | 2.064735192 | 2.062068721 | 1.758474545 |
| LOC550955 | 82.69152717 | 86.75749409 | 116.6321141 | 102.228166 |
| LOC408833 | 33.3519383 | 32.40040154 | 46.04533048 | 41.05437933 |
| LOC409342 | 13.11697376 | 14.78487722 | 17.5430842 | 16.78518606 |
| LOC411124 | 2.373231403 | 3.002474323 | 3.001687525 | 3.043879146 |
| LOC100576096 | 0.278672349 | 0.197601771 | 0.159209486 | 0.1564533 |
| LOC411411 | 131.7088205 | 152.9082374 | 162.0741306 | 157.8791989 |
| LOC100576591 | 1.354071699 | 0.741284468 | 0.616996745 | 0.895871945 |
| LOC724681 | 28.44543681 | 28.75371862 | 40.91495178 | 36.79500664 |
| LOC725057 | 25.44120154 | 40.85012015 | 57.93537597 | 38.75360607 |
| LOC550735 | 1.453875618 | 1.489302305 | 1.128650006 | 1.03830593 |
| LOC409256 | 3.286814058 | 2.838382288 | 2.644000479 | 2.535921705 |
| LOC413213 | 1.75625862 | 2.455852905 | 4.221319139 | 2.827301595 |
| LOC552115 | 150.3741962 | 180.7069168 | 201.7088893 | 184.42697 |
| LOC100379261 | 24.15412408 | 29.16735632 | 25.91072482 | 29.49771675 |
| LOC411423 | 14.0879743 | 15.07562895 | 18.89685078 | 17.36232227 |
| LOC409136 | 6.803016939 | 6.594328409 | 4.15550371 | 5.270332302 |
| LOC410204 | 45.52543394 | 50.94691748 | 57.43566265 | 58.17112297 |
| LOC725241 | 0.435903277 | 0.823461352 | 0.626638287 | 0.677729016 |
| LOC410094 | 19.88819293 | 29.41622645 | 36.97416715 | 28.7907468 |
| LOC413118 | 4.083971698 | 2.708699242 | 2.868224289 | 3.139196681 |
| - | 1.489204239 | 0.89254796 | 0.867844937 | 1.009497251 |
| LOC113218716 | 22.9110982 | 28.71028656 | 37.00180468 | 29.66271639 |
| LOC100578281 | 6.782518455 | 5.736187962 | 5.010737803 | 5.590632165 |
| LOC724569 | 4.894384843 | 5.808967201 | 6.933713421 | 7.063601456 |
| LOC552748 | 4.158229993 | 2.769639967 | 3.663874178 | 2.97386332 |
| LOC413488 | 11.97438079 | 12.05300934 | 8.85185497 | 10.12954901 |
| LOC408301 | 15.44346592 | 18.00805395 | 21.77767375 | 19.21074049 |
| LOC102655463 | 1.033930121 | 1.41958898 | 1.42395939 | 1.665278932 |
| LOC408589 | 85.90940041 | 100.5332378 | 116.5417166 | 108.3453495 |
| LOC726065 | 4.718965905 | 3.551747386 | 2.945754247 | 3.873952899 |
| - | 1.592411845 | 0.692119038 | 1.103354651 | 1.060315471 |
| LOC408503 | 45.37560406 | 54.96134659 | 70.55332285 | 62.85229758 |
| LOC410951 | 3.179027951 | 4.122115676 | 3.970797956 | 4.442596381 |
| LOC413959 | 7.436886242 | 7.111365711 | 6.053198628 | 5.795118332 |
| LOC552597 | 5.77765102 | 6.593720479 | 8.104093259 | 8.068616519 |
| LOC408467 | 2.125700711 | 3.092980097 | 3.083176129 | 2.970120804 |
| LOC726099 | 6.260908866 | 6.19049434 | 9.216236178 | 8.189738802 |
| LOC552686 | 5.309537274 | 7.625577721 | 7.233061103 | 10.59059544 |
| LOC107964012 | 1.229900425 | 1.103700277 | 0.734208328 | 0.431744955 |
| LOC409425 | 12.81818711 | 18.5990868 | 19.93154096 | 17.19941704 |
| LOC726016 | 8.767988074 | 10.33855959 | 12.61707044 | 12.24341661 |
| LOC408321 | 13.59711428 | 11.92151319 | 9.246945691 | 10.93052451 |
| LOC725286 | 7.949827316 | 5.401399171 | 5.125782038 | 5.998402741 |
| LOC100578309 | 1.394427122 | 1.16213837 | 1.076054159 | 0.835332795 |
| LOC552128 | 105.7721379 | 125.510738 | 142.0233316 | 127.9423078 |
| LOC411536 | 6.136767363 | 5.619759464 | 4.635773728 | 5.257399581 |
| LOC409999 | 1.706595271 | 1.884334767 | 2.041378708 | 2.948561919 |
| LOC724895 | 0.217563927 | 0.089483586 | 0.020683628 | 0.054581323 |
| LOC410862 | 2.008473692 | 3.215518258 | 2.4082411 | 2.791623319 |
| LOC107965219 | 0.19203432 | 0.100171829 | 0.079391302 | 0.073917287 |
| LOC410956 | 1.322350667 | 1.612424472 | 1.925717172 | 1.795940298 |
| LOC724670 | 6.43972969 | 4.477442106 | 3.426631753 | 4.792889642 |
| LOC102655090 | 44.18354311 | 39.84032282 | 32.94248953 | 37.1434336 |
| LOC410133 | 4.162811111 | 5.120664208 | 5.544983931 | 5.469725591 |
| LOC551517 | 10.43653704 | 9.772049601 | 9.13905297 | 8.777873157 |
| LOC551911 | 5.465607021 | 4.005202561 | 3.887371725 | 4.178209305 |
| LOC724288 | 3.302485347 | 2.930129468 | 2.503884927 | 2.724701735 |
| CUTA | 16.3673048 | 20.19047019 | 25.05330917 | 20.59422805 |
| LOC100578355 | 3.872252898 | 2.837603211 | 2.830373596 | 2.962909012 |
| LOC100576270 | 20.86724133 | 22.29283892 | 32.97710378 | 27.74734729 |
| LOC408447 | 5.560075343 | 4.405816759 | 2.690375358 | 4.068428983 |
| LOC411538 | 17.95161061 | 21.08699518 | 24.11132226 | 22.10798191 |
| LOC413034 | 21.85064494 | 25.20156052 | 30.85697329 | 26.90376403 |
| LOC409459 | 17.30412832 | 17.16174836 | 14.71842039 | 14.03202364 |
| LOC414015 | 14.01608016 | 12.61006176 | 10.47288425 | 11.74092333 |
| LOC726439 | 673.6150696 | 950.6364152 | 1135.103738 | 959.7424347 |
| LOC551308 | 5.886517889 | 4.777710231 | 4.501168379 | 4.620967582 |
| LOC102655706 | 2.425094235 | 2.655810953 | 4.421597868 | 3.267454672 |
| LOC412281 | 13.05487427 | 11.55031782 | 10.6021775 | 11.2093927 |
| LOC412132 | 3.917900875 | 3.62365637 | 3.097837759 | 3.227200044 |
| LOC552634 | 26.40754722 | 32.90578294 | 43.19867176 | 35.75928876 |
| LOC552549 | 5.744708222 | 5.597687397 | 4.289764661 | 4.682730461 |
| LOC113219112 | 3.634503683 | 4.505274947 | 5.54184313 | 6.319054057 |
| LOC412986 | 10.79426952 | 12.75318215 | 14.97601681 | 13.92372393 |
| LOC408662 | 3.021925482 | 2.471811612 | 2.452482633 | 2.414292299 |
| LOC408462 | 7.987847157 | 9.514869022 | 12.17010588 | 11.37127354 |
| LOC725880 | 61.84625085 | 76.02442504 | 99.48175386 | 80.56653479 |
| LOC107965674 | 23.07060735 | 24.04724772 | 30.60982518 | 30.44872543 |
| LOC413210 | 4.678442425 | 6.412835361 | 6.189029234 | 6.587697471 |
| Rps14 | 597.9202927 | 760.5624835 | 1003.815634 | 862.3254908 |
| LOC552282 | 540.5647473 | 628.8462146 | 762.2552895 | 693.7634686 |
| LOC551355 | 2.963666705 | 2.397600658 | 2.331073941 | 2.354381801 |
| LOC409272 | 17.8327954 | 18.25327744 | 23.08178312 | 22.16267696 |
| LOC725696 | 3.417526757 | 3.012177853 | 2.946592205 | 2.606035481 |
| LOC410345 | 11.22151861 | 9.348792359 | 8.276980362 | 9.21150371 |
| LOC727591 | 4.889555916 | 3.613544451 | 4.213017077 | 3.812607564 |
| LOC409359 | 8.762950715 | 7.672203613 | 6.569735479 | 6.748273736 |
| LOC552185 | 6.220666645 | 4.30176531 | 4.074265478 | 4.620608504 |
| LOC551968 | 169.1989002 | 179.0024422 | 236.5993222 | 213.4576687 |
| LOC552496 | 5.862238645 | 3.821441313 | 4.076319916 | 4.206387093 |
| LOC412923 | 2.45666825 | 2.834495271 | 4.345571308 | 4.069153762 |
| LOC725700 | 19.09614386 | 20.1609812 | 26.02628478 | 23.70370528 |
| LOC412832 | 1.204209287 | 0.77456988 | 0.916723265 | 0.781148199 |
| - | 0.372826585 | 0.796150023 | 0.437026156 | 0.750725874 |
| LOC552671 | 213.9289891 | 266.9533556 | 310.7698509 | 272.6489877 |
| LOC551633 | 11.64503463 | 17.54785197 | 21.4852581 | 15.77534231 |
| LOC100578666 | 1.171917725 | 0.626297148 | 0.284064247 | 0.500079892 |
| LOC410428 | 5.42558622 | 3.605611864 | 3.057510892 | 4.000298829 |
| LOC102655503 | 3.948047876 | 2.912481708 | 3.058376771 | 3.185503601 |
| Argk | 413.5142429 | 489.1191114 | 527.9328566 | 510.8833046 |
| LOC725498 | 1.112418274 | 1.559327573 | 2.125819069 | 1.765824692 |
| LOC408944 | 7.318048415 | 10.75834153 | 10.826132 | 10.37931232 |
| LOC102654271 | 7.996174185 | 5.027388848 | 5.77529031 | 5.85489365 |
| - | 0.66371733 | 0.369136142 | 0.466549754 | 0.277705771 |
| LOC102656027 | 0.570861432 | 1.660574346 | 1.450295882 | 1.29060259 |
| LOC410527 | 13.090968 | 14.20865591 | 17.07687816 | 18.2784857 |
| LOC725556 | 8.103981985 | 6.390803823 | 4.996790483 | 6.054610526 |
| LOC100578404 | 3.706568271 | 2.655556653 | 2.663540281 | 2.989791433 |
| LOC107965532 | 1.032371424 | 0.657925053 | 0.521550319 | 0.486078147 |
| LOC409810 | 6.828059037 | 6.136913436 | 5.06288186 | 5.695537177 |
| LOC551987 | 55.59687006 | 42.19234438 | 40.41481098 | 45.76166237 |
| LOC551734 | 7.414221917 | 7.060398625 | 5.107420685 | 6.052733884 |
| LOC551838 | 11.24468034 | 10.62710375 | 7.492838187 | 9.106693849 |
| LOC552746 | 12.42212683 | 12.80871222 | 19.44795456 | 15.75762747 |
| LOC409913 | 14.02935429 | 20.07428465 | 24.97577973 | 21.10650508 |
| LOC409675 | 344.2618263 | 426.0899918 | 518.9991778 | 431.4381934 |
| LOC409622 | 55.46108025 | 68.81369335 | 76.42246546 | 70.03814482 |
| LOC724241 | 21.22323096 | 24.4554826 | 31.62430102 | 27.56507058 |
| LOC412209 | 12.06724639 | 22.11696222 | 22.46798529 | 17.08243704 |
| LOC552727 | 52.95236981 | 56.77177878 | 67.41290872 | 63.75171663 |
| LOC102654069 | 4.779570536 | 6.261146177 | 2.666070895 | 3.550855889 |
| LOC410749 | 4.861085291 | 4.533520602 | 4.066367008 | 3.935477075 |
| LOC551528 | 81.58208225 | 81.44922558 | 101.9750625 | 101.584389 |
| Or115 | 2.400240018 | 1.701651842 | 1.512998139 | 1.54410758 |
| - | 0.739117352 | 0.831410665 | 0.816733291 | 1.146103834 |
| LOC724467 | 22.65813909 | 29.83102402 | 34.76298853 | 30.19112733 |
| LOC408986 | 78.64990351 | 82.31553016 | 101.3325007 | 100.2964727 |
| LOC100577635 | 0.945603036 | 1.17442721 | 1.14568242 | 1.254403358 |
| LOC408562 | 6.19422291 | 5.744179779 | 5.335254123 | 5.284876323 |
| - | 0.183919516 | 0.546982436 | 0.522878747 | 0.562592352 |
| LOC408319 | 4.664597791 | 3.40258668 | 3.394561999 | 3.517848882 |
| LOC725086 | 35.05096365 | 37.53532412 | 44.67472204 | 44.83115894 |
| LOC102655712 | 0.327237781 | 0.335252941 | 0.559187131 | 0.486361393 |
| LOC551526 | 8.137607485 | 7.779062015 | 6.230867883 | 6.424407542 |
| LOC552691 | 3.844056381 | 2.99631339 | 3.429604697 | 3.026777083 |
| - | 1.564831078 | 1.423858344 | 1.00313671 | 1.248511965 |
| LOC408810 | 0.888548796 | 1.255814941 | 1.311651517 | 1.308511645 |
| LOC551706 | 0.520311648 | 0.887357466 | 0.614835588 | 0.706670497 |
| LOC725308 | 7.850341775 | 9.171956014 | 11.4613819 | 11.27507196 |
| LOC726187 | 53.19505746 | 53.16607933 | 43.82352153 | 46.20545799 |
| LOC726432 | 9.796063943 | 7.623472563 | 7.480125073 | 7.796030729 |
| CPR27 | 48.18844046 | 66.63933741 | 65.77033324 | 64.51291056 |
| LOC409777 | 0.628898023 | 0.544411356 | 0.607731226 | 0.394807301 |
| LOC408276 | 11.07489938 | 13.56804969 | 12.65770313 | 13.92671995 |
| LOC102653764 | 18.53230735 | 35.06476348 | 22.93814785 | 24.14688405 |
| LOC409514 | 29.07565701 | 25.41451869 | 24.33565614 | 24.26026664 |
| LOC100577365 | 0.072957095 | 0.147559756 | 0.256995036 | 0.154115583 |
| LOC411054 | 3.359629839 | 4.668415729 | 5.680028527 | 4.887897448 |
| LOC551168 | 2.250982026 | 2.290388784 | 1.924422512 | 1.73442926 |
| LOC100576803 | 0.186488624 | 0.278380814 | 0.210051576 | 0.367660876 |
| LOC725479 | 0.473013582 | 0.418557985 | 0.314983073 | 0.355593039 |
| LOC408458 | 7.412350353 | 8.399307573 | 10.73034627 | 9.244144318 |
| LOC409365 | 9.913110467 | 7.765307692 | 7.751940495 | 7.872185529 |
| LOC100577582 | 16.19219047 | 20.49662306 | 26.89561543 | 20.86679133 |
| LOC100577254 | 2.05173728 | 1.73281709 | 1.783557037 | 1.734341584 |
| LOC409841 | 13.16462963 | 15.73270058 | 16.46518856 | 16.01214685 |
| LOC100578417 | 1.033700565 | 0.667157122 | 0.582759408 | 0.673918279 |
| LOC100576265 | 0.924660105 | 0.467784315 | 0.408268089 | 0.46191506 |
| Gycbeta1 | 20.10310771 | 21.29143242 | 21.48703139 | 24.25147243 |
| LOC100576216 | 2.800240441 | 2.084922964 | 2.100316781 | 2.097995488 |
| LOC100577766 | 2.211978868 | 1.738913523 | 1.531767149 | 1.591644174 |
| LOC411766 | 2.913060466 | 2.316190159 | 2.168453529 | 2.251584264 |
| LOC551046 | 0.999329115 | 2.282282232 | 2.385516466 | 1.695850226 |
| - | 1.3065369 | 0.895553706 | 0.841076041 | 0.775620094 |
| LOC102654610 | 31.05104879 | 36.91316386 | 38.19099559 | 42.20118444 |
| LOC100578943 | 20.79758498 | 28.2835491 | 47.27830981 | 31.99373897 |
| LOC552447 | 114.2647035 | 187.4615565 | 215.4249662 | 173.282134 |
| LOC724802 | 27.1238143 | 32.84596759 | 39.82547568 | 39.03771912 |
| LOC100576888 | 2.670164406 | 2.793324443 | 3.491287215 | 3.85034523 |
| LOC727146 | 31.61093154 | 42.14868865 | 38.90655585 | 40.23213684 |
| LOC551928 | 5.660667555 | 5.323717818 | 4.37624339 | 4.625823731 |
| LOC412763 | 7.600733569 | 10.01136738 | 9.943267647 | 10.22497429 |
| LOC725459 | 14.14154757 | 17.27513239 | 22.30728494 | 17.79135183 |
| LOC551623 | 1.92451658 | 2.985293808 | 2.491514359 | 3.065251426 |
| LOC413650 | 6.841561363 | 6.600749293 | 4.784055732 | 5.839084661 |
| LOC412024 | 7.731264767 | 6.174578356 | 6.139452899 | 6.242557075 |
| LOC102655112 | 4.71046917 | 4.271681832 | 3.916574994 | 3.712068907 |
| LOC726737 | 0.477486436 | 1.92266469 | 1.384733899 | 0.977902064 |
| LOC724223 | 8.752324983 | 7.919954299 | 6.110272361 | 7.149816172 |
| LOC102656215 | 8.99978235 | 11.17150529 | 12.23820618 | 11.46156191 |
| LOC409221 | 3.377777012 | 3.230896929 | 2.528461446 | 2.540668165 |
| LOC100578937 | 12.11235921 | 9.579871802 | 10.31600538 | 10.01727099 |
| LOC726013 | 556.7488211 | 736.5289506 | 865.5573111 | 771.0331992 |
| LOC113219326 | 5.726529974 | 7.097593397 | 6.067218775 | 9.019293873 |
| LOC413137 | 336.5501742 | 541.0902814 | 708.1753448 | 518.5044127 |
| LOC550663 | 9.795535421 | 12.85267582 | 16.27762111 | 12.50618521 |
| LOC551107 | 699.2213904 | 935.3665667 | 1151.444037 | 943.8079518 |
| LOC408626 | 10.52946795 | 12.2133277 | 14.0557682 | 13.40301784 |
| LOC102653960 | 15.63386045 | 17.61479372 | 23.84848883 | 21.19863232 |
| LOC551313 | 81.3300729 | 98.41042917 | 94.68449014 | 97.05981667 |
| LOC413837 | 34.87521192 | 36.31203625 | 23.87311956 | 28.6150116 |
| LOC102654889 | 1.247158506 | 1.230002159 | 1.171458688 | 0.883291624 |
| LOC412289 | 32.2916925 | 34.39963511 | 44.41015492 | 40.39054875 |
| LOC410331 | 1.581746379 | 1.616209622 | 1.538115186 | 1.249100718 |
| - | 15.58279991 | 21.17732899 | 20.9038546 | 20.54627494 |
| LOC726543 | 1.159585383 | 1.822274559 | 1.354533799 | 2.121896909 |
| LOC413625 | 11.25572608 | 10.12344622 | 8.707174042 | 9.083358876 |
| LOC100576273 | 0.212202367 | 0.177947607 | 0.079915145 | 0.083618589 |
| LOC408762 | 2.662947138 | 1.615211219 | 1.513255851 | 1.849991857 |
| LOC410030 | 25.87218802 | 42.13142311 | 58.64517095 | 40.29830526 |
| LOC551179 | 1.738422945 | 2.050033663 | 2.67093936 | 2.887145371 |
| LOC726200 | 1.005781475 | 1.180483366 | 1.078573129 | 1.446489639 |
| LOC727298 | 3.51805248 | 2.808282879 | 2.623288546 | 2.715731112 |
| LOC725026 | 8.296209491 | 15.48772066 | 17.74949203 | 14.87045341 |
| LOC413845 | 91.01844047 | 110.3823539 | 120.5604962 | 111.1469324 |
| LOC727035 | 0.327231432 | 0.115324295 | 0.186162605 | 0.10962882 |
| LOC552818 | 11.79673847 | 11.65552278 | 8.429134396 | 9.572234953 |
| LOC412806 | 18.20344741 | 25.01877623 | 32.85673023 | 24.59065052 |
| LOC412309 | 5.251730674 | 8.472076073 | 8.512616186 | 6.992680393 |
| LOC725941 | 2.464844428 | 1.737627604 | 2.137428434 | 1.665967905 |
| LOC410015 | 21.00235963 | 20.18708305 | 17.33856147 | 18.08438246 |
| LOC726433 | 0.846180902 | 0.573610978 | 0.554269976 | 0.599685197 |
| LOC102655407 | 140.1751523 | 131.2512853 | 121.7311744 | 123.1315582 |
| LOC408969 | 37.50715578 | 53.07805283 | 44.49239258 | 44.80593071 |
| LOC411870 | 13.5945308 | 17.26560704 | 17.85164175 | 17.18129959 |
| LOC550684 | 9.555427231 | 9.75091755 | 12.46902857 | 12.35873804 |
| LOC551587 | 7.872611504 | 11.21523202 | 14.77703143 | 11.03239835 |
| LOC551109 | 1.23832126 | 0.72096205 | 0.910401622 | 0.833596204 |
| LOC409521 | 20.35379033 | 22.51246911 | 29.48094503 | 24.6455568 |
| LOC725455 | 1.817608298 | 1.991756552 | 2.144583646 | 2.570482005 |
| LOC410206 | 12.19215933 | 10.10560893 | 9.396490572 | 9.615729789 |
| LOC100578894 | 1.822266014 | 1.095190052 | 0.964579688 | 1.05985477 |
| LOC113219014 | 4.769210383 | 6.157620178 | 6.06262514 | 6.496791504 |
| LOC726740 | 19.50589166 | 20.82965015 | 22.97761542 | 25.25516269 |
| LOC552355 | 9.140996442 | 7.368191474 | 6.55362454 | 7.546312013 |
| LOC551680 | 12.0178035 | 13.38335245 | 15.11077342 | 14.49164089 |
| - | 7.56541782 | 4.54281236 | 6.136469109 | 4.747735442 |
| LOC102654086 | 0.036338956 | 0.160512727 | 0.027615711 | 0.183462711 |
| LOC551941 | 4.129694226 | 6.579758825 | 5.591993127 | 5.453204987 |
| LOC727309 | 74.38544966 | 84.6617786 | 103.7326779 | 92.66003021 |
| Grd | 9.622534742 | 9.110994407 | 7.265386968 | 7.870723075 |
| LOC100576671 | 5.124436201 | 6.830645666 | 10.53614455 | 7.695386645 |
| LOC552399 | 2.427549931 | 1.916801654 | 1.775464226 | 1.741444113 |
| LOC410487 | 4.298840983 | 5.766275966 | 5.073070398 | 5.500923955 |
| LOC409728 | 562.8587312 | 906.5307543 | 1070.465548 | 851.3444537 |
| LOC726616 | 80.23305037 | 70.17433186 | 59.96391557 | 67.32130631 |
| LOC412284 | 7.775235491 | 7.088771477 | 5.84774714 | 6.239880931 |
| LOC107965950 | 3.143301705 | 2.874270751 | 2.865139529 | 2.022256336 |
| LOC412349 | 45.384003 | 48.81206205 | 61.68418645 | 56.62720706 |
| LOC100577816 | 5.819778381 | 4.232486401 | 4.058927452 | 4.665952718 |
| LOC726167 | 14.48256979 | 16.43257902 | 19.87055324 | 17.74520841 |
| LOC552064 | 6.804646604 | 5.841533513 | 5.580770251 | 5.664084636 |
| LOC413451 | 1.97624462 | 1.37139299 | 1.661408798 | 1.50536681 |
| LOC552460 | 41.17539046 | 70.17197192 | 69.90496854 | 62.42545835 |
| LOC413073 | 9.9219327 | 9.934758089 | 7.57381287 | 8.430996465 |
| LOC551282 | 18.48253583 | 26.75474491 | 28.58517233 | 23.53181213 |
| LOC409286 | 15.73037054 | 18.52858292 | 20.79698328 | 20.45607799 |
| LOC552715 | 12.63569349 | 14.24966903 | 19.84823522 | 15.80503294 |
| LOC411802 | 1.325025901 | 1.200702851 | 1.195230565 | 1.008612884 |
| LOC551903 | 16.81433191 | 8.298644663 | 8.331958873 | 11.76295731 |
| LOC413021 | 3.938898154 | 5.879879991 | 5.917943485 | 5.258018388 |
| LOC726839 | 0.31003229 | 0.578429174 | 0.897836486 | 0.593371406 |
| LOC412076 | 12.04229272 | 10.00008653 | 10.83010869 | 9.964636474 |
| LOC413873 | 0.974578152 | 0.961612377 | 0.706496409 | 0.734716418 |
| LOC725009 | 2.812776886 | 1.750279113 | 1.965377746 | 2.049767839 |
| - | 1.012604824 | 0.763465328 | 0.564034555 | 0.698368958 |
| LOC408574 | 8.078129815 | 7.256357678 | 5.963085653 | 6.809567202 |
| LOC413649 | 3.673354581 | 3.715979156 | 4.456075744 | 4.919376191 |
| LOC100577607 | 25.11662584 | 32.43154264 | 36.1358767 | 30.31012915 |
| LOC552225 | 8.631226998 | 6.228889799 | 7.050785468 | 6.409258562 |
| Tspan6 | 97.82739537 | 152.8397304 | 152.5767338 | 127.8557378 |
| LOC725659 | 45.30167618 | 47.68366711 | 59.93037881 | 55.29262381 |
| LOC725796 | 10.86354982 | 11.7453547 | 13.87217763 | 13.8870508 |
| LOC100578770 | 5.948486242 | 6.687611567 | 7.519137498 | 9.282062785 |
| LOC724233 | 516.3872958 | 830.5989627 | 1054.048033 | 767.5604821 |
| LOC410613 | 16.93199559 | 20.08493622 | 20.62626267 | 23.5863345 |
| LOC551235 | 14.2779418 | 23.37584647 | 31.83443021 | 23.95205727 |
| LOC411217 | 1.562914568 | 0.982573333 | 1.317979244 | 1.211848479 |
| - | 0.140899521 | 0.307761844 | 0.338417252 | 0.511701812 |
| LOC100579019 | 0.581344195 | 0.729253847 | 0.980678773 | 1.386357531 |
| LOC552725 | 1.486624486 | 1.284915283 | 1.271932321 | 1.046011644 |
| LOC726379 | 5.103751665 | 4.331684523 | 5.347060624 | 3.966272234 |
| LOC411269 | 17.03368117 | 28.41797365 | 37.80830073 | 27.79708722 |
| LOC408426 | 9.405163551 | 10.17424827 | 13.82761845 | 12.69599171 |
| LOC551617 | 12.25512623 | 13.86877862 | 16.82842215 | 16.44015489 |
| LOC107965706 | 69.40325268 | 74.57736059 | 79.69291875 | 86.2580413 |
| LOC725268 | 7.09897622 | 5.060171564 | 5.459283164 | 5.537565452 |
| LOC406093 | 7158.991734 | 25411.97486 | 28927.80404 | 34772.88501 |
| LOC102656294 | 1.784674494 | 2.764273201 | 2.40716117 | 2.862789551 |
| LOC410556 | 1V3.20010748 | 9.708186287 | 8.740573693 | 10.29397053 |
| LOC552806 | 3.066283683 | 2.241299443 | 2.023780209 | 2.276912256 |
| LOC408867 | 5.674961849 | 7.714359265 | 6.766529993 | 7.23466315 |
| LOC724240 | 3.801514083 | 2.317912492 | 1.990118455 | 2.725123514 |
| LOC411053 | 0.212791113 | 0.175202665 | 0.167846561 | 0.124456832 |
| LOC727126 | 2.636547923 | 6.303121389 | 5.837363274 | 4.176965897 |
| LOC413544 | 6.838901659 | 6.219828689 | 5.446357742 | 5.459025506 |
| LOC408650 | 85.44806774 | 96.0853123 | 95.35510156 | 120.3261187 |
| LOC552800 | 2.975869947 | 2.802046392 | 2.577318915 | 2.331186932 |
| LOC411695 | 87.39964213 | 111.3912977 | 140.272831 | 114.0440765 |
| For | 9.516303368 | 17.42200933 | 20.54552007 | 15.78831588 |
| - | 0.177544719 | 0.094573952 | 0.060562967 | 0.047525645 |
| LOC100578289 | 0.899740244 | 0.481893154 | 0.163735936 | 0.383286385 |
| LOC551839 | 33.92065205 | 40.80327682 | 50.92036664 | 44.89378988 |
| LOC411983 | 9.605611961 | 22.10764984 | 18.06098925 | 15.83070644 |
| LOC726273 | 10.05603568 | 10.02968208 | 7.776464291 | 8.370507671 |
| LOC409016 | 20.16340531 | 23.95587618 | 25.99247206 | 25.99274873 |
| LOC550920 | 5.197131814 | 4.152994709 | 4.404516115 | 3.982825833 |
| LOC726789 | 856.0188966 | 992.7124418 | 1230.079292 | 1111.244647 |
| LOC408673 | 12.23186133 | 14.00798777 | 17.8497993 | 16.03615897 |
| LOC726633 | 7.528020547 | 7.105328087 | 6.597330799 | 6.322376496 |
| LOC724310 | 1.289072823 | 1.849603429 | 2.036440524 | 1.79305843 |
| LOC412935 | 7.315847648 | 7.24331967 | 6.891842891 | 6.18883997 |
| LOC725376 | 2.653855118 | 2.015015415 | 1.985933951 | 2.079800408 |
| LOC412808 | 10.18034772 | 13.67983643 | 14.0354914 | 13.85873509 |
